# Optical Resolution of Carboxylic Acid Derivatives of Homoleptic Cyclometalated Iridium(III) Complexes via Diastereomers Formed with Chiral Auxiliaries

**DOI:** 10.1021/acs.inorgchem.3c00685

**Published:** 2023-07-11

**Authors:** Azusa Kanbe, Kenta Yokoi, Yasuyuki Yamada, Makoto Tsurui, Yuichi Kitagawa, Yasuchika Hasegawa, Daiji Ogata, Junpei Yuasa, Shin Aoki

**Affiliations:** † Faculty of Pharmaceutical Science, Tokyo University of Science, 2641 Yamazaki, Noda, Chiba 278-8510, Japan; ‡ Department of Chemistry, Graduate School of Science, Nagoya University, Furo-cho, Chikusa-ku, Nagoya 464-8602, Japan; § Research Center for Materials Science, Nagoya University, Furo-cho, Chikusa-ku, Nagoya 464-8602, Japan; ∥ JST, PRESTO, 4-1-8 Honcho, Kawaguchi, Saitama 332-0012, Japan; ⊥ Graduate School of Chemical Sciences and Engineering, Hokkaido University, N13W8, Kita-ku, Sapporo, Hokkaido 060-8628, Japan; # Faculty of Engineering, Hokkaido University, Kita-13, Nishi-8, Kita-Ku, Sapporo, Hokkaido 060-8628, Japan; ∇ Institute for Chemical Reaction Design and Discovery (WPI-ICReDD), Hokkaido University, Kita-21, Nishi-10, Kita-Ku, Sapporo, Hokkaido 001-0021, Japan; ○ Faculty of Science, Tokyo University of Science, 1-3 Kagurazaka, Shinjuku-ku, Tokyo 162-8601, Japan; ● Research Institute for Science and Technology (RIST), Tokyo University of Science, 2641 Yamazaki, Noda, Chiba 278-8510, Japan; □ Research Institute for Biomedical Science (RIBS), Tokyo University of Science, 2641 Yamazaki, Noda, Chiba 278-8510, Japan

## Abstract

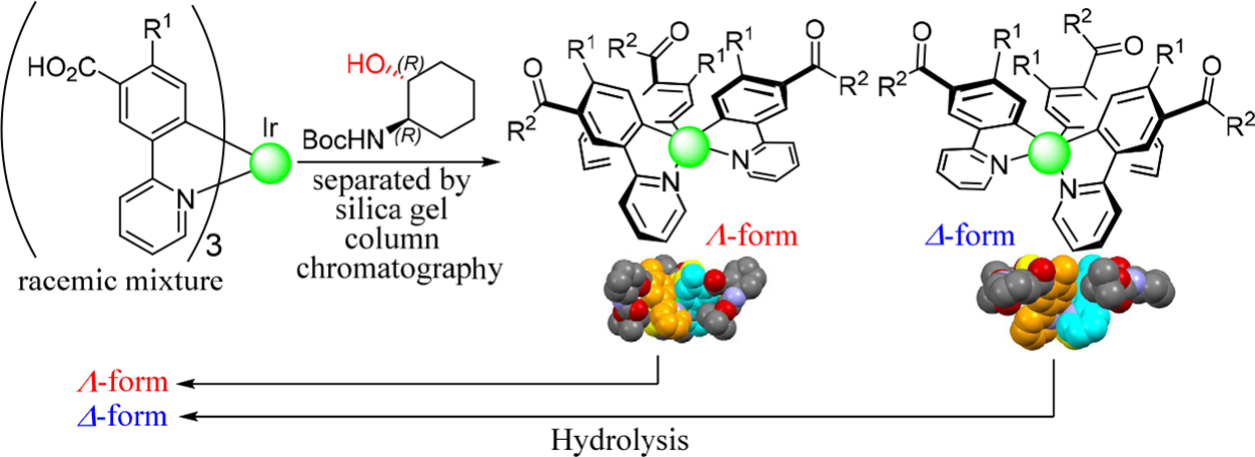

We report on a facile method for the optical resolution of cyclometalated iridium(III) (Ir(III)) complexes via diastereomers formed with chiral auxiliaries. The racemic carboxylic acids of Ir(III) complexes (*fac*-**4** (*fac*-Ir(ppyCO_2_H)_3_ (ppy: 2-phenylpyridine)), *fac*-**6** (*fac*-Ir(tpyCO_2_H)_3_ (tpy: 2-(4′-tolyl)pyridine)), and *fac*-**13** (*fac*-Ir(mpiqCO_2_H)_3_ (mpiq: 1-(4′-methylphenyl)isoquinoline))) were converted into the diastereomers, Δ- and Λ-forms of *fac*-**9** (from *fac*-**6**), *fac*-**10** (from *fac*-**4**), *fac*-**11** (from *fac*-**6**), and *fac*-**14** (from *fac*-**13**), respectively, by the condensation with (1*R,*2*R*)-1,2-diaminocyclohexane or (1*R,*2*R*)-2-aminocyclohexanol. The resulting diastereomers were separated by HPLC (with a nonchiral column) or silica gel column chromatography, and their absolute stereochemistry was determined by X-ray single-crystal structure analysis and CD (circular dichroism) spectra. Spectra of all diastereomers of the Ir(III) complexes are reported. Hydrolysis of the ester moieties of Δ- and Λ-forms of *fac*-**10**, *fac*-**11**, and *fac*-**14** gave both enantiomers of the corresponding carboxylic acid derivatives in the optically pure forms, Δ-*fac* and Λ-*fac*-**4**, -**6**, and -**13**, respectively.

## Abstract 2

We report on an efficient method for the optical resolution of carboxylic acids of the homoleptic cyclometalated iridium(III) (Ir(III)) complexes via diastereomeric amides and esters formed by the condensation with (1*R,*2*R*)-1,2-diaminocyclohexane and (1*R,*2*R*)-2-aminocyclohexanol, respectively. The resulting diastereomers were separated by HPLC or silica gel column chromatography, and their absolute stereochemistry was determined by X-ray single-crystal structure analysis and CD (circular dichroism) spectra. Hydrolysis of the ester moieties of Δ- and Λ- *fac*-**10**, *fac*-**11**, and *fac*-**14** gave the corresponding carboxylic acid derivatives in the optically pure forms.

## Introduction

Homoleptic cyclometalated Ir(III) complexes such as facial *fac-*Ir(tpy)_3_ (tpy: 2-(4′-tolyl)pyridine) and *fac*-Ir(ppy)_3_ (ppy: 2-phenylpyridine) have *C*_3_-symmetric structures and exhibit excellent photophysical properties, large Stokes shifts, high quantum yields, and long lifetimes for their luminescence emission. Because of their attractive photochemical properties, considerable interest has developed in applications for not only organic light-emitting diodes (OLEDs) as phosphorescent emitters,^1^ but also bioimaging probes,^2^ pH sensors,^3^ anticancer reagents,^4^ photoredox catalysts,^5^ and related applications.^6^

In our group, regioselective electrophilic reactions at the 5′-position of phenylpyridine units and subsequent conversions of tris cyclometalated Ir(III) complexes, a process that is referred to as “post-complexation functionalization (PCF)”, were developed for the synthesis of a series of Ir(III) complexes that exhibit blue, green, and red emissions.^7^ For example, Ir complexes functionalized with amino and pyridyl groups exhibit emission color changes between green and red due to the protonation and deprotonation of their basic groups in aqueous solutions.^8^ We conducted the preparation of Ir(III) complexes bearing biologically active peptides for therapy and the luminescent imaging of cancer cells.^9,10^ It was reported that the hybrid compounds of the Ir(III) complex with cyclic peptides (Ir(III) complex-peptide hybrids, IPHs) bind to death receptors (DRs) that are expressed on cancer cells,^9^ and those with cationic peptides containing lysine (K) and glycine (G)^10^ induce programmed cell death (PCD) such as apoptosis, necroptosis, and paraptosis in cancer cells. A number of other Ir(III) complexes that exhibit red, green, blue, and white colorless luminescence emission and that have long emission lifetimes were also synthesized.^7,11,12^

Intrinsically, Ir(III) complex cores adopt 6-coordinate octahedral or pseudo-octahedral structures and hence possess metal-centered chirality, for example, delta (Δ) and lambda (Λ) forms.^13,14^ There are only limited methods currently available for separating these metal-centered enantiomers, one of which involves the use of chiral HPLC columns for the separations of several homoleptic and heteroleptic complexes^15,16^ and acetylacetone (acac) complexes.^17^ A method for synthesizing a five-coordinate Ir(III) complex and separating the resulting enantiomers by chiral HPLC followed by conversion to heteroleptic complexes has also been reported, in which enantiomers of Ir(III) complexes are separated by means of an HPLC chiral stationary phase using amylose or cellulose derivatives.^18^

**Chart 1:**
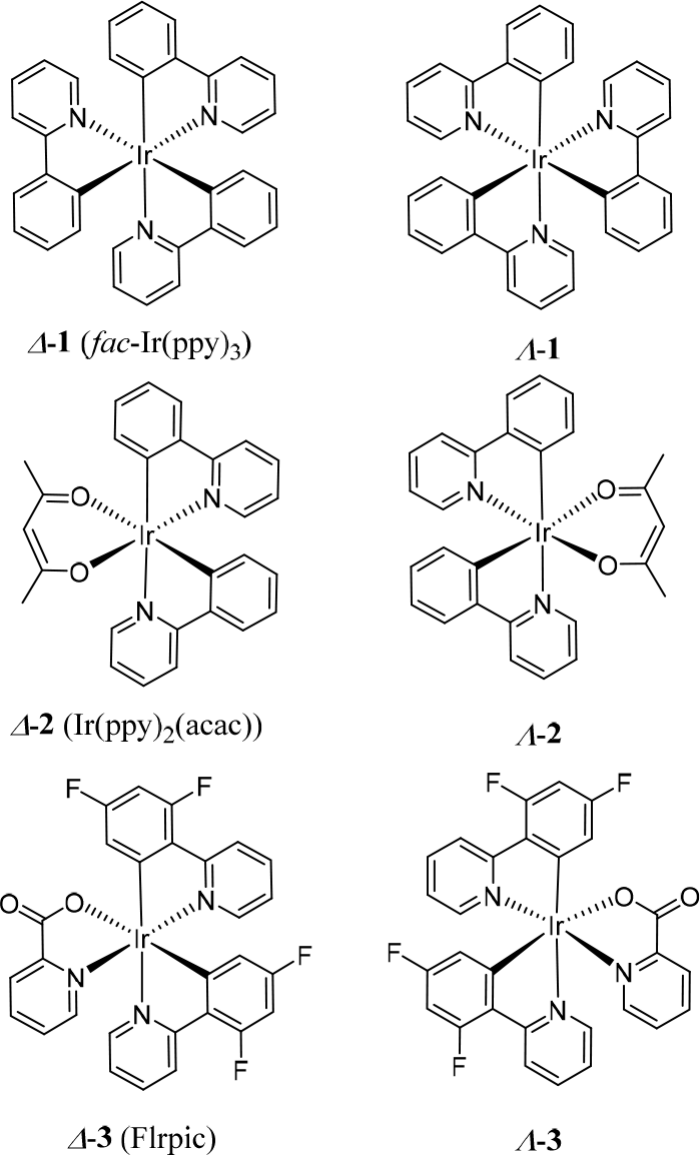
Chemical Structures and Absolute Configuration of Representative Cyclometalated Ir(III) Complexes

Meggers’ group reported the separation of Λ- and Δ-isomers of Ir(III) complexes by introducing chiral ligands such as (*S*)-4-*tert*-butyl-2-(2′-hydroxyphenyl)-2-oxazoline analogues, l-proline, and l-α-methylproline.^19^ Similar methods for the enantioselective synthesis of bis-heteroleptic Ir complexes were carried out via the complexation with l- and d-serine.^20^ Zuo and co-workers reported on the separation of **1**–**3** by chiral HPLC columns and the CD (circular dichroism) and CPL (circularly polarized luminescence) spectra of the purified enantiomers (Chart 1). They also conducted the complexation of the μ-complex {Ir(dfppy)_2_(μ-Cl)}_2_ (dfppy: 4,6-difluorophenylpyridine) with optically pure ancillary ligands, (*R*)- and (*S*)-2-(4-ethyl-4,5-dihydrooxazol-2-yl)phenol (edp), to obtain four enantiomeric Ir(III) complexes, Λ- and Δ-forms of Ir(dfppy)_2_(*R*-edp) and Ir(dfppy)_2_(*S*-edp).^21^ A more direct method includes the diastereoselective formation of the *fac*-Λ-complex from a tripodal ligand having three optically pure cyclometalating ligand units, 2-(2′-phenyl)-4,5-pinenopyridine (pppy), and a separation with a silica preparative plate.^22^

**Chart 2:**
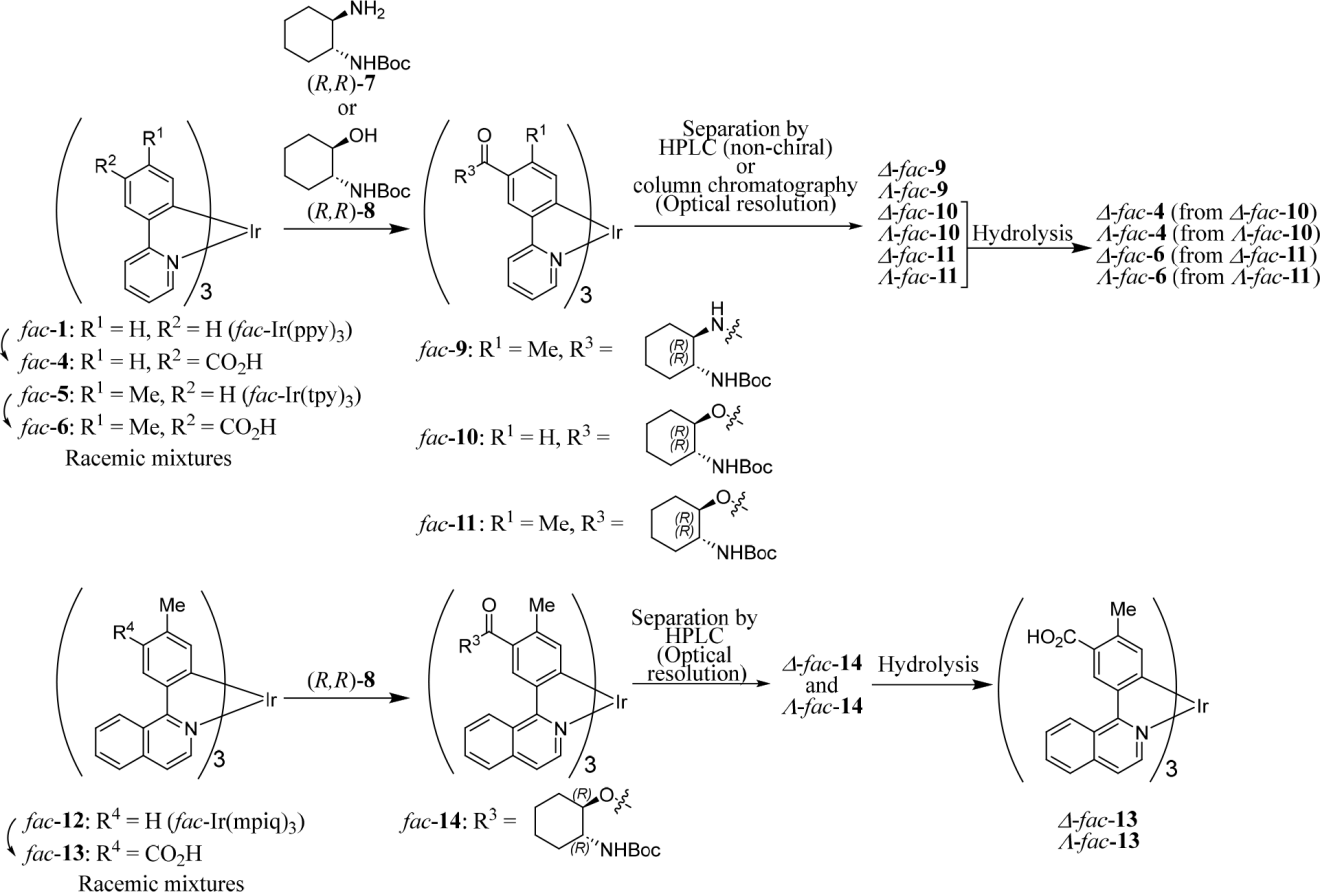
Optical Resolution of Cyclometalated Ir(III) Complexes

This background has prompted us to conduct the optical resolution of homoleptic cyclometalated Ir(III) complexes that contain carboxylic acid moieties, which are important products and intermediates of the postcomplexation functionalization,^7^ via their corresponding diastereomeric intermediates to produce optically pure Ir(III) complexes. In this work, we incorporate optically active diamine or amino alcohol units into the complexes to convert the two enantiomers to diastereomeric isomers in order to separate the Δ-forms and Λ-forms from one another (Chart 2). Namely, carboxylic acid derivatives of racemic mixtures, *fac*-**4** (*fac*-Ir(ppyCO_2_H)_3_), **6** (*fac*-Ir(tpyCO_2_H)_3_), and **13** (*fac*-Ir(mpiqCO_2_H)_3_, mpiq: 1-(4′-methylphenyl)isoquinoline), which were synthesized from *fac*-**1**, -**5**, and -**12** by PCF,^8a^ were converted into the corresponding diastereomers by introducing the chiral units (1*R*,2*R*)-1,2-diaminocyclohexane ((1*R,*2*R*)-1,2-DAH, (*R,R*)-**7**)^23^ and (1*R*,2*R*)-2-aminocyclohexanol ((1*R,*2*R*)-2-ACH, (*R,R*)-**8**)^24^ to give *fac*-**9**, *fac*-**10**, and *fac*-**11**, respectively (Chart 2). These stereoisomers were then separated by normal-phase HPLC or silica gel column chromatography to obtain Δ*-* and Λ*-fac-***9**, -**10**, and -**11**, respectively. Optically pure Δ- and Λ-*fac*-**4** and -**6** were then isolated by the hydrolysis of Δ*-* and Λ*-fac*-**10** and -**11**, respectively. In addition, *fac*-**13** was also converted to the two diastereomers of *fac*-**14**, which were converted to Δ*-fac-***13** and Λ*-fac*-**13** by a similar procedure (Chart 2). This method allows us to obtain both enantiomers at the same time on a large scale via the diastereomeric intermediates conjugated with one enantiomer of chiral auxiliary units.^25^

## Results and Discussion

### Design and Synthesis of Diastereomeric Amides (*fac*-**9**) and Esters (*fac*-**10**, -**11**, and -**14**)

Methods for the optical resolution of chiral organic carboxylic acids can generally be classified into the following methods: (i) diastereomeric salt formation with chiral amines, (ii) the formation of diastereomeric esters by forming covalent bonds with chiral alcohols, and (iii) kinetic resolution by enzymatic or nonenzymatic reactions (e.g., stereoselective hydrolysis of the corresponding racemic esters).^13^ We initially attempted to produce salts of *fac*-**6**^8a^ with optically active amines such as quinidine, cinchonidine, (*R*)-1-(1-naphthyl)ethylamine, l-arginine, (*R,R*)-diphenyldiaminoethane, and (1*R,*2*R*)-1,2-diaminocyclohexane in several solvents and carried out the recrystallization of the resulting salts. However, these efforts resulted in a negligible enrichment of enantiomers (data not shown).

**Chart 3:**
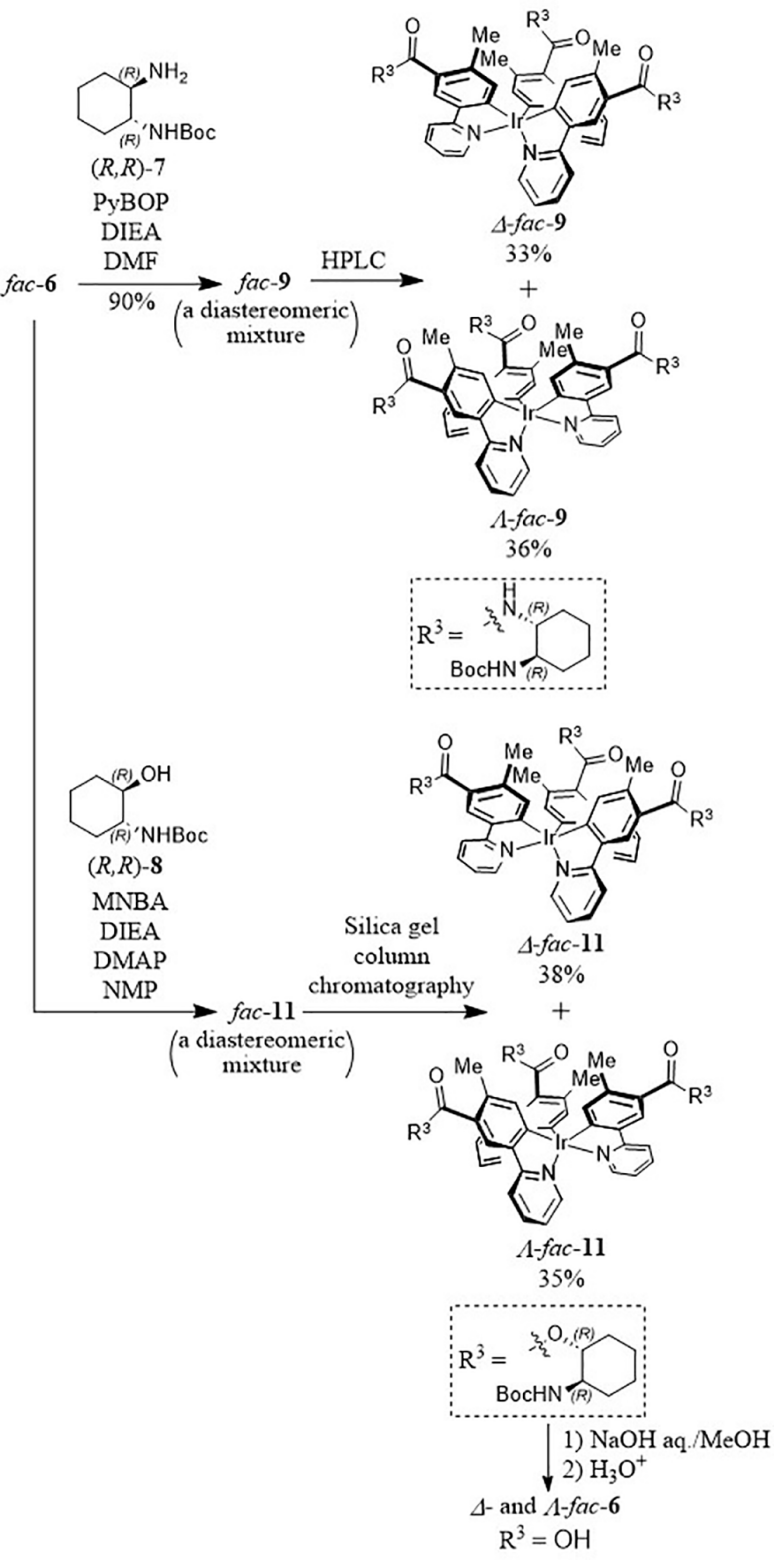
Synthesis of Optically Pure *fac*-**9** and *fac*-**6** from Racemic *fac*-**6**

Therefore, we synthesized diastereomeric esters of *fac*-**6** by reactions with optically pure alcohols such as (1*R,*2*S,*5*R*)-menthol, l-prolinol, and its derivatives for optical resolution. However, the separation of these diastereomers by nonchiral HPLC and/or column chromatography was also not successful (data not shown). We then conducted the condensation reactions of *fac*-**6** with (*R,R*)-**7** in the presence of benzotriazol-1-yloxytripyrrolidinophosphonium hexafluorophosphate (PyBOP) and *N*,*N*-diisopropylethylamine (DIEA) to obtain *fac*-**9** as a diastereomeric mixture, as shown in Charts 2 and 3 (top half). Separation using normal-phase and nonchiral HPLC gave Δ*-fac-***9** (33%) and Λ*-fac-***9** (36%). It should be noted here that the introduction of only one enantiomer of 1,2-DAH or 2-ACH ((*R,R*)-forms in this work) was sufficient to permit the Δ-forms and Λ-forms to be separated from one another. Note that Δ-*fac-***9** and Λ-*fac-***9** are actually Δ*-*(*R,R*)-*fac-***9** and Λ*-*(*R,R*)-*fac-***9**, respectively, because they contain three (1*R,*2*R*)-1,2-DAH units, and their nomenclature is abbreviated to Δ-*fac-***9** and Λ-*fac-***9** for clarity in this manuscript.

Next, the synthesis of *fac*-**11** from *fac*-**6** and (*R*,*R*)-**8** was attempted (Chart 2). The initial condensation reaction of *fac*-**11** with (*R,R*)-**8** using 2-methyl-6-nitrobenzoic anhydride (MNBA)^26^ gave *fac*-**11** as a diastereomeric mixture, which was separated by silica gel column chromatography to afford Λ*-* and Δ*-*forms of *fac*-**11** in 0.7% and 0.4% yields, respectively (Table 1, entry 1). Increasing the amount of MNBA and reaction time used in the reaction somewhat improved the chemical yields of the desired products (Table 1, entries 2 and 3). The use of polar solvents such as *N*-methylpyrrolidone (NMP) resulted in similar yields (Table 1, entries 4 and 7), and THF afforded even higher yields, possibly because of the higher solubility of *fac*-**6** in THF (Table 1, entry 8). The results shown in entries 8–14 of Table 1 suggest that carrying out the condensation reactions at higher temperature (entries 8–10 vs entry 7) and at high concentrations (entries 10 and 13 (Chart 3, bottom half) and 14 vs entry 12) gave higher chemical yields. Eventually, 150–160 mg of Λ-*fac-***11** and Δ-*fac-***11** were obtained in entry 13. The separated Δ-*fac-***11** and Λ-*fac-***11** were converted to Δ-*fac-***6** and Λ-*fac-***6**, respectively, by hydrolysis with aqueous NaOH in MeOH/H_2_O.

**Table 1:**
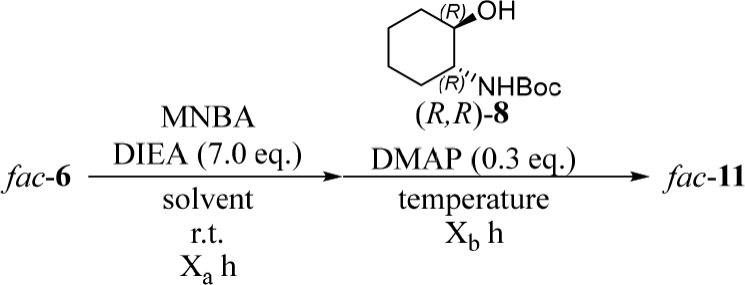
Examination of the Reaction Conditions for the Synthesis of *fac*-**11**

						time		yield (%)	
entry	(*R,R*)*-***8** (equiv)	MNBA (equiv)	solvent	concentration of **6** (M)	reaction temp	X_a_	X_b_	Λ-*fac-***11**	Δ-*fac-***11**
1	3.6	3.6	CH_2_Cl_2_	0.1	rt	10 min	27.5 h	0.7	0.4
2	7.1	3.6	CH_2_Cl_2_	0.1	rt	15 min	48 h	8	11
3	7.5	7.0	CH_2_Cl_2_	0.1	rt	25 min	120 h	4	3
4	3.6	3.6	MeCN	0.1	rt	30 min	96 h	2	2
5	3.8	3.6	CHCl_3_	0.1	rt	20 min	60 h	not obtained	
6	7.1	3.6	DMF	0.1	rt	15 h	41 h	4	2
7	6.3	3.6	NMP	0.1	40 °C	20 min	26 h	4	5
8	5.9	3.6	THF	0.05	reflux	10 min	19 h	9	14
9	7.1	3.6	NMP	0.1	60 °C	30 min	18 h	16	14
10^a^	7.1	3.6	NMP	0.1	80 °C	1.5 h	1 h	24	19
	3.1	3.5							
11^b^		3.7	NMP	0.1	100 °C	11 h		not obtained	
12	7.2	7.1	NMP	0.07	rt	10 min	13 h	6	3
13	7.1	3.6	NMP	0.3	80 °C	4 h	2 h	35	38
14	7.1	3.6	NMP	0.5	80 °C	4 h	2.5 h	30	35

a (*R,R*)*-***8** and MNBA were added to the reaction mixtures in two batches.

b The reaction was performed at 100 °C from the reaction to form the mixed anhydride in the first step.

*Fac*-**10** and *fac*-**14** were synthesized from *fac*-**4** and *fac*-**13** (Chart 2), respectively, by using MNBA and 2-fluoro-6-(trifluoromethyl)benzoic anhydride (FTFBA) (Chart 3 and Table 2). In both cases, better chemical yields were obtained when FTFBA was used in daylight (Table 2, entries 3 and 6 vs entries 1, 2, and 4). In the synthesis of *fac*-**14** using MNBA as a condensation reagent, the mixed anhydride intermediate was observed to decompose under daylight conditions (entries 4 and 5 in Table 2), possibly due to the decomposition of the anhydride intermediates, whose nitro groups may absorb UV–visible light. Fortunately, the use of FTFBA under daylight conditions afforded *fac*-**14** in moderate chemical yield (Table 2, entry 6).

**Table 2:**
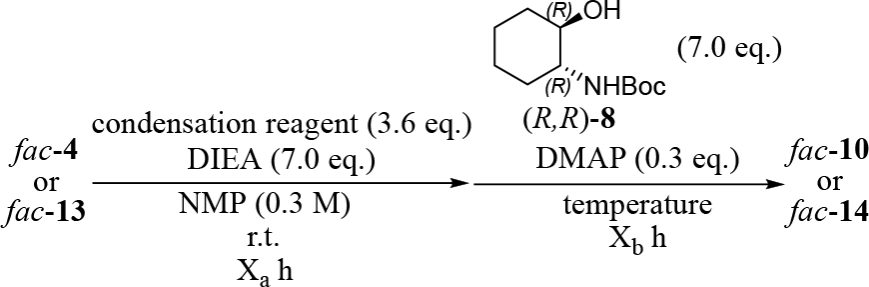
Syntheses of *fac*-**10** and *fac-***14** from *fac*-**4** and *fac*-**13**, Respectively

				time		products	
entry	substrate	condensation reagent	temp	X_a_	X_b_		
1	*fac-***4**	MNBA	80 °C	2.5 h	2 h	Λ-*fac-***10** (15%)	Δ-*fac-***10** (16%)
2	*fac-***4**	FTFBA	rt	15 min	5 h	Λ-*fac-***10** (16%)	Δ-*fac-***10** (14%)
3	*fac-***4**	FTFBA	80 °C	2.5 h	3 h	Λ-*fac-***10** (23%)	Δ-*fac-***10** (20%)
4^a^	*fac-***13**	MNBA		1 day		not obtained	
5^b^	*fac-***13**	MNBA	80 °C	30 min	3 h	Λ-*fac-***14** (19%)	Δ-*fac-***14** (23%)
6	*fac-***13**	FTFBA	80 °C	2 h	2.5 h	Λ-*fac-***14** (9%)	Δ-*fac-***14** (10%)

a Mixed anhydride intermediate was decomposed.

b The reaction was performed under dark conditions.

### X-ray Crystal Structures of Δ-*fac*-**9**, Δ-*fac*-**11**, and Λ-*fac*-**11**

**Figure 1:**
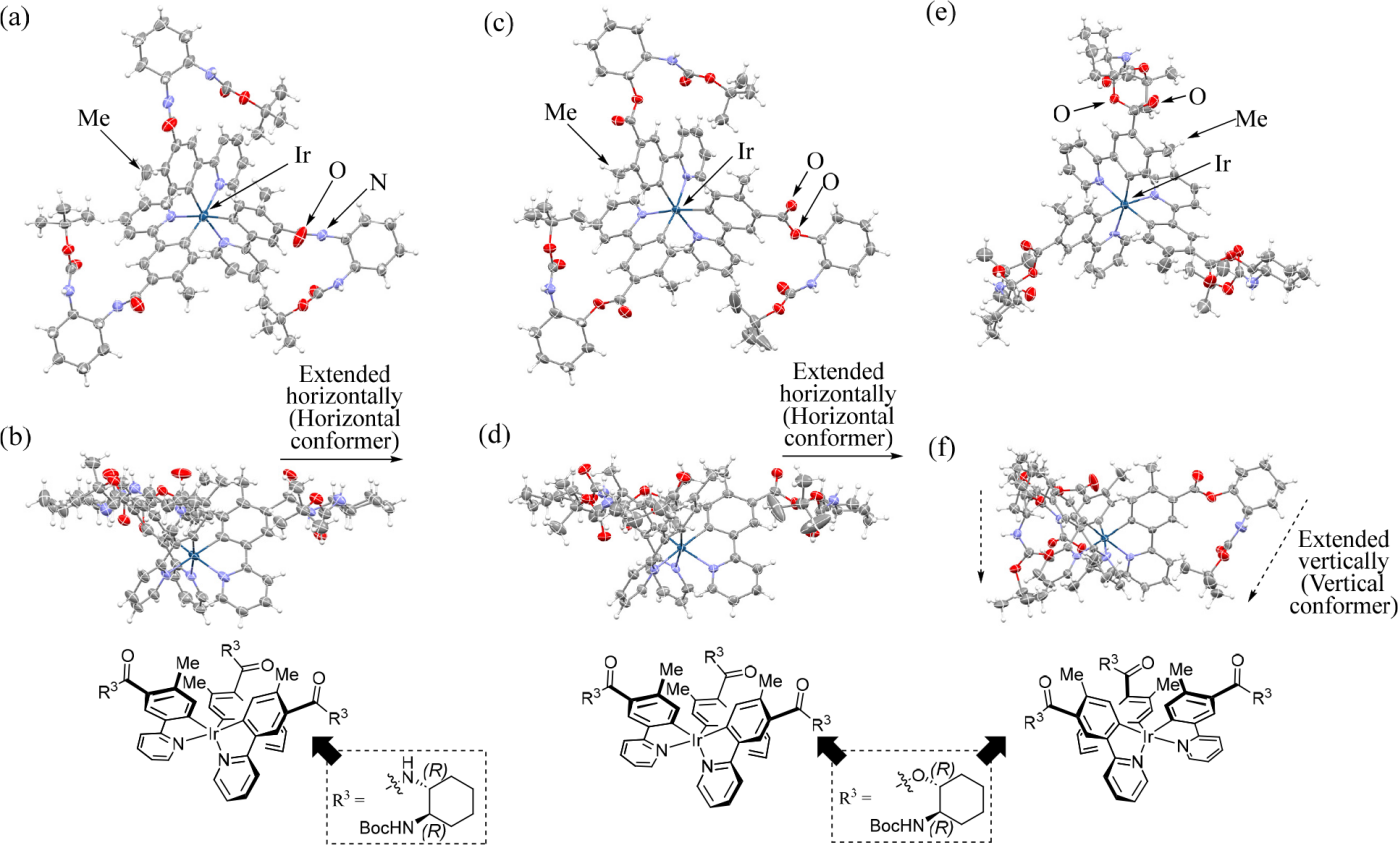
ORTEP drawings of single crystal structures of Δ-*fac-***9**, Δ-*fac-***11**, and Λ-*fac-***11**: (a, b) top and side views of Δ-*fac-***9** (horizontal conformer), (c, d) top and side views of Δ-*fac-***11** (horizontal conformer), and (e, f) top and side views of Λ-*fac-***11** (vertical confomer) with thermal ellipsoids (50% probability). For clarity, CHCl_3_ and hexane included in the crystal were omitted.

The absolute configurations of Δ-*fac-***9**, Δ-*fac-***11**, and Λ-*fac-***11** were determined by X-ray single-crystal structure analysis of the yellow crystals obtained by recrystallization from hexanes/CHCl_3_ as shown in Figure 1 (typical parameters of this X-ray crystal structure analysis are given in Table S1 in the Supporting Information). The average N(tpy)–Ir bond lengths for Δ-*fac-***9**, Δ-*fac-***11**, and Λ-*fac-***11** are 2.12, 2.14, and 2.13 Å, respectively, and the average C(tpy)–Ir bond distances are 2.00, 1.99, and 2.01 Å, respectively (Table S2 in the Supporting Information), which are consistent with other reported values for *fac*-**1**, *fac*-**5**, and analogues thereof.^8,11,27^

Figure 1 also shows the difference in the conformations of Δ-*fac-***9** and -**11** and Λ-*fac-***11**. Namely, the (1*R,*2*R*)-1,2-DAH groups and (1*R,*2*R*)-2-ACH groups in Δ-*fac-***9** and -**11** are extended horizontally with respect to the central Ir(III) cores, as indicated by the plain arrows in Figure 1b,d (horizontal conformer). In contrast, the three (1*R,*2*R*)-2-ACH groups in Λ-*fac-***11** are oriented vertically (slightly directed toward the pyridine rings of the Ir(III) cores) with respect to the Ir(tpy)_3_ core (vertical conformer), as indicated with dashed arrows in Figure 1f. It is likely that this difference is due to the relationship between the stereochemistry of the Ir(tpy)_3_ core (Δ*-* or Λ*-*form) and the (1*R,*2*R*)-1,2-DAH or (1*R,*2*R*)-2-ACH units.

We performed DFT calculations on Δ-*fac-***9**, Λ-*fac-***9**, Δ-*fac-***11**, and Λ-*fac-***11** in an attempt to predict the most stable structures. It should be mentioned that the calculations were initiated from different conformers of these complexes from the structures found in the X-ray crystal structure analysis (actually, initiated from conformers in which their (1*R,*2*R*)-1,2-DAH or (1*R,*2*R*)-2-ACH units are located between those in “horizontal conformers” and “vertical conformers”). The result of DFT calculations presented in Figure S1 in the Supporting Information indicates that the (1*R,*2*R*)-1,2-DAH or (1*R,*2*R*)-2-ACH units in Δ-*fac-***9** and Δ-*fac-***11** are extended horizontally, as indicated by the plain arrows in Figure S1a,b,e,f in the Supporting Information. In contrast, (1*R,*2*R*)-1,2-DAH and (1*R,*2*R*)-2-ACH units in Λ-*fac-***9** (crystal structure was not obtained) and Λ-*fac-***11** are oriented vertically, as indicated with the dashed arrows in Figure S1c,d,g,h in the Supporting Information. These estimated structures are similar to the aforementioned X-ray crystal structures displayed in Figure 1. The estimated energy levels of the HOMO and LUMO of Δ- and Λ-*fac*-**9** and -**11** are summarized in Figure S2 in the Supporting Information as well as that of *fac*-**5**.^11^ It was found that these energies are almost the same regardless of the direction of the chiral units. The Gibbs free energies of these most stable structures were found to be almost same between the Δ- and Λ-forms (Figure S3 in the Supporting Information). It is likely that different conformations between Δ-*fac-***11** (Figure 1c,d) (and Δ*-fac*-**9** (Figure 1a,b)) and Λ*-fac*-**11** (Figure 1e,f) is due to the packing effect in the crystals, while findings and applications of some different reactivities between these diastereomers in specific solvents might be our next work.^28^

### HPLC Analysis of Diastereomeric Amides and Esters

**Figure 2:**
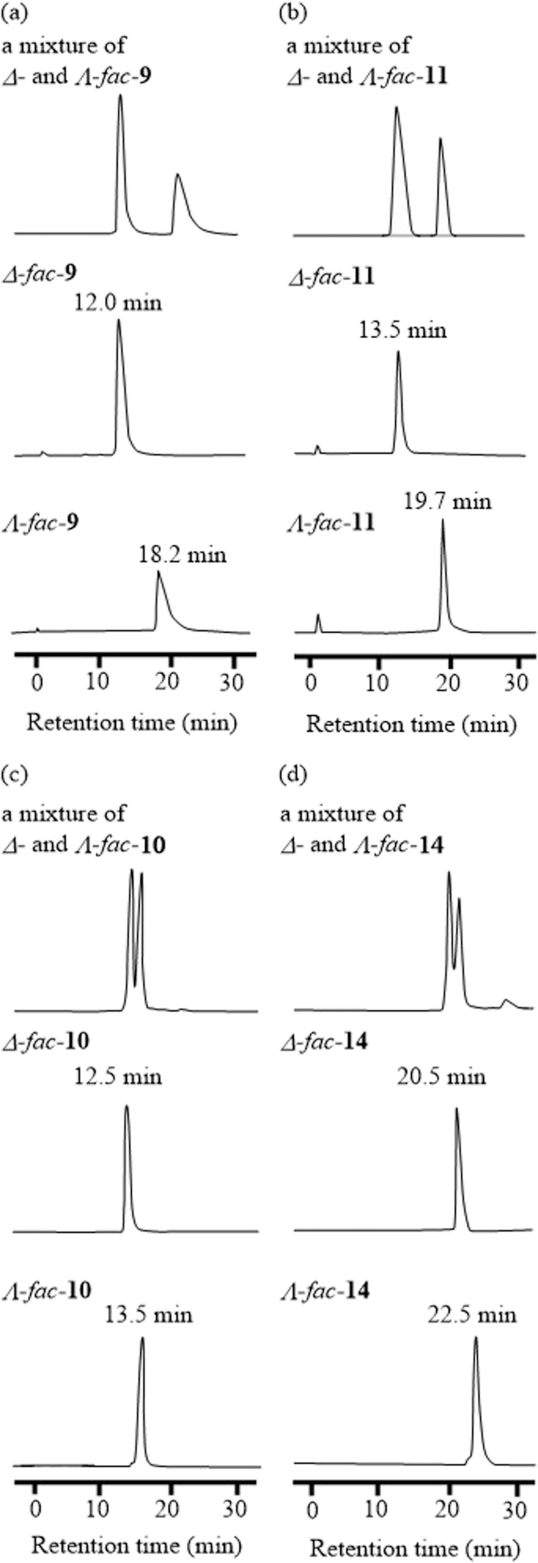
HPLC (normal-phase) chromatograms of diastereomeric amides and esters on SenshuPak PEGASIL Silica SP100 (achiral column). (a) HPLC chromatograms of a diastereomeric mixture of *fac*-**9** (top), Δ*-fac*-**9** (middle), and Λ-*fac-***9** (bottom). Eluent: CHCl_3_/MeCN = 1/2, flow rate 1.0 mL/min, UV detection at 254 nm. (b) HPLC chromatograms of Δ*-fac*-**11** (middle) and Λ-*fac-***11** (bottom). Eluent: hexanes/CHCl_3_ = 1/5, flow rate 1.0 mL/min, UV detection at 254 nm. (c) HPLC chromatograms of a diastereomeric mixture of *fac*-**10** (top), Δ*-fac*-**10** (middle), and Λ-*fac-***10** (bottom). Eluent: CHCl_3_ only, flow rate 1.0 mL/min, UV detection at 254 nm. (d) HPLC chromatograms of a diastereomeric mixture of *fac*-**14** (top), Δ*-fac*-**14** (middle), and Λ-*fac-***14** (bottom). Eluent: hexanes/CHCl_3_ = 1/2, flow rate 1.0 mL/min, UV detection at 254 nm.

Figure 2a,b shows typical HPLC chromatograms for *fac*-**9** and *fac*-**11** by normal-phase HPLC before (top) and after (middle and bottom) their separation by HPLC (normal phase) and silica gel column chromatography, respectively. Figure 2c,d shows the normal-phase (nonchiral) HPLC chromatograms for a mixture of **10** and **14** (top) and their Δ*-* and Λ*-*forms (middle and bottom, respectively) after the isolation, which confirm the purity of these diastereomers.

### ^1^H NMR Spectra of Each Diastereomer

**Figure 3:**
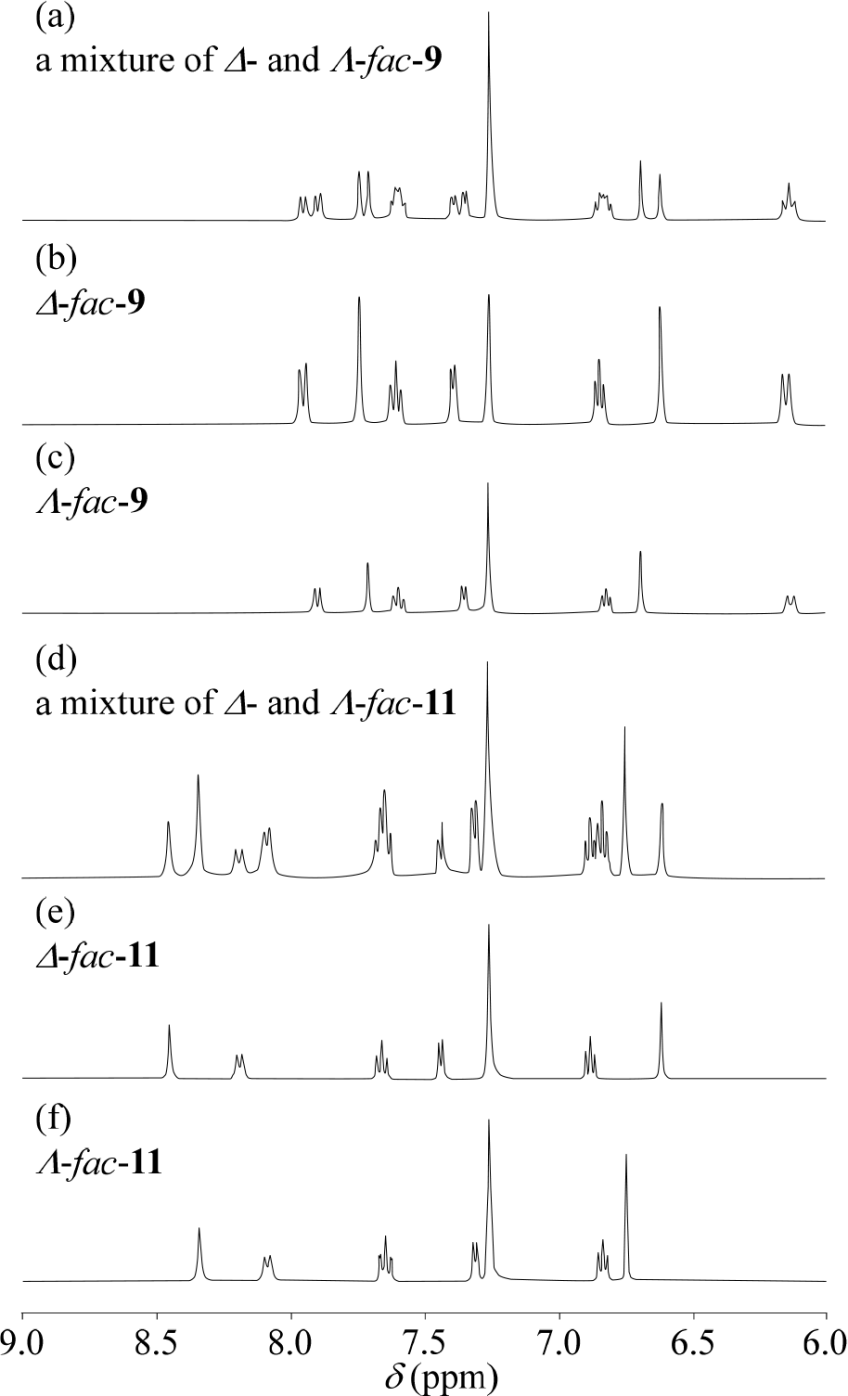
^1^H NMR spectra (400 MHz in CDCl_3_) of a diastereomeric mixture (a), Δ*-*form (b), and Λ*-*form (c) of *fac*-**9** and a diastereomeric mixture (d), Δ*-*form (e), and Λ*-*form (f) of *fac*-**11** (aromatic region).

^1^H NMR spectra (aromatic region) of each diastereomer of *fac*-**9** and *fac*-**11** in CDCl_3_ are shown in Figure 3. The chemical shifts of the aromatic protons are slightly different between those of Δ-*fac-***9** and Λ-*fac-***9**. In addition, a larger difference in chemical shift was observed for Δ-*fac-***11** and Λ-*fac-***11** than for Δ-*fac-***9** and Λ-*fac-***9**. It is likely that these phenomena are due to different interactions between the optically pure Ir(III) complex core and the chiral side chains, as was observed in their X-ray crystal structures (Figure 1) and DFT calculations (Figure S1 in the Supporting Information).^29^ A significant upfield or downfield shift of aliphatic proton signals of (1*R,*2*R*)-1,2-DAH and (1*R,*2*R*)-2-ACH units in the corresponding diastereomers (Δ*-fac*-**9** vs Λ-*fac*-**9** and Δ-*fac*-**11** vs Λ-*fac*-**11)** was not observed.

### Hydrolysis Reaction of Diastereomeric Esters

The hydrolysis of *fac*-**10**, -**11**, and -**14** was conducted using NaOH in MeOH/H_2_O (4/1) (Chart 2), and the resulting products were analyzed by HPLC using CHIRALCEL OJ-H. Only a single peak was observed in the cases of Δ- and Λ*-fac*-**4**, -**6**, and -**13** after the hydrolysis, respectively, as presented in Figure S4 in the Supporting Information, indicating that the purity of the Ir(III) complex was >99% ee and that negligible isomerization occurred during the hydrolysis.

### Photophysical Properties of Each Diastereomer and Enantiomer of Ir(III) Complexes

**Figure 4:**
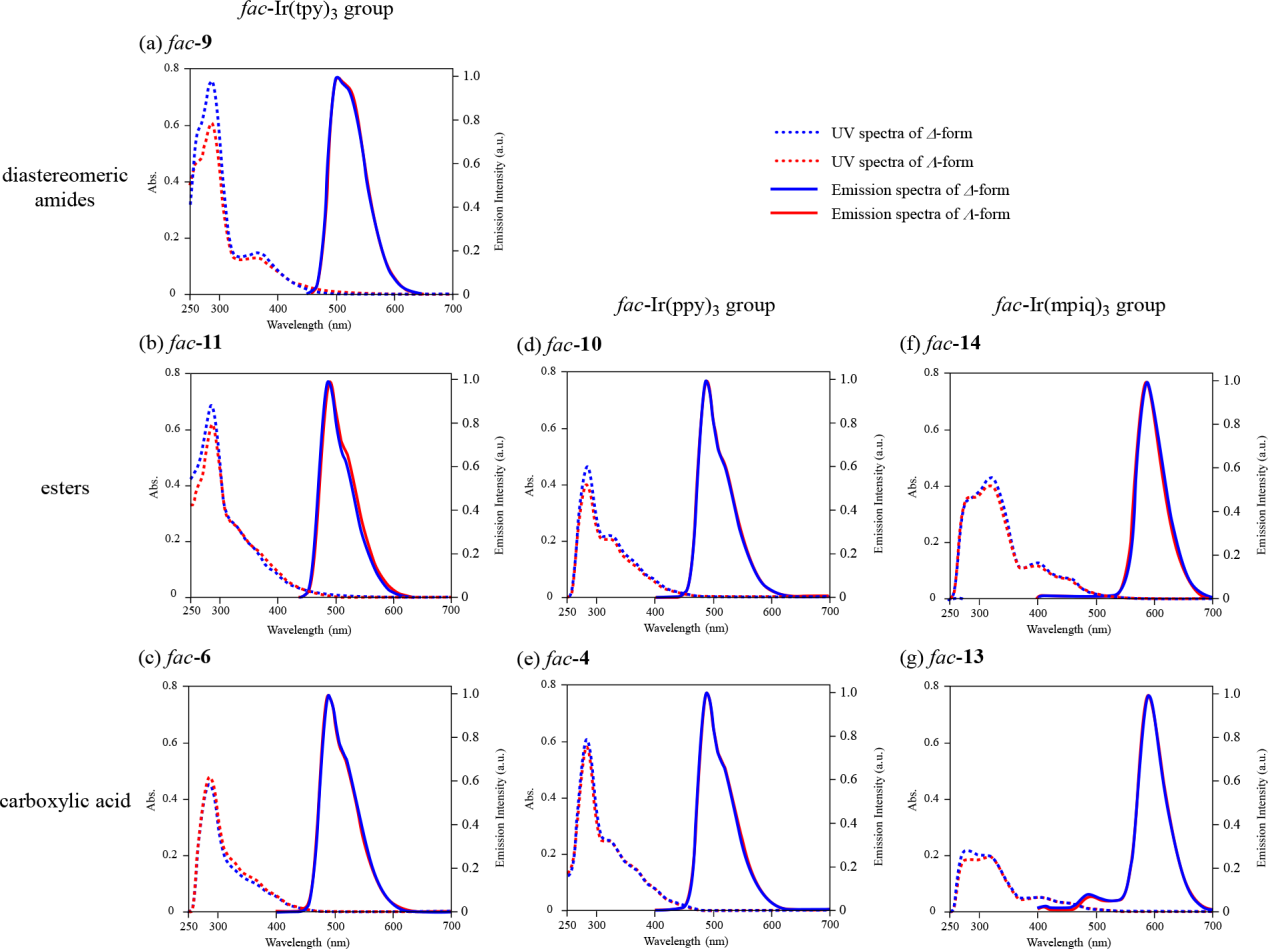
UV/vis absorption spectra and emission spectra of all diastereomers of (a) *fac*-**9**, (b) *fac*-**11**, (c) *fac*-**6**, (d) *fac*-**10**, (e) *fac*-**4**, (f) *fac*-**14**, and (g) *fac*-**13** (10 μM) in degassed DMSO at 298 K (excitation at 366 nm, au = arbitrary units). Blue dashed curves: UV/vis absorption spectra of Δ*-*forms. Red dashed curves: UV/vis absorption spectra of Λ-forms. Blue plain curves: emission spectra of Δ*-*forms. Red plain curves: emission spectra of Λ-forms.

UV/vis absorption and luminescent spectra of all diastereomers of *fac*-**4**, *fac*-**6**, *fac-***9**, *fac*-**10**, *fac*-**11**, *fac*-**13**, and *fac-***14** in degassed DMSO at 25 °C are shown in Figure 4, and their photophysical data are summarized in Table 3. The UV/vis absorption at ca. 280 nm of *fac*-**6**, *fac*-**9**, *fac*-**11**, and *fac*-**14** was assigned to the ^1^π–π* transition of the tpy ligands, and that at ca. 360 nm was assigned to a spin-allowed singlet-to-singlet metal-to-ligand charge transfer (^1^MLCT) transition, a spin-forbidden singlet-to-triplet (^3^MLCT) transition, and ^3^π–π* transitions, as were those of the corresponding racemic compounds. In the emission spectra of *fac*-**9** and *fac*-**11**, a green emission was observed with an emission maximum at ca. 490–500 nm. Their quantum yields were determined based on the Φ value of *fac*-**5** in CH_2_Cl_2_ (Φ = 0.5), which was used as a standard reference.^1d^ Both diastereomers of *fac*-**9** and *fac*-**11** were found to exhibit nearly the same UV/vis absorption and emission spectra in DMSO. Typical photophysical parameters of the carboxylates, Δ- and Λ*-*forms of *fac*-**4**, *fac*-**6**, and *fac*-**13**, are given in Table S3 of the Supporting Information.

**Table 3:** Photophysical Properties of *fac*-**9**, *fac*-**10**, *fac*-**11**, and *fac*-**14** in Degassed DMSO at 298 K ([compound] = 10 μM)

compound	λ_abs_ (nm) (ε (M^–1^ cm^–1^))	λ_em_ (nm)^a^	Φ^a^	τ (μs)
Δ-*fac-***9**	287 (7.6 × 10^4^), 366 (1.5 × 10^4^)	502	0.39^b^	1.37^d^
Λ-*fac-***9**	287 (7.6 × 10^4^), 362 (1.3 × 10^4^)	502	0.40^b^	1.39^d^
Δ*-fac*-**10**	283 (4.6 × 10^4^), 322 (2.2 × 10^4^)	488	0.60^b^	1.23^d^
Λ*-fac*-**10**	284 (4.0 × 10^4^), 322 (2.1 × 10^4^)	488	0.62^b^	1.21^d^
Δ-*fac*-**11**	285 (6.9 × 10^4^)	488	0.43^b^	1.27^d^
Λ-*fac*-**11**	287 (6.2 × 10^4^)	490	0.40^b^	1.29^d^
Δ-*fac*-**14**	319 (4.3 × 10^4^), 397 (1.3 × 10^4^)	587	0.39^c^	2.45^e^
Λ-*fac*-**14**	317 (4.0 × 10^4^), 395 (1.2 × 10^4^)	589	0.29^c^	2.32^e^

a Excitation at 366 nm.

b Quinine sulfate in 0.1 M H_2_SO_4_ (Φ = 0.55) was used as a reference.

c *fac*-Ir(mpiq)_3_ in toluene (Φ = 0.26) was used as a reference.

d A 475 nm long wave pass filter was used.

e A 550 nm long wave pass filter was used.

### Circular Dichroism (CD) Spectra of Each Diastereomer and Enantiomer of Ir(III) Complexes

**Figure 5:**
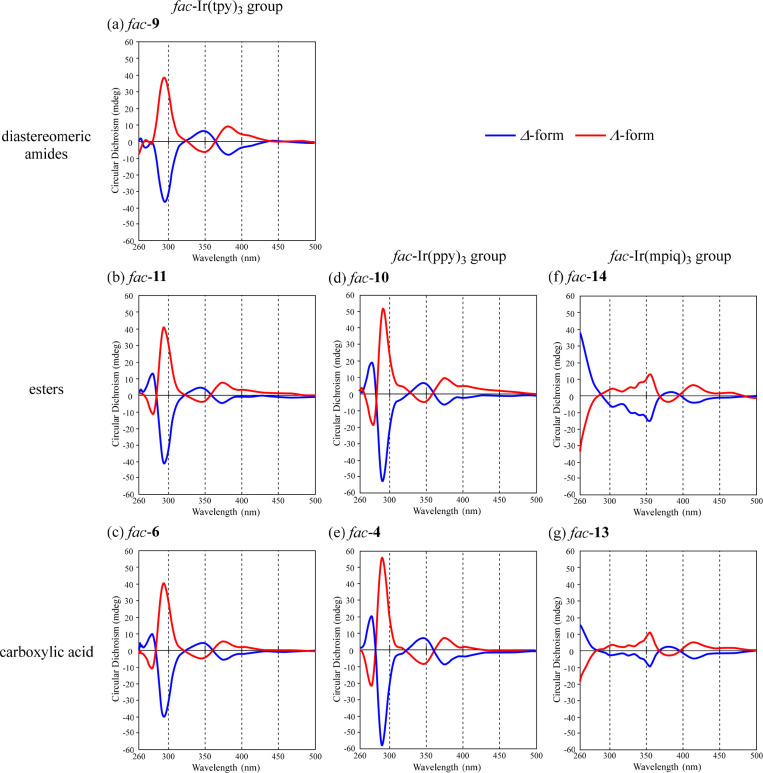
CD spectra of (a) *fac*-**9**, (b) *fac*-**11**, (c) *fac*-**6**, (d) *fac*-**10**, (e) *fac*-**4**, (f) *fac*-**14**, and (g) *fac*-**13** (10 μM) in DMSO at 298 K. Blue curves: Δ*-*forms. Red curves: Λ-forms.

**Figure 6:**
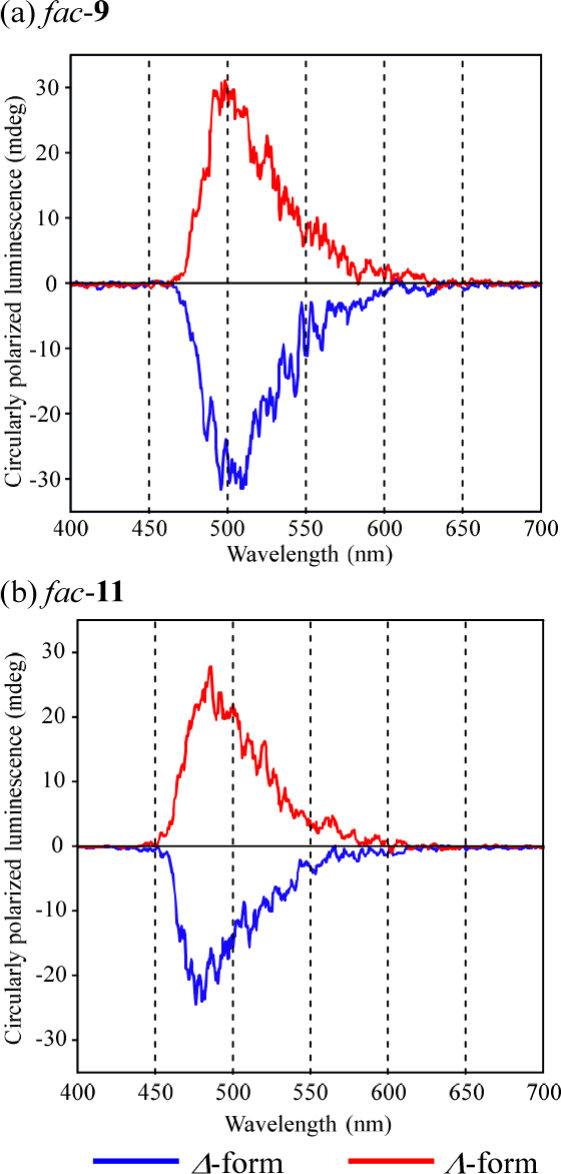
CPL spectra of (a) *fac*-**9** and (b) *fac*-**11** (100 μM) in DMSO (excitation at 288 nm for *fac*-**9** and at 286 nm for *fac*-**11**) at 298 K. Blue curves: Δ*-*forms. Red curves: Λ-forms.

CD spectra of *fac*-**4**, -**6**, -**9**, -**10**, -**11**, -**13**, and **14** in DMSO at 25 °C are shown in Figure 5, in which symmetrical spectra were obtained for all compounds. Positive and negative Cotton effects were observed at ca. 300 nm for the Λ- and Δ-forms, respectively, in the CD spectra of *fac*-**4**, -**6**, -**9**, -**10**, and -**11**, and positive and negative Cotton effects were observed at ca. 350 nm in CD spectra of Λ- and Δ-*fac*-**13** and -**14**, respectively. These results are similar to the previously reported CD spectrum of optically active *fac*-**1** that had been separated using chiral columns.^18^ It should be noted that the CD and CPL spectra (described in the next section) of two diastereomers of *fac*-**9** and *fac*-**11** were observed as mirror images (Figures 5 and 6), although Δ- and Λ-forms of **9** and **11** are diastereomers, respectively, suggesting a weak effect of chiral auxiliaries such as (*R*,*R*)-1,2-DAH and (*R*,*R*)-1-ACH on their CD and CPL spectra.

### Circularly Polarized Luminescence (CPL) Spectra of *fac*-**9** and *fac*-**11**

Circularly polarized luminescence (CPL) spectra of Δ- and Λ*-*forms of *fac*-**9** (excitation at 288 nm) and **11** (excitation at 286 nm) are shown in Figure 6. Positive and negative CPL signals were observed at ca. 503 nm for Λ*-* and Δ-*fac*-**9** (*g*_CPL_ = 2.0 × 10^–3^ and −2.0 × 10^–3^). Similarly, positive and negative CPL signals were observed at ca. 485 nm for Λ*-* and Δ-*fac*-**11** (*g*_CPL_ = 1.7 × 10^–3^ and −1.4 × 10^–3^). The CPL signals and *g*_CPL_ values for various Ir(III) complexes have been reported, and it is known that the CPL signal varies depending on the structure of the ligand.^17,21,30^ Zuo’s group reported the *g*_CPL_ values of some Ir(III) complexes (*g*_CPL_ = 3.15 × 10^–3^ for Δ-*fac*-**1**, −3.29 × 10^–3^ for Λ*-fac*-**1**, 0.915 × 10^–3^ for Δ-*fac*-**2**, −1.33 × 10^–3^ for Λ*-fac*-**2**, 1.22 × 10^–3^ for Δ-*fac*-**3**, −1.66 × 10^–3^ for Λ*-fac*-**3**).^21^ Bernhard’s group reported that the *g*_CPL_ values of Λ-*fac*-**2** is 0.78 × 10^–3^ at 525 nm (excitation at 400 nm),^17^ implying that the order (digits) of these *g*_CPL_ values are similar to those of the *g*_CPL_ values for Λ*-* and Δ-forms of *fac*-**9** and **11**. In addition, the Δ-forms of *fac*-**9** and -**11** exhibit negative signals and the Λ-forms exhibit positive signals, respectively, which are similar to the spectral data reported by Bernhard’s group.^17^ The CPL spectra of the Δ- and Λ-forms of *fac*-**4** and *fac*-**6** are shown in Figure S5 in the Supporting Information.

### Stability of Each Diastereomer against Light and Heat and in the Presence of Silica Gel

**Chart 4:**
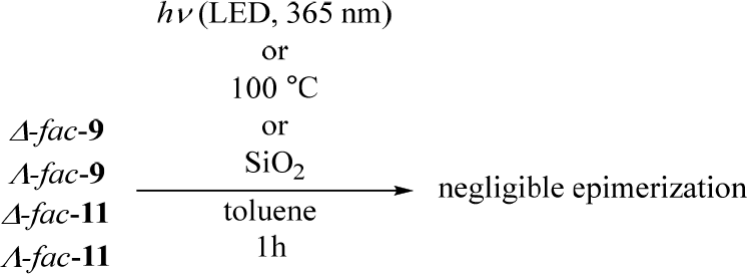
Stability Evaluation of *fac*-**9** and *fac*-**11** upon Photoirradiation, Heating, and Treatment with Silica Gel

The stabilities of the separated diastereomers (*fac*-**9** and **11**) to heat, light, and silica gel were evaluated (Chart 4). Solutions of each of the Ir(III) complexes (1 mM) in toluene^31^ were subjected to the given reaction conditions and then analyzed using normal-phase HPLC (SenshuPak PEGASIL Silica SP100, eluent CHCl_3_/MeCN = 1/2 for *fac*-**9**, hexanes/CHCl_3_ = 1/5 for *fac*-**11**, flow rate 1.0 mL/min, wavelength 254 nm). To evaluate the photostability of Δ- and Λ-*fac*-**9** and -*fac*-**11**, each solution in toluene was irradiated with light at a wavelength of 365 nm by using Twinlight (RELYON, Japan) at room temperature. Negligible decomposition and racemization were observed after the photoirradiation of these complexes for 1 h, while irradiation for a longer time induced their decomposition to some extent (observed on their emission spectra) rather than epimerization.^28^ The stability of *fac*-**9** and -**11** in toluene at 100 °C was confirmed by heating solutions of each Ir(III) complex for 3 h. In addition, negligible epimerization of Δ- and Λ-forms of *fac*-**9** and -**11** was observed when these complexes were treated with silica gel in toluene at room temperature for 1–3 h, implying negligible epimerization during the purification with silica gel chromatography and on HPLC.

## Conclusion

In summary, we report on the successful optical resolution of carboxylate derivatives of cyclometalated Ir(III) complexes via diastereomers that were formed by conjugation with chiral auxiliaries such as (1*R*,2*R*)-1,2-diaminocyclohexane ((1*R,*2*R*)-1,2-DAH) and (1*R*,2*R*)-2-aminocyclohexanol ((1*R,*2*R*)-2-ACH) groups. The racemic Ir(III) complexes *fac*-**4**, -**6**, and -**13** were converted to the corresponding diastereomers by condensation of (1*R,*2*R*)-1,2-DAH and (1*R,*2*R*)-2-ACH and successfully separated by silica gel column chromatography or normal-phase and nonchiral HPLC, not by the use of chiral HPLC columns. The absolute configurations of all of the Ir(III) complexes were determined from an X-ray single crystal structure analysis and CD spectra of *fac*-**9**, -**10**, -**11**, and -**14**. It should be noted that both diastereomers (both enantiomers with respect to the Ir(III) complex core) were obtained on a large scale. In addition, spectroscopic measurements of the synthesized diastereomeric Ir(III) complexes were also performed. Moreover, the hydrolysis of the diastereomeric esters was carried out and the resulting carboxylic acid derivative was found to be optically pure by an analysis using chiral HPLC columns. The synthesis and evaluation of anticancer activity of stereochemically pure Ir(III) complex-peptide hybrids are now in progress in our research group.

It is well-known that tris-homoleptic cyclometalated Ir(III) complexes such as Ir(tpy)_3_ complexes adopt facial (*fac*) forms and meridional (*mer*) forms and that *fac*-forms are generally more stable than *mer*-forms.^1c,1d^ In this study, we have focused on the optical resolution of the carboxylates of *fac*-Ir(tpy)_3_, *fac*-Ir(ppy)_3_, and *fac*-Ir(mpiq)_3_, which are readily accessible and important intermediates for many purposes as described in our previous publications, to demonstrate our methods.^7−11^ Our next work will be the optical resolution of *mer-*Ir complexes and the related derivatives. The results presented herein provide useful information for the future design and synthesis of optically active Ir(III) complexes and their applications as biological reagents, medicinal chemistry, photocatalysts, and related fields.^5,32^

## Experimental Procudures

### General Information

All reagents and solvents were of the highest commercial quality and were used without further purification unless otherwise noted. Anhydrous *N,N*-dimethylformamide (DMF), *N*-methyl-pyrrolidone (NMP), and CH_2_Cl_2_ were obtained by distillation from calcium hydride. Anhydrous MeCN was obtained by distillation from phosphorus(V) oxide. Anhydrous THF was obtained by distillation from sodium and benzophenone. All aqueous solutions were prepared with deionized water. IrCl_3_·3H_2_O was purchased from KANTO CHEMICAL Co. Melting points were measured on a Yanaco micro melting point apparatus. ^1^H (300 and 400 MHz) and ^13^C (100 and 150 MHz) NMR spectra were recorded on a JEOL Always 300 (JEOL, Tokyo, Japan), a JNM-ECZ400S (JEOL), and Bruker AVANCE600 spectrometers. Tetramethylsilane (TMS) was used as an internal reference for the ^1^H NMR and ^13^C NMR spectroscopy measurements of samples in CDCl_3_, DMSO-*d*_6_, and CD_3_OD. IR spectra were recorded on a PerkinElmer FTIR Spectrum 100 (ATR) instrument (PerkinElmer, Massachusetts, USA). Electrospray ionization (ESI) mass spectra were recorded on a Sciex X500R QTOF (AB SCIEX, Framingham, Massachusetts, USA) and Varian 910-MS (Varian Medical Systems, California, USA) spectrometers. Elemental analyses were performed on a PerkinElmer CHN 2400 analyzer (PerkinElmer). Optical rotations were determined with a JASCO P-1030 digital polarimeter (JASCO, Tokyo, Japan) in 50 mm cells using the D line of sodium (589 nm). Thin-layer (TLC) and silica gel column chromatographies were performed using a Merck Art. 5554 (silica gel) TLC plate and Fuji Silysia Chemical FL-100D, respectively. Commercially available DMSO and CH_2_Cl_2_ (spectrophotometric grade, FUJIFILM WAKO PURE CHEMICAL Co.) were used for the measurement of photophysical data. Photoisomerization experiments were performed with a Twin LED Light (RELYON, Tokyo, Japan) equipped with 365 nm light sources. UV/vis spectra were recorded on a JASCO V-630 spectrometer, and emission spectra were recorded on a JASCO FP-8300 spectrofluorometer. CD spectra were recorded on a Chirascan (Applied Photophysics) spectrophotometer, and CPL spectra were obtained using a JASCO CPL-300 spectrofluoropolarimeter. Gel permeation chromatography (GPC) experiments were carried out using a system (LabACE LC-5060) equipped with a UV detector (Japan Analytical Industry Co., Ltd.) and a gel permeation column (JAIGEL-2HR). Normal-phase HPLC experiments were carried out using a system consisting of a PU-980 intelligent HPLC pump (JASCO, Japan), a UV-970 intelligent UV–visible detector (JASCO), a Rheodine injector (Model No. 7125), and a Chromatopac C-R6A (Shimadzu, Japan). For analytical HPLC, a Senshupak Pegasil silica SP100 column (Senshu scientific Co., Ltd.) (4.6ϕ × 250 mm, No. 2103193S) and a CHIRALCEL OJ-H column (DAICEL CHEMICAL INDUSTRIES, Ltd.) (4.6ϕ × 250 mm, No. OJH0CE-OL015) were used. For preparative HPLC, a Senshu Pak Pegasil Silica SP100 column (Senshu scientific Co., Ltd.) (10ϕ × 250 mm, No. 2104062S) was used.

### Synthesis.^29^

#### Δ*-fac-***9** and Λ*-fac-***9**

PyBOP (176 mg, 0.338 mmol) was added to a solution of racemic *fac*-**6**^8a^ (82 mg, 0.098 mmol) and DIEA (101 μL, 0.580 mmol) in DMF (2 mL), and the reaction mixture was stirred at room temperature for 2 h. To the reaction mixture was added a mono-Boc-protected (1*R,*2*R*)-1,2-diaminocyclohexane ((*R*,*R*)-**7**)^23^ (69 mg, 0.324 mmol), and the reaction mixture was stirred at room temperature for 20 h. The solvent was removed under reduced pressure. The resulting residue was purified by silica gel column chromatography (CHCl_3_/MeOH = 10:1) and GPC (CHCl_3_) to afford a diastereomeric mixture of *fac*-**9** as a yellow solid (154 mg, quant.). *Fac*-**9** was separated by normal-phase HPLC (CHCl_3_/MeCN = 1/2, flow rate 3.0 mL/min, *t*_r_ (retention time) = 13.2 min (Δ-*fac*-**9**), 20.7 min (Λ-*fac*-**9**)), and the solvent was removed under reduced pressure to afford Δ-*fac*-**9** (46 mg, 33% yield) and Λ-*fac*-**9** (51 mg, 36% yield) as yellow solids, respectively. Δ-*fac*-**9**: mp >300 °C. IR (ATR): ν 3319, 2930, 2858, 1690, 1635, 1600, 1505, 1472, 1425, 1389, 1365, 1317, 1255, 1162, 1069, 1012, 908, 869, 778, 750, 521, 454, 441, 429, 420. 410 cm^–1^. ^1^H NMR (400 MHz, CDCl_3_/TMS): δ 7.96 (d, *J* = 8.4 Hz, 3H), 7.74 (s, 3H), 7.61 (dt, *J* = 7.8, 1.6 Hz, 3H), 7.39 (d, *J* = 5.2 Hz, 3H), 6.85 (t, *J* = 6.0 Hz, 3H), 6.62 (s, 3H), 6.15 (d, *J* = 8.8 Hz, 3H), 4.96 (d, 8.4 Hz, 3H), 3.88–3.83 (m, 3H), 3.45–3.40 (m, 3H), 2.25 (s, 9H), 2.13–2.05 (m, 6H), 1.77 (m, 6H), 1.39–1.26 (m, 39H) ppm. ^13^C NMR (100 MHz, CDCl_3_/TMS): δ 171.4, 165.8, 164.8, 156.6, 146.9, 141.4, 139.7, 137.8, 136.3, 127.6, 122.7, 121.9, 119.0, 79.5, 54.9, 53.7, 33.1, 32.8, 28.4, 25.0, 24.9, 20.7 ppm. ESI-MS (*m*/*z*): calcd for C_72_H_91_N_9_O_9_^191^Ir [M + H]^+^ 1416.6540; found 1416.6540. Anal. Calcd for C_72_H_90_N_9_O_9_Ir·0.8hexane·0.5CHCl_3_·1.5MeCN: C, 59.43; H, 6.59; N, 9.05%. Found: C, 59.46; H, 6.52; N, 9.12%. Λ-*fac*-**9**: mp >300 °C. IR (ATR): ν 3318, 2929, 2857, 1691, 1634, 1600, 1505, 1471, 1425, 1389, 1364, 1318, 1255, 1161, 1068, 1012, 933, 908, 779, 749, 515, 464, 440, 422, 416 cm^–1^. ^1^H NMR (400 MHz, CDCl_3_/TMS): δ 7.90 (d, *J* = 8.4 Hz, 3H), 7.71 (s, 3H), 7.60 (t, *J* = 7.6 Hz, 3H), 7.35 (d, *J* = 5.6 Hz, 3H), 6.82 (t, *J* = 6.4 Hz, 3H), 6.69 (s, 3H), 6.13 (d, *J* = 8.4 Hz, 3H), 4.98 (d, *J* = 8.8 Hz, 3H), 3.87–3.86 (m, 3H), 3.42–3.40 (m, 3H), 2.25 (s, 9H), 2.17–2.14 (m, 3H), 2.08–2.06 (m, 3H), 1.78–1.76 (m, 6H), 1.39–1.27 (m, 39H) ppm. ^13^C NMR (100 MHz, CDCl_3_/TMS): δ 171.7, 165.7, 164.6, 156.5, 146.9, 141.5, 139.5, 137.2, 136.2, 128.0, 122.8, 121.8, 118.9, 79.3, 54.9, 53.8, 33.1, 32.8, 28.3, 25.0, 24.8, 20.6 ppm. ESI-MS (*m*/*z*): calcd for C_72_H_90_N_9_O_9_^191^Ir [M + H]^+^ 1416.6540; found 1416.6545. Anal. Calcd for C_72_H_90_N_9_O_9_Ir·0.8hexane·0.5CHCl_3_·1.5MeCN: C, 59.43; H, 6.59; N, 9.05%. Found: C, 59.47; H, 6.48; N, 9.25%.

#### Δ*-fac-***10** and Λ*-fac-***10**

FTFBA (55 mg, 0.139 mmol) was added to a solution of racemic *fac*-**4**^8a^ (30 mg, 0.037 mmol) and DIEA (47 μL, 0.267 mmol) in NMP (130 μL), and the reaction mixture was stirred at room temperature for 2.5 h. To the reaction mixture were added Boc-protected (1*R*,2*R*)-2-aminocyclohexanol ((*R*,*R*)-**8**)^24^ (62 mg, 0.277 mmol) and DMAP (2.4 mg, 0.020 mmol), and the reaction mixture was stirred at 80 °C for 3 h. The reaction mixture was diluted with CHCl_3_ and washed with a saturated aqueous solution of NH_4_Cl and brine. The organic layer was dried over Na_2_SO_4_, filtered, and concentrated under reduced pressure. The resulting residue was purified by silica gel column chromatography (hexanes/CHCl_3_ = 1:1 to 1:2) and GPC (CHCl_3_) to afford a diastereomeric mixture of *fac*-**10** as a yellow solid (24 mg, 47% yield). *fac*-**10** was separated by preparative normal-phase HPLC (CHCl_3_ only, *t*_r_ = 21.5 min (Δ-*fac-***10**), 23.5 min (Λ-*fac-***10**), 3.0 mL/min), and solvent was removed under reduced pressure to afford Δ-*fac-***10** (10 mg, 20% yield) and Λ-*fac-***10** (12 mg, 23% yield) as yellow solids, respectively. Δ-*fac*-**10**: mp 248 °C. IR (ATR): ν 3311, 2928, 2857, 1699, 1677, 1588, 1531, 1476, 1452, 1426, 1364, 1320, 1302, 1243, 1158, 1111, 1063, 1028, 998, 913, 865, 842, 787, 762, 724, 641, 494, 418 cm^–1^. ^1^H NMR (400 MHz, CDCl_3_/TMS): δ 8.41 (s, 3H), 8.15 (d, *J* = 8.4 Hz, 3H), 7.69 (dt, *J* = 7.8, 1.6 Hz, 3H), 7.51 (d, *J* = 4.8 Hz, 3H), 7.40 (dd, *J* = 7.8, 1.4 Hz, 3H), 6.95 (dt, *J* = 6.6, 1.2 Hz, 3H), 6.81 (d, *J* = 8.0 Hz, 3H), 4.77–4.73 (m, 3H), 4.55 (d, *J* = 9.2 Hz, 3H), 3.75–3.73 (m, 3H), 2.09 (d, *J* = 12.4 Hz, 6H), 1.76–1.73 (m, 6H), 1.53–1.26 (m, 21H), 1.19, (s, 27H) ppm. ^13^C NMR (100 MHz, CDCl_3_/TMS): δ 170.2, 167.9, 165.5, 155.6, 146.9, 144.1, 136.9, 136.8, 130.5, 125.4, 122.6, 122.3, 119.8, 79.2, 75.6, 53.5, 32.3, 31.2, 28.2, 24.6, 24.1 ppm. ESI-MS (*m*/*z*): calcd for C_69_H_85_N_7_O_12_^191^Ir [M + NH_4_]^+^ 1394.5857; found 1394.5875. Anal. Calcd for C_69_H_81_N_6_O_12_Ir·0.86hexane·0.29CHCl_3_: C, 60.12; H, 6.32; N, 5.65%. Found: C, 60.40; H, 6.02; N, 5.35%. Λ-*fac*-**10**: mp 207 °C. IR (ATR): ν 3355, 2929, 2859, 1687, 1587, 1561, 1504, 1476, 1452, 1412, 1390, 1364, 1321, 1302, 1234, 1160, 1111, 1061, 1028, 998, 953, 911, 867, 844, 787, 759, 724, 665, 641, 493, 432, 417 cm^–1^. ^1^H NMR (400 MHz, CDCl_3_/TMS): δ 8.37 (s, 3H), 8.11 (d, *J* = 8.4 Hz, 3H), 7.69 (dt, *J* = 8.0, 1.6 Hz, 3H), 7.46–7.42 (m, 6H), 6.92 (t, *J* = 6.0 Hz, 3H), 6.89 (d, *J* = 7.6 Hz, 3H), 4.75–4.73 (m, 3H), 4.55 (d, *J* = 9.6 Hz, 3H), 3.73–3.72 (m, 3H), 2.10 (d, *J* = 12.4 Hz, 6H), 1.77–1.72 (m, 6H), 1.53–1.48 (m, 6H), 1.36 (t, *J* = 10.4 Hz, 6H), 1.23 (s, 27H) ppm. ^13^C NMR (100 MHz, CDCl_3_/TMS): δ 170.2, 168.0, 165.5, 155.5, 146.8, 144.0, 137.0, 136.8, 130.7, 125.3, 122.5, 122.4, 119.7, 79.0, 75.4, 53.6, 32.4, 31.2, 28.2, 24.6, 24.1 ppm. ESI-MS (*m*/*z*): calcd for C_69_H_85_N_7_O_12_^191^Ir [M + NH_4_]^+^ 1394.5857; found 1394.5862.

#### Δ*-fac-***11** and Λ*-fac-***11**

MNBA (374 mg, 1.087 mmol) was added to a solution of racemic *fac*-**6** (249 mg, 0.300 mmol) and DIEA (368 μL, 2.113 mmol) in NMP (1 mL), and the reaction mixture was stirred at room temperature for 4 h. To the reaction mixture were added Boc-protected (1*R,*2*R*)-2-aminocyclohexanol ((*R*,*R*)-**8**) (472 mg, 2.122 mmol) and DMAP (11 mg, 0.090 mmol), and the reaction mixture was stirred at 80 °C for 2 h. The reaction mixture was diluted with CHCl_3_ and washed with a saturated aqueous solution of NH_4_Cl and brine. The organic layer was dried over Na_2_SO_4_, filtered, and concentrated under reduced pressure. The resulting residue was purified by silica gel column chromatography (hexanes/CHCl_3_ = 1/0 to 1/1) to afford Δ-*fac*-**11** (324 mg, a mixture of Δ-*fac*-**11** and (*R*,*R*)-**8**) and Λ-*fac*-**11** (150 mg, 35% yield) as yellow solids, respectively. The mixture of Δ-*fac*-**11** and (*R*,*R*)-**14** was reprecipitated from hexanes/CHCl_3_ to afford Δ-*fac*-**11** (161 mg, 38% yield) as a yellow solid. Δ-*fac*-**11**: mp 220 °C (dec.). IR (ATR): ν 3313, 2929, 2858, 1698, 1677, 1584, 1526, 1473, 1452, 1424, 1380, 1364, 1318, 1294, 1271, 1229, 1197, 1157, 1087, 998, 910, 883, 867, 851, 777, 754, 723, 699, 658, 630, 524, 501, 489, 441, 425, 406 cm^–1^. ^1^H NMR (400 MHz, CDCl_3_/TMS): δ 8.46 (s, 3H), 8.19 (d, *J* = 8.0 Hz, 3H), 7.66 (td, *J* = 7.8, 1.2 Hz, 3H), 7.44 (d, *J* = 5.19, 3H), 6.88 (t, *J* = 6.2 Hz, 3H), 6.62 (s, 3H), 4.73 (td, *J* = 10.2, 3.7 Hz, 3H), 4.53 (d, *J* = 9.6 Hz, 3H), 3.82–3.80 (m, 3H), 2.36 (s, 9H), 2.09 (m, 6H), 1.77 (m, 6H), 1.39–1.27 (m, 12H), 1.18 (s, 27H) ppm. ^13^C NMR (100 MHz, CDCl_3_/TMS): δ 169.3, 168.1, 165.6, 155.8, 146.7, 142.3, 141.8, 140.1, 136.5, 127.1, 122.2, 120.7, 119.8, 79.3, 75.3, 53.4, 32.6, 31.4, 28.3, 24.8, 24.2, 22.4 ppm. ESI-MS (*m*/*z*): calcd for C_72_H_91_N_7_O_12_^191^Ir [M + NH_4_]^+^ 1436.6326; found 1436.6329. Anal. Calcd for C_72_H_87_N_6_O_12_Ir·0.2CHCl_3_·MeCN: C, 59.99; H, 6.12; N, 6.60%. Found: C, 59.80; H, 6.08; N, 6.53%. Λ-*fac*-**11**: mp 195 °C (dec). IR (ATR): ν 3356, 2932, 2860, 1703, 1584, 1561, 1510, 1473, 1451, 1390, 1365, 1318, 1293, 1230, 1195, 1168, 1082, 997, 911, 850, 777, 749, 722, 523, 494, 458, 443, 433 cm^–1^. ^1^H NMR (400 MHz, CDCl_3_/TMS): δ 8.34 (s, 3H), 8.09 (d, *J* = 8.4 Hz, 3H), 7.65 (td, *J* = 7.8, 1.6 Hz, 3H), 7.31 (d, *J* = 5.6 Hz, 3H), 6.84 (t, *J* = 6.4 Hz, 3H), 6.75 (s, 3H), 4.78 (td, *J* = 10.6, 3.9 Hz, 3H), 4.60 (d, *J* = 8.4 Hz, 3H), 3.75–3.73 (m, 3H), 2.39 (s, 9H), 2.13 (t, *J* = 16.0 Hz, 6H), 1.80–1.70 (m, 6H), 1.42–1.27 (m, 12H), 1.20 (s, 27H) ppm. ^13^C NMR (100 MHz, CDCl_3_/TMS): δ 169.0, 168.6, 165.6, 155.6, 146.6, 142.1, 141.8, 140.3, 136.5, 126.7, 121.9, 121.1, 120.0, 78.9, 74.8, 53.8, 32.7, 31.5, 28.3, 24.6, 24.2, 22.4 ppm. ESI-MS (*m*/*z*): calcd for C_72_H_87_N_6_O_12_^191^Ir [M]^+^ 1418.5982; found 1418.5995. Anal. Calcd for C_72_H_87_N_6_O_12_Ir·0.2CHCl_3_: C, 60.03; H, 6.08; N, 5.82%. Found: C, 59.80; H, 6.15; N, 6.08%.

#### Racemic *fac*-**13**

Phosphorus oxychloride (830 μL) was added dropwise to DMF (8.3 mL), and the resulting mixture was stirred at 0 °C for 1 h, to which *fac*-**12**^8d^ (245 mg, 0.289 mmol) was then added to produce a yellow solution. After stirring at 80 °C for 21 h, the deep red reaction mixture was allowed to cool at 0 °C, and the pH was adjusted to 11 by adding 3 N aqueous NaOH. The red solid was isolated by filtration and washed with H_2_O. The resulting residue was diluted with CHCl_3_ and dried over Na_2_SO_4_, filtered, and concentrated under reduced pressure. The formylated compound (*fac*-tris[1-(5′-formyl-4’methylphenyl)isoquinoline]iridium(III) (Ir(mpiq-CHO)_3_) was obtained as an orange powder by reprecipitation from hexanes/CHCl_3_ (258 mg, 96% yield). Mp: 275 °C (dec). IR (ATR): ν 2162, 1669, 1566, 1500, 1434, 1390, 1348, 1281, 1216, 1145, 1088, 1018, 927, 814, 739, 722, 679, 656, 631, 578, 558, 518, 460, 450, 421 cm^–1^. ^1^H NMR (400 MHz, CDCl_3_/TMS): δ 10.20 (s, 3H), 8.98 (d, *J* = 8.0 Hz, 3H), 8.62 (s, 3H), 7.81–7.71 (m, 9H), 7.32 (d, *J* = 6.0 Hz, 3H), 7.23 (d, *J* = 6.4 Hz, 3H), 6.85 (s, 3H), 2.45 (s, 9H) ppm. ^13^C NMR (100 MHz, CDCl_3_/TMS): δ 192.1, 176.3, 165.8, 144.3, 141.9, 140.1, 139.2, 136.8, 132.2, 130.9, 128.6, 128.0, 127.3, 127.1, 126.5, 121.3, 19.5 ppm. ESI-MS (*m*/*z*): calcd for C_51_H_37_N_3_O_3_^191^Ir [M + H]^+^ 930.2435; found 930.2454.

A mixture of NaClO_2_ (740 mg, 8.2 mmol) and NaH_2_PO_4_·2H_2_O (1264 mg, 8.1 mmol) in H_2_O (2.5 mL) was added dropwise to a solution of Ir(mpiq-CHO)_3_ (258 mg, 0.277 mmol) and 2-methyl-2-butene (853 μL, 8.1 mmol) in DMSO (10 mL) at room temperature. After stirring for 22 h, the pH of the reaction mixture was adjusted to 1 by adding 2 N aqueous HCl. The resulting solid was isolated on a filter and washed with H_2_O to afford *fac*-**13** as an orange solid (298 mg, quantitative yield). Mp: 297 °C (dec). IR (ATR): ν 2921, 1697, 1577, 1548, 1519, 1500, 1435, 1347, 1298, 1279, 1235, 1165, 1145, 1088, 1012, 947, 914, 816, 784, 755, 739, 697, 675, 631, 603, 558, 497, 465, 422, 415 cm^–1^. ^1^H NMR (400 MHz, DMSO-*d*_6_/TMS): δ 8.87 (d, *J* = 8.0 Hz, 3H), 8.76 (s, 3H), 7.99 (dd, *J* = 8.0, 1.2 Hz, 3H), 7.90–7.82 (m, 6H), 7.56 (d, *J* = 6.4 Hz, 3H), 7.41 (d, *J* = 4.4 Hz, 3H), 6.72 (s, 3H), 2.27 (s, 9H) ppm. ^13^C NMR (100 MHz, DMSO-*d*_6_/TMS): δ 171.0, 168.9, 164.7, 143.3, 140.2, 139.4, 139.2, 136.3, 131.5 131.0, 128.9, 127.7, 125.9, 125.5, 121.5, 121.4, 22.1 ppm. ESI-MS (*m*/*z*): calcd for C_51_H_37_N_3_O_6_^191^Ir [M + H]^+^ 978.2283; found 978.2299. Anal. Calcd for C_51_H_36_N_3_O_6_Ir·0.75CHCl_3_·4H_2_O: C, 54.49; H, 3.95; N, 3.68%. Found: C, 54.33; H, 3.77; N, 3.50%.

#### Δ*-fac-***14** and Λ*-fac-***14**

MNBA (76 mg, 0.221 mmol) was added to a solution of racemic *fac*-**13** (59 mg, 0.060 mmol) and DIEA (76 μL, 0.437 mmol) in NMP (200 μL), and the reaction mixture was stirred at room temperature for 45 min. To the reaction mixture were added (*R,R*)-**8** (97 mg, 0.434 mmol) and DMAP (1.8 mg, 0.015 mmol), and the resulting mixture was stirred at 80 °C for 2 h. The reaction mixture was diluted with CHCl_3_ and washed with a saturated aqueous solution of NH_4_Cl and brine. The organic layer was dried over Na_2_SO_4_, filtered, and concentrated under reduced pressure. The resulting residue was purified by silica gel column chromatography (hexanes/CHCl_3_ = 2:1 to 1:3) and GPC (CHCl_3_) to afford a diastereomeric mixture of *fac*-**14**. *fac*-**14** was separated by normal-phase HPLC (hexanes/CHCl_3_ = 1/4, *t*_r_ = 19.5 min (Δ-*fac-***14**), 21.0 min (Λ-*fac-***14**), 3.0 mL/min), and solvent was removed under reduced pressure to afford Δ-*fac-***14** (22 mg, 23% yield) and Λ-*fac-***14** (18 mg, 19% yield) as red solids, respectively. Δ-*fac*-**14**: mp 278 °C. IR (ATR): ν 3378, 2926, 2854, 1693, 1578, 1502, 1452, 1391, 1364, 1317, 1266, 1239, 1202, 1168, 1079, 1028, 992, 911, 869, 815, 775, 753, 741, 707, 676, 633, 604, 507, 418, 406 cm^–1^. ^1^H NMR (400 MHz, CDCl_3_/TMS): δ 9.11 (d, *J* = 8.8 Hz, 3H), 8.92 (s, 3H), 7.84 (t, *J* = 7.4 Hz, 3H), 7.76–7.67 (m, 6H), 7.43 (d, *J* = 5.6 Hz, 3H), 7.16 (d, *J* = 6.4 Hz, 3H), 6.88 (s, 3H), 4.83 (td, *J* = 10.0, 4.0 Hz, 3H), 4.66 (d, *J* = 9.2 Hz, 3H), 3.76–3.75 (m, 3H), 2.37 (s, 9H), 2.12 (d, *J* = 12.4 Hz, 3H), 2.02 (d, *J* = 12.0 Hz, 3H), 1.77–1.70 (m, 6H), 1.38–1.30 (m, 3H), 1.25 (s, 9H), 1.18 (s, 27H) ppm. ^13^C NMR (100 MHz, CDCl_3_/TMS): δ 172.7, 168.4, 166.0, 155.6, 143.6, 142.0, 140.2, 139.3, 136.7, 132.4, 130.6, 128.5, 126.9, 126.6, 120.7, 79.0, 74.4, 53.8, 32.6, 31.3, 29.7, 28.2, 24.5, 24.2, 22.4, 14.1 ppm. ESI-MS (*m*/*z*): calcd for C_84_H_97_N_7_O_12_^191^Ir [M + NH_4_]^+^ 1586.6796; found 1586.6797. Λ-*fac*-**14**: mp 152 °C. IR (ATR): ν 3361, 2926, 2857, 1697, 1578, 1502, 1451, 1391, 1364, 1348, 1318, 1238, 1203, 1164, 1078, 1027, 991, 914, 868, 814, 776, 754, 739, 709, 676, 633, 601, 451, 416 cm^–1^. ^1^H NMR (400 MHz, CDCl_3_/TMS): δ 9.03 (d, *J* = 8.4 Hz, 3H), 8.90 (s, 3H), 7.80–7.67 (m, 12H), 7.14 (d, *J* = 5.6 Hz, 3H), 6.87 (s, 3H), 4.85 (td, *J* = 10.4, 4.4 Hz, 3H), 4.67 (d, *J* = 8.4 Hz, 3H), 3.71–3.67 (m, 3H), 2.40 (s, 9H), 2.13–2.10 (m, 6H), 1.78–1.72 (m, 6H), 1.36 (t, *J* = 9.4 Hz, 3H), 1.25 (s, 9H), 1.07 (s, 27H) ppm. ^13^C NMR (100 MHz, CDCl_3_/TMS): δ 172.5, 168.6, 166.3, 155.5, 143.6, 142.0, 140.4, 139.2, 136.7, 132.5, 130.6, 128.2, 127.6, 127.0, 126.5, 120.6, 78.9, 74.2, 54.1 ppm. ESI-MS (*m*/*z*): calcd for C_69_H_85_N_7_O_12_^191^Ir [M + NH_4_]^+^ 1586.6796; found 1586.6821.

#### Δ-*fac*-**6**

A 5 N solution of NaOH in water (350 μL) was added to a solution of Δ-*fac*-**11** (70 mg, 49 μmol) in MeOH (1.4 mL), and the reaction mixture was stirred at 80 °C for 19 h. After the mixture was cooled to room temperature, the solvent was removed under reduced pressure. After saturated aqueous NaHCO_3_ was added, the solution was washed with AcOEt. To the aqueous layer was added a 2 N aqueous solution of HCl to adjust the pH to 1, and the resulting solution was extracted with AcOEt. The combined organic layer was washed with brine, dried over Na_2_SO_4_, filtered, and concentrated under reduced pressure. The resulting residue was purified by silica gel column chromatography (hexanes/CHCl_3_ = 1/0 to 0/1, AcOEt/MeOH = 1/0 to 10/1) to afford Δ-*fac*-**6** (29 mg, 71% yield, 99% ee) as a yellow solid. Mp: >300 °C. IR (ATR): ν 2926, 1672, 1581, 1560, 1522, 1472, 1419, 1294, 1239, 1157, 1069, 909, 778, 748, 679, 626, 524, 494, 429, 412 cm^–1^. ^1^H NMR (300 MHz, DMSO-*d*_6_/TMS): δ 8.24 (s, 1H), 8.17 (d, *J* = 8.4 Hz, 3H), 7.85 (t, *J* = 7.7 Hz, 3H), 7.43 (d, *J* = 4.8, 3H), 7.18 (t, *J* = 6.2, 3H), 6.58 (s, 3H), 1.36 (s, 9H) ppm. ^13^C NMR (100 MHz, DMSO-*d*_6_/TMS): δ 210.1, 189.5, 169.1, 167.8, 164.0, 146.7, 142.2, 139.7, 139.2, 123.2, 121.8, 119.2, 22.2 ppm. ESI-MS (*m*/*z*): calcd for C_39_H_31_^191^IrN_3_O_6_ [M + H]^+^ 828.1813; found 828.1828. Anal. Calcd for C_39_H_30_N_3_O_6_Ir·0.4hexane·0.1CHCl_3_: C, 56.95; H, 4.11; N, 4.80%. Found: C, 56.72; H, 4.41; N, 4.56%.

#### Λ-*fac*-**6**

Λ-*fac*-**6** was prepared by using a procedure similar to that for Δ-*fac*-**6**. A 5 N NaOH solution in water (350 μL) was added to a solution of Λ-*fac*-**11** (70 mg, 49 μmol) in MeOH (1.4 mL), and the reaction mixture was stirred at 80 °C for 5.5 h. After cooling to room temperature, the solvent was removed under reduced pressure. After addition of saturated aqueous NaHCO_3_, the solution was washed with AcOEt. To the aqueous layer was added a 2 N aqueous solution of HCl to adjust the pH to 1, and the resulting solution was extracted with AcOEt. The combined organic layer was washed with brine, dried over Na_2_SO_4_, filtered, and concentrated under reduced pressure. The resulting residue was purified by silica gel column chromatography (hexanes/CHCl_3_ = 1/0 to 0/1, AcOEt/MeOH = 1/0 to 10/1) to afford Λ-*fac*-**6** (37 mg, 91% yield, >99% ee) as a yellow solid. Mp: >300 °C. IR (ATR): ν 2924, 1667, 1580, 1521, 1471, 1411, 1298, 1239, 1156, 1069, 908, 777, 747, 669, 626, 575, 523, 498, 477, 465, 454, 435, 426, 417, 410 cm^–1^. ^1^H NMR (300 MHz, DMSO-*d*_6_/TMS): δ 8.24 (s, 3H), 8.17 (d, *J* = 8.1 Hz, 3H), 7.85 (t, *J* = 7.8 Hz, 3H), 7.43 (d, *J* = 4.8 Hz, 3H), 7.18 (t, *J* = 6.0 Hz, 3H), 6.58 (s, 3H), 1.36 (s, 9H) ppm. ^13^C NMR (150 MHz, DMSO-*d*_6_/TMS): δ 169.2, 167.8, 164.2, 146.8, 142.3, 139.8, 139.3, 137.7, 125.9, 123.3, 121.9, 119.3, 22.3 ppm. ESI-MS (*m*/*z*): calcd for C_39_H_30_^191^IrN_3_O_6_ [M]^+^ 827.1735; found 827.1735. Anal. Calcd for C_39_H_30_N_3_O_6_Ir·0.45H_2_O: C, 55.96; H, 3.72; N, 5.02%. Found: C, 56.22; H, 3.62; N, 4.76%.

#### Δ-*fac*-**4**

Δ-*fac*-**4** was prepared using a procedure similar to that for Δ-*fac*-**6**. A 5 N NaOH solution in H_2_O (100 μL) was added to a solution of Δ-*fac*-**10** (10 mg, 7.4 μmol) in MeOH (400 μL), and the reaction mixture was stirred at 80 °C for 3 h. After cooling to room temperature, the solvent was removed under reduced pressure. After the addition of saturated aqueous NaHCO_3_, the solution was washed with AcOEt. To the aqueous layer was added a 2 N aqueous solution of HCl to adjust the pH to 1, and the resulting solution was extracted with AcOEt. The combined organic layer was washed with brine, dried over Na_2_SO_4_, filtered, and concentrated under reduced pressure. The resulting residue was reprecipitated from hexanes/THF to afford Δ-*fac*-**4** (6 mg, quantitative, >99% ee) as a yellow solid. Mp: >300 °C. IR (ATR): ν 2487, 2160, 1978, 1670, 1584, 1533, 1475, 1456, 1414, 1382, 1298, 1211, 1101, 1061, 1048, 1028, 997, 844, 786, 765, 736, 669, 640, 556, 486, 463, 427, 420, 410 cm^–1^. ^1^H NMR (600 MHz, DMSO-*d*_6_/TMS): δ 12.36 (bs, 3H), 8.30 (d, *J* = 1.2 Hz, 3H), 8.28 (d, *J* = 8.4 Hz, 3H), 7.89 (td, *J* = 7.8, 1.2 Hz, 3H), 7.51 (dd, *J* = 5.4, 0.6 Hz, 3H), 7.28 (dd, *J* = 8.4, 1.8 Hz, 3H), 7.24 (td, *J* = 6.6, 1.2 Hz, 3H), 6.73 (d, *J* = 7.8 Hz, 3H) ppm. ^13^C NMR (150 MHz, DMSO-*d*_6_/TMS): δ 169.3, 169.0, 168.2, 163.9, 147.0, 144.4, 136.1, 129.8, 124.8, 123.9, 122.9, 119.7 ppm. ESI-MS (*m*/*z*): calcd for C_36_H_25_^191^IrN_3_O_6_ [M + H]^+^ 786.1344; found 786.1344.

#### Λ-*fac*-**4**

Λ-*fac*-**4** was prepared by using a procedure similar to that for Δ-*fac*-**6**. A 5 N NaOH solution in H_2_O (100 μL) was added to a solution of Λ-*fac*-**10** (10 mg, 7.3 μmol) in MeOH (400 μL), and the reaction mixture was stirred at 80 °C for 2 h. After cooling to room temperature, the solvent was removed under reduced pressure. After saturated aqueous NaHCO_3_ was added, the solution was washed with AcOEt. To the aqueous layer was added a 2 N aqueous solution of HCl to adjust the pH to 1, and the resulting solution was extracted with AcOEt. The combined organic layer was washed with brine, dried over Na_2_SO_4_, filtered, and concentrated under reduced pressure. The resulting residue was reprecipitated from hexanes/THF to afford Λ-*fac*-**4** (7 mg, quantitative, >99% ee) as a yellow solid. Mp: >300 °C. IR (ATR): ν 3051, 1673, 1586, 1475, 1415, 1212, 1061, 1029, 847, 747, 668, 641, 505, 430, 416, 405 cm^–1^. ^1^H NMR (600 MHz, DMSO-*d*_6_/TMS): δ 12.33 (bs, 3H), 8.30 (d, *J* = 1.8 Hz, 3H), 8.28 (d, *J* = 8.4 Hz,3H), 7.90 (td, *J* = 7.8, 1.2 Hz, 3H), 7.51 (dd, *J* = 5.4, 0.6 Hz, 3H), 7.28 (dd, *J* = 7.8, 1.8 Hz, 3H), 7.24 (td, *J* = 6.6, 1.2 Hz, 3H), 6.73 (d, *J* = 7.8 Hz, 3H) ppm. ^13^C NMR (150 MHz, DMSO-*d*_6_/TMS): δ 185.9, 168.3, 164.1, 147.1, 144.5, 138.0, 136.2, 136.1, 129.8, 122.9, 119.8, 106.1 ppm. ESI-MS (*m*/*z*): calcd for C_36_H_25_^191^IrN_3_O_6_ [M + H]^+^ 786.1344; found 786.1348. Anal. Calcd for C_36_H_24_N_3_O_6_Ir·0.4CHCl_3_·1.5MeOH: C, 51.58; H, 3.47; N, 4.76%. Found: C, 51.80; H, 3.29; N, 4.50%.

#### Δ-*fac*-**13**

Δ-*fac*-**13** was prepared by using a procedure similar to that for Δ-*fac*-**6**. A 5 N NaOH solution in H_2_O (500 μL) was added to a solution of Δ-*fac*-**14** (10 mg, 6.3 μmol) in MeOH (2 mL), and the reaction mixture was stirred at 80 °C for 4 days. After cooling to room temperature, the solvent was removed under reduced pressure. After saturated aqueous NaHCO_3_ was added, the solution was washed with AcOEt. To the aqueous layer was added a 2 N aqueous solution of HCl to adjust the pH to 1, and the resulting solution was extracted with AcOEt. The combined organic layer was washed with brine, dried over Na_2_SO_4_, filtered, and concentrated under reduced pressure to afford Δ-*fac*-**13** (7 mg, quantitative, >99% ee) as a orange solid. Mp: 297 °C (dec). IR (ATR): ν 2921, 2851, 2524, 2161, 1672, 1573, 1548, 1500, 1407, 1377, 1348, 1244, 1165, 1094, 1029, 914, 801, 752, 737, 699, 674, 632, 603, 580, 558, 516, 479, 460, 445, 429, 418, 406 cm^–1^. ^1^H NMR (600 MHz, DMSO-*d*_6_/TMS): δ 12.26 (bs, 3H), 8.87 (d, *J* = 8.4 Hz, 3H), 8.75 (s, 3H), 7.99 (dd, *J* = 8.4, 1.2 Hz, 3H), 7.88 (td, *J* = 7.8, 1.2 Hz, 3H), 7.84 (td, *J* = 7.2, 1.2 Hz, 3H), 7.56 (d, *J* = 6.0 Hz, 3H), 7.40 (d, *J* = 6.6 Hz, 3H), 2.26 (s, 9H) ppm. ^13^C NMR (150 MHz, DMSO-*d*_6_/TMS): δ 171.1, 169.1, 164.8, 143.4, 140.3, 139.5, 139.3, 136.5, 131.6, 131.2, 130.7, 129.0, 127.8, 126.0, 125.6, 121.6, 22.2 ppm. ESI-MS (*m*/*z*): calcd for C_51_H_36_^191^IrN_3_O_6_ [M]^+^ 977.2205; found 977.2206.

#### Λ-*fac*-**13**

Λ-*fac*-**13** was prepared by using a procedure similar to that for Δ-*fac*-**6**. A 5 N aqueous NaOH solution (200 μL) was added to a solution of Λ-*fac*-**14** (10 mg, 6.3 μmol) in MeOH (800 μL), and the reaction mixture was stirred at 80 °C for 3 h. After cooling to room temperature, the solvent was removed under reduced pressure. After saturated aqueous NaHCO_3_ was added, the solution was washed with AcOEt. To the aqueous layer was added a 2 N aqueous solution of HCl to adjust the pH to 1, and the resulting solution was extracted with AcOEt. The combined organic layer was washed with brine, dried over Na_2_SO_4_, filtered, and concentrated under reduced pressure to afford Λ-*fac*-**13** (8 mg, quantitative, >99% ee) as an orange solid. Mp: 297 °C (dec). IR (ATR): ν 2923, 2852, 2512, 2162, 2034, 1672, 1574, 1547, 1518, 1501, 1434, 1377, 1348, 1269, 1244, 1165, 1094, 1031, 915, 813, 781, 752, 737, 700, 674, 632, 602, 557, 499, 483, 461, 440, 416, 423, 410 cm^–1^. ^1^H NMR (600 MHz, DMSO-*d*_6_/TMS): δ 12.26 (bs, 3H), 8.87 (d, *J* = 8.4 Hz, 3H), 8.75 (s, 3H), 7.99 (dd, *J* = 8.1, 1.2 Hz, 3H), 7.88 (td, *J* = 7.8, 1.8 Hz, 3H), 7.84 (td, *J* = 7.5, 1.2 Hz, 3H), 7.56 (d, *J* = 6.0 Hz, 3H), 7.40 (d, *J* = 6.6 Hz, 3H), 6.71 (s, 3H), 2.26 (s, 9H) ppm. ^13^C NMR (150 MHz, DMSO-*d*_6_/TMS): δ 168.9, 143.3, 140.2, 139.4, 139.2, 136.3, 131.5, 131.1, 128.9, 127.7, 125.9, 125.5, 121.4, 22.1 ppm. ESI-MS (*m*/*z*): calcd for C_51_H_36_^191^IrN_3_O_6_ [M]^+^ 977.2205; found 977.2207.

### X-ray Data Collection and Refinement of Δ-*fac*-**9**, Δ-*fac*-**11**, and Λ-*fac*-**11**

Yellow crystals of Δ-*fac*-**9**, Δ-*fac*-**11**, and Λ-*fac*-**11** suitable for X-ray analysis were obtained by the slow vapor diffusion of hexanes into their CHCl_3_ solutions at room temperature. The synchrotron X-ray diffraction study for Δ-*fac*-**9** was carried out at the BL02B1 beamline at Spring-8 with the approval of the Japan Synchrotron Radiation Research Institute (JASRI) with a diffractometer equipped with a Rigaku Mercury2CCD detector. The collected diffraction data were processed with the *RAPID AUTO* software program. The structure was solved by the charge-flipping method and refined by full-matrix least squares on *F*^2^ by using the SHELX program suite. The single-crystal X-ray diffraction study for Δ-*fac*-**11** and Λ-*fac*-**11** was carried out using a Rigaku X-ray diffractometer equipped with a molybdenium MicroMax-007 and Saturn 724+ detector, and the measurement was performed at 293 K. The structure was solved by direct methods (SHELX) and refined by full-matrix least squares methods on *F*^2^ using the Yadokari-XG program. OMIT was used to exclude the most disagreeable reflections (error/esd >10) for the refinement of Δ-*fac*-**9**, whereas DELU, SIMU, and ISOR were used for the refinement of Δ-*fac*-**11**. All non-hydrogen atoms were refined anisotropically in the structure. The crystal data in this manuscript can be obtained free of charge from The Cambridge Crystallographic Data Centre via www.ccdc.cam.ac.uk/data_request/cif.

#### Crystal data for Δ-*fac*-**9**:

formula C_38_H_47_Cl_6_Ir_0.5_N_4.5_O_4.5_; FW = 947.60, triclinic, space group *P*1, *a* = 13.5883(6) Å, *b* = 14.0191(7) Å, *c* = 14.0425(7) Å, α = 101.692(7)°, β = 115.010(8)°, γ = 110.375(8)°, *V* = 2070.9(2) Å^3^, *Z* = 2, *T* = 100 K, *D*_calcd_ = 1.520 g cm^–3^, μ(synchrotron) = 0.503 cm^–1^, 2θ_max_ = 16.00°, λ(synchrotron) = 0.4126 Å, 62001 reflections measured, 18641 unique, 18451 (*I* > 2σ(*I*)) were used to refine 976 parameters, 3 restraints, wR2 = 0.1223, R1 = 0.0459 (*I* > 2σ(*I*)), GOF = 1.020. A total of 62001 reflections were collected, among which 616 reflections were independent (*R*_int_ = 0.0466). CCDC 2236350 contains the supplementary crystallographic data for the sample, and representative parameters are also given in Table S1 in the Supporting Information.

#### Crystal data for Δ-*fac*-**11**:

formula C_76_H_91_Cl_12_IrN_6_O_12_; FW = 1898.14, triclinic, space group *P*1, *a* = 13.3507(3) Å, *b* = 13.9918(3) Å, *c* = 14.3988(3) Å, α = 108.231(2)°, β = 113.667(2)°, γ = 104.028(2)°, *V* = 2120.17(9) Å^3^, *Z* = 1, *T* = 293 K, *D*_calcd_ = 1.487 g cm^–3^, μ(Mo Kα) = 2.013 cm^–1^, 2θ_max_ = 30.2610°, λ(Mo Kα) = 0.71073 Å, 42682 reflections measured, 19538 unique, 17790 (*I* > 2σ(*I*)) were used to refine 976 parameters, 3 restraints, wR2 = 0.0974, R1 = 0.0438 (*I* > 2σ(*I*)), GOF = 0.992. A total of 42682 reflections were collected, among which 17790 reflections were independent (*R*_int_ = 0.0432). CCDC 2119640 contains the supplementary crystallographic data for the product, and representative parameters are also given in Table S1 in the Supporting Information.

#### Crystal data for Λ-*fac*-**11**:

formula C_86_H_117_Cl_6_IrN_6_O_12_; FW = 1831.75, orthorhombic, space group *P*2_1_2_1_2_1_, *a* = 14.2878(3) Å, *b* = 21.3264(4) Å, *c* = 30.3367(6) Å, α = 90°, β = 90°, γ = 90°, *V* = 9243.8(3) Å^3^, *Z* = 4, *T* = 293 K, *D*_calcd_ = 1.316 g cm^–3^, μ(Mo Kα) = 1.676 cm^–1^, 2θ_max_ = 30.2970°, λ(Mo Kα) = 0.71073 Å, 93431 reflections measured, 25814 unique, 20843 (*I* > 2σ(*I*)) were used to refine 1012 parameters, 188 restraints, wR2 = 0.1261, R1 = 0.0540 (*I* > 2σ(*I*)), GOF = 1.082. A total of 93431 reflections were collected, among which 34850 reflections were independent (*R*_int_ = 0.0466).

### Measurements of UV–Vis Absorption and Luminescence Spectra

UV–vis spectra were recorded on a JASCO V-630 spectrometer, and emission spectra were recorded at 25 °C on a JASCO FP-8300 spectrofluorometer. Sample solutions in quartz cuvettes equipped with Teflon septum screw caps were degassed by bubbling Ar through the solution for 10 min prior to luminescence measurements. The luminescence quantum yields (Φ) were determined by comparison with the integrated corrected emission spectrum of quinine sulfate (Φ = 0.55 in 0.1 M H_2_SO_4_) when excited at 366 nm. Equation 1 was used for calculating the emission quantum yields, in which Φ_s_ and Φ_r_ denote the quantum yields of the sample and reference of the solvents used for the measurements of the sample and reference compound, respectively, and η_s_ and η_r_ are the refractive indexes of the solvents used for the measurements of the sample and reference, respectively (η: 1.33 for H_2_O, 1.42 for CH_2_Cl_2_, 1.50 for toluene, and 1.48 for DMSO). *A*_s_ and *A*_r_ denote the absorbances of the sample and reference, respectively.
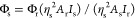
1

The luminescence lifetimes of sample solutions in degassed DMSO or aqueous solutions at 298 K were measured on a TSP1000-M-PL (Unisoku, Osaka, Japan) instrument using THG (355 nm) of a Nd:YAG laser Minilite I (Continuum, CA) as the excitation source. The signals were monitored with an R2949 photomultiplier. Data were analyzed by using a nonlinear least-squares procedure.

### Measurements of CD Spectra and CPL Spectra

CD spectra were recorded on a Chirascan (Applied Photophysics) spectrophotometer, and CPL spectra were measured using a JASCO CPL-300 spectrofluoropolarimeter.

### Stability of *fac*-**9** and *fac*-**11** against Light and Heat and in the Presence of Silica Gel

Δ- and Λ-*fac*-**9** and -**11** were dissolved in toluene (1 mM), respectively. For photostability, each solution was irradiated at room temperature with light at a wavelength of 365 nm for 1 h using Twinlight (RELYON, Tokyo, Japan), equipped with an LED (365 nm). For stability against heat, each solution was heated to 100 °C for 1 h. For stability in the presence of silica gel, silica gel was added to each solution, and the mixture was then stirred at room temperature for 1 h. Each solution was then filtered and analyzed by normal-phase HPLC (SenshuPak PEGASIL Silica SP100, eluent CHCl_3_/MeCN = 1/2 for *fac*-**9**, hexanes/CHCl_3_ = 1/5 for *fac*-**11**; flow rate 1.0 mL/min; UV detection at 254 nm).

### Theoretical Calculations

Density functional theory (DFT) calculations were carried out using the Gaussian09 program (PBE1PBE functional, the LanL2DZ basis set for Ir and the 6-31G basis set for H, C, O, and N atoms). The molecular orbitals were visualized using Jmol software (an open-source Java viewer for chemical structures in 3D, http://www.jmol.org/).

## Supporting Information Available

Predicted most stable structures of Δ-*fac*-**9**, Λ-*fac*-**9**, Δ-*fac*-**11**, and Λ-*fac*-**11**, energy diagram for the HOMO and LUMO of *fac*-**5**, Δ*-fac***-9**, Λ*-fac***-9**, Δ*-fac***-11**, and Λ*-fac***-11**, Gibbs free energies of the most stable structures of Δ*-fac***-9**, Λ*-fac***-9**, Δ*-fac***-11**, and Λ*-fac***-11**, HPLC chromatograms of carboxylic acid derivatives, crystal data and structure refinement for Δ-*fac*-**9**, Δ-*fac*-**11**, and Λ-*fac*-**11**, representative parameters for the crystal structure analysis of Δ*-fac***-9**, Δ*-fac***-11**, and Λ*-fac***-11**, photophysical properties of *fac*-**4**, *fac*-**6**, and *fac*-**13** in degassed DMSO, CPL spectra of Δ- and Λ-*fac*-**4** and -**6**, and ^1^H NMR and ^13^C NMR spectra of racemic *fac*-**13** and Δ- and Λ-*fac*-**9**, -**10**, -**11**, -**14**, -**6**, -**4**, and -**13** (PDF)
