# Correction to “Optical Resolution of Carboxylic Acid Derivatives of Homoleptic Cyclometalated Iridium(III) Complexes via Diastereomers Formed with Chiral Auxiliaries”

**DOI:** 10.1021/acs.inorgchem.3c02577

**Published:** 2023-08-10

**Authors:** Azusa Kanbe, Kenta Yokoi, Yasuyuki Yamada, Makoto Tsurui, Yuichi Kitagawa, Yasuchika Hasegawa, Daiji Ogata, Junpei Yuasa, Shin Aoki

Page 11326: The chemical structures of Δ-**2**, Λ-**2**, Δ-**3**, and Λ-**3** in Chart 1 in the published manuscript are corrected as follows. This new figure differs from the original with respect to the stereochemistry of Δ-**2**, Λ-**2**, Δ-**3**, and Λ-**3**. Namely, the stereochemistry of two pyridyl rings of 2-phenylpyridine (ppy) and 4,6-difluorophenylpyridine ligands (two Ir–N coordination bonds) in **2** and **3** were drawn in *cis* positions around Ir cation in our original publication, and it is corrected to *trans* positions in this correction. This change does not impact the object, results, and conclusions in the original publication.

**Chart 1:**
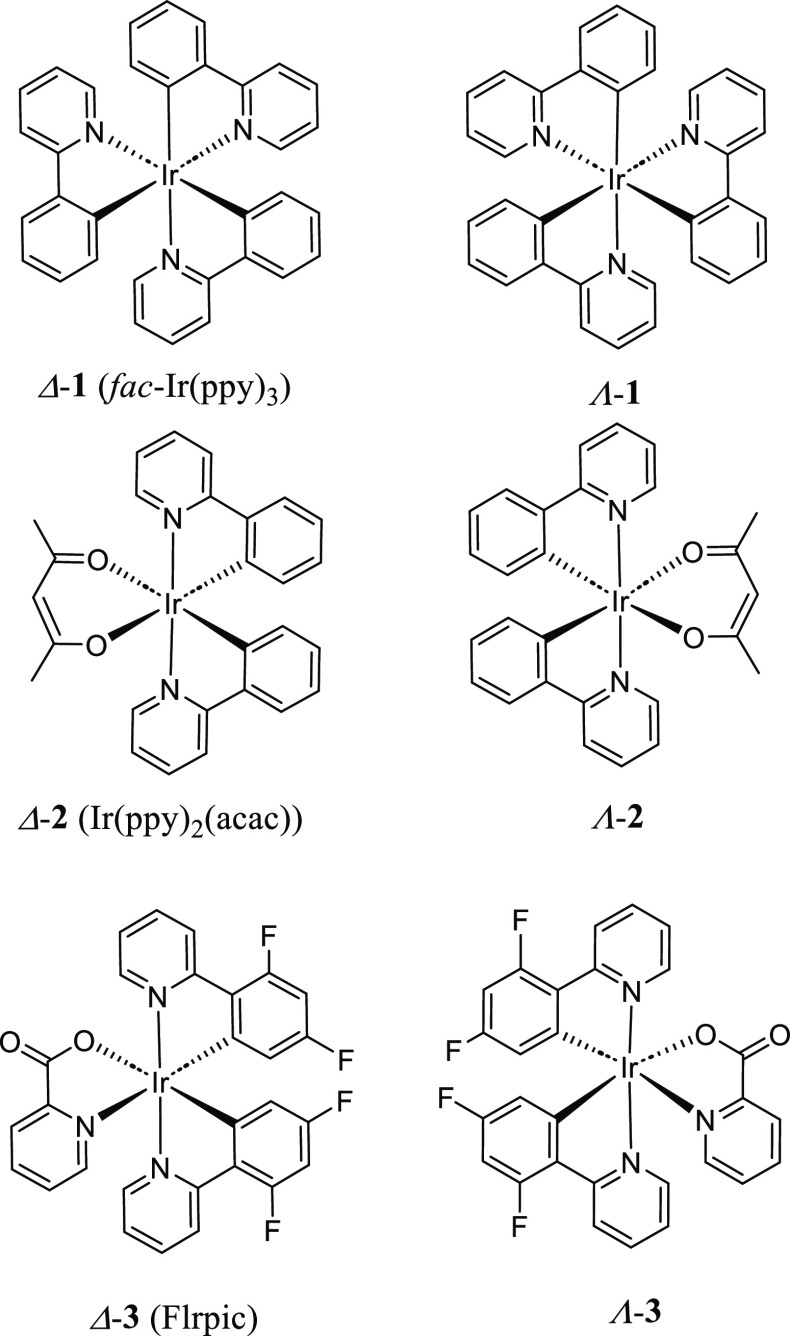
Chemical Structures and Absolute Configuration of Representative Cyclometalated Ir(III) Complexes

