# Supplementary material for: Optical Resolution of Carboxylic Acid Derivatives of Homoleptic Cyclometalated Iridium(III) Complexes via Diastereomers Formed with Chiral Auxiliaries
Source: Inorg Chem. 2023 Jul 11;62(29):11325–41. doi: 10.1021/acs.inorgchem.3c00685 (PMC10369494; doi:10.1021/acs.inorgchem.3c00685)
Supplement: Supplementary file 1 — ic3c00685_si_001.pdf [file ic3c00685_si_001.pdf]

Optical Resolution of Carboxylic Acid Derivatives of Homoleptic Cyclometalated Iridium(III) Complexes via Diastereomers Formed with Chiral Auxiliaries

*Azusa Kanbe,[a] Kenta Yokoi,[a] Yasuyuki Yamada,[b,c,d] Makoto Tsurui,[e] Yuichi Kitagawa,[f,g] Yasuchika Hasegawa,[f,g] Daiji Ogata,[h] Junpei Yuasa,[h] and Shin Aoki [a,i,j]**

[a]Faculty of Pharmaceutical Science, Tokyo University of Science,

2641 Yamazaki, Noda, Chiba 278-8510, Japan,

Phone no. +81-4-7121-3670

[b]Department of Chemistry, Graduate School of Science, Nagoya University,

Furo-cho, Chikusa-ku, Nagoya 464-8602, Japan

[c]Research Center for Materials Science, Nagoya University,

Furo-cho, Chikusa-ku, Nagoya 464-8602, Japan

[d]JST, PRESTO, 4-1-8 Honcho, Kawaguchi, Saitama, 332-0012, Japan

[e]Graduate School of Chemical Sciences and Engineering, Hokkaido University,

N13W8, Kita-ku, Sapporo, Hokkaido, 060-8628, Japan

[f]Faculty of Engineering, Hokkaido University,

Kita-13, Nishi-8, Kita-Ku, Sapporo, Hokkaido 060-8628, Japan

[g]Institute for Chemical Reaction Design and Discovery (WPI-ICReDD), Hokkaido University,

Kita-21, Nishi-10, Kita-Ku, Sapporo, Hokkaido 001-0021, Japan

[h]Faculty of Science, Tokyo University of Science,

1-3 Kagurazaka, Shinjuku-ku, Tokyo 162-8601, Japan,

[i]*Research Institute for Science and Technology (RIST),*

Tokyo University of Science, 2641 Yamazaki, Noda, Chiba 278-8510, Japan

[j]*Research Institute for Biomedical Science (RIBS),*

Tokyo University of Science, 2641 Yamazaki, Noda, Chiba 278-8510, Japan,

***Corresponding authors: E-mail,** shinaoki@rs.tus.ac.jp

Table of Contents

Figure S1. Predicted most stable structures of *Δ-fac*-9, *Λ-fac*-9, *Δ-fac*-11, and *Λ-fac*-11 .... S3

Figure S2. Energy diagram for the HOMO and LUMO of *fac*-5, *Δ-fac*-9, *Λ-fac*-9, *Δ-fac*-11, and *Λ-fac*-11 .... S4

Figure S3. Gibbs free energies of the most stable structures of *Δ-fac*-9, *Λ-fac*-9, *Δ-fac*-11, and *Λ-fac*-11 .... S4

Figure S4 HPLC chromatograms of carboxylic acid derivatives........ S5

Table S1. Crystal data and structure refinement for *Δ-fac*-9, *Δ-fac*-11, and *Λ-fac*-11........... S6

Table S2. Representive parameters for the crystal structure analysis of *Δ-fac*-9, *Δ-fac*-11, and *Λ-fac*-11 .... S6

Table S3. Photophysical Properties of *fac*-4, *fac*-6, and *fac*-13 in Degassed DMSO S7

Figure S5. CPL spectra of *Δ*- and *Λ-fac*-4 and 6 .... S7

Figure S6. ^{1}H NMR and ^{13}C NMR spectra of *Δ-fac*-9 .... S8

Figure S7. ^{1}H NMR and ^{13}C NMR spectra of *Λ-fac*-9 .... S9

Figure S8. ^{1}H NMR and ^{13}C NMR spectra of *Δ-fac*-10 .... S10

Figure S9. ^{1}H NMR and ^{13}C NMR spectra of *Λ-fac*-10 .... S11

Figure S10. ^{1}H NMR and ^{13}C NMR spectra of *Δ-fac*-11 .... S12

Figure S11. ^{1}H NMR and ^{13}C NMR spectra of *Λ-fac*-11 .... S13

Figure S12. ^{1}H NMR and ^{13}C NMR spectra of racemic *fac*-13 .... S14

Figure S13. ^{1}H NMR and ^{13}C NMR spectra of *Δ-fac*-14 .... S15

Figure S14. ^{1}H NMR and ^{13}C NMR spectra of *Λ-fac*-14 .... S16

Figure S15. ^{1}H NMR and ^{13}C NMR spectra of *Δ-fac*-6 .... S17

Figure S16. ^{1}H NMR and ^{13}C NMR spectra of *Λ-fac*-6 .... S18

Figure S17. ^{1}H NMR and ^{13}C NMR spectra of *Δ-fac*-4 .... S19

Figure S18. ^{1}H NMR and ^{13}C NMR spectra of *Λ-fac*-4 .... S20

Figure S19. ^{1}H NMR and ^{13}C NMR spectra of *Δ-fac*-13 .... S21

Figure S20. ^{1}H NMR and ^{13}C NMR spectra of *Λ-fac*-13 .... S22

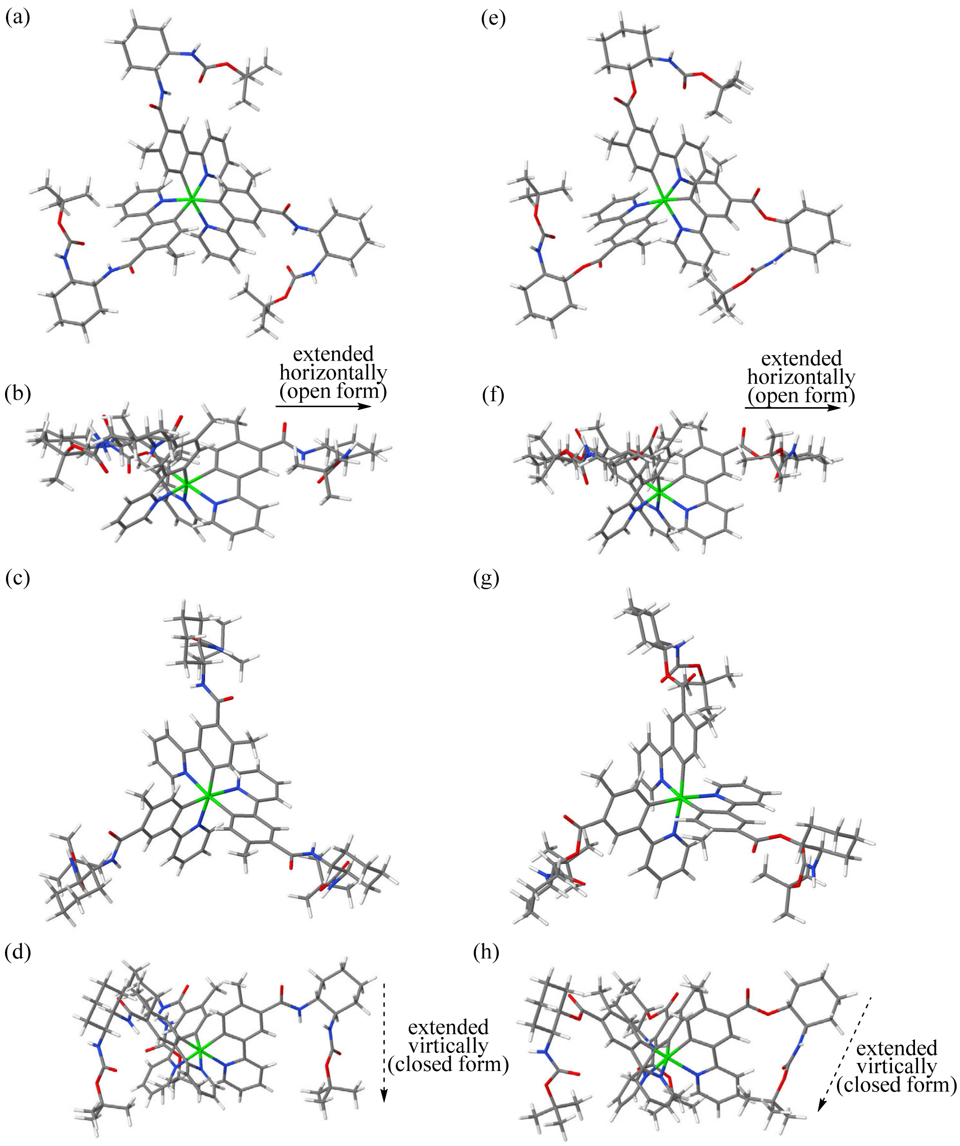

Figure S1. Predicted most stable structures of *Δ-fac*-**9**, *Λ-fac*-**9**, *Δ-fac*-**11**, and *Λ-fac*-**11** calculated by DFT methods using the B3LYP hybrid functional together with the LanL2DZ basis set for an Ir atom and the 6-31G basis set for H, C, O, and N atoms. (a and b) top view and side view of *Δ-fac*-**9**, (c and d) top view and side view of *Λ-fac*-**9**, (e and f) top view and side view of *Δ-fac*-**11**, (g and h) top view and side view of *Λ-fac*-**11**.

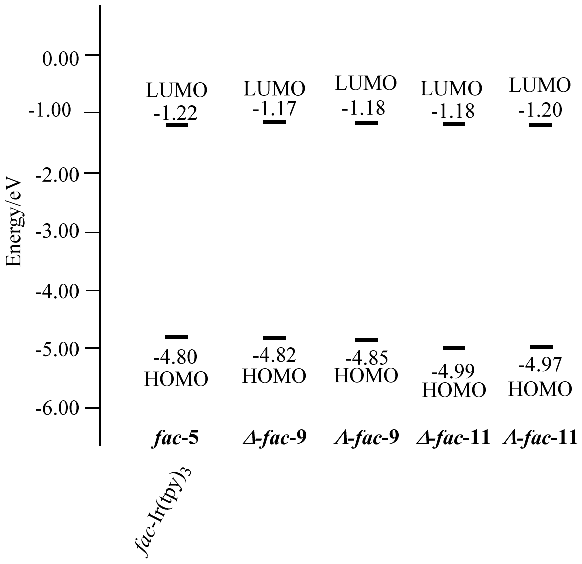

Figure S2. Energy diagram for the HOMO and LUMO of *fac*-**5**, *Δ*-*fac*-**9**, *Λ*-*fac*-**9**, *Δ*-*fac*-**11**, and *Λ*-*fac*-**11** calculated by the Gaussian09 program using the B3LYP hybrid functional together with the LanL2DZ basis set for the Ir atom and the 6-31G basis set for the H, C, N, and O atoms.

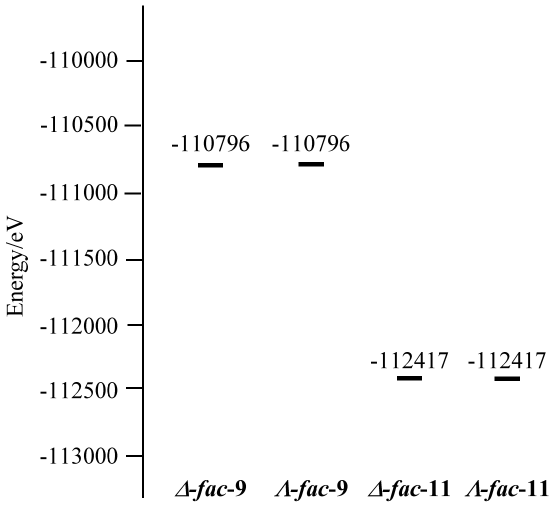

Figure S3. Gibbs free energies of the most stable structures of *Δ*-*fac*-**9**, *Λ*-*fac*-**9**, *Δ*-*fac*-**11**, and *Λ*-*fac*-**11** calculated by the Gaussian09 program using the B3LYP hybrid functional together with the LanL2DZ basis set for the Ir atom and the 6-31G basis set for the H, C, N, and O atoms.

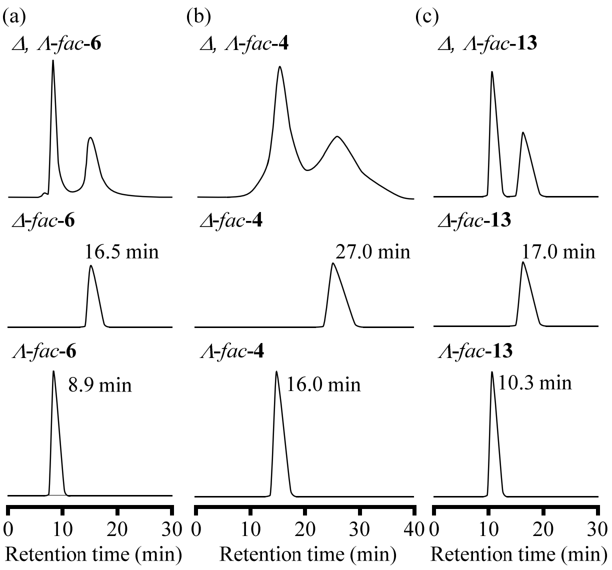

Figure S4. HPLC chromatograms of carboxylic acid derivatives on CHIRALCEL® OJ-H (racemic mixture: top, *Δ*-forms: middle, and *Λ*-forms: bottom). (a) HPLC chromatograms of *fac*-**6**. Eluent: Hexane/EtOH = 50/50; flow rate: 1.0 mL/min; UV detection at 254 nm. (b) HPLC chromatograms of *fac*-**4**. Eluent: Hexane/EtOH = 30/70; flow rate: 0.5 mL/min; UV detection at 254 nm. (c) HPLC chromatograms of *fac*-**13**. Eluent: Hexane/EtOH = 50/50; flow rate: 1.0 mL/min; UV detection at 254 nm.

Table S1. Crystal Data and Structure Refinement for *Δ-fac*-**9**, *Δ-fac*-**11**, and *Λ-fac*-**11**.

Compound	*Δ-fac*-**9**	*Δ-fac*-**11**	*Λ-fac*-**11**
CCDC Deposition Number	2236350	2119640	2114777
Empirical formula	$C_{38}H_{47}Cl_6Ir_{0.5}N_{4.5}O_{4.5}$	$C_{76}H_{91}Cl_{12}IrN_6O_{12}$	$C_{86}H_{117}Cl_6IrN_6O_{12}$
Formula weight	947.60	1898.14	1831.75
Temperature	100 K	293 K	293 K
Crystal system	Triclinic	Triclinic	Orthorhombic
Space group	P 1	P 1	P 21 21 21
Unit cell dimensions	a = 13.5883(6) Å	a = 13.3507(3) Å	a = 14.2878(3) Å
	b = 14.0191(7) Å	b = 13.9918(3) Å	b = 21.3264(4) Å
	c = 14.0425(7) Å	c = 14.3988(3) Å	c = 30.3367(6) Å
	α = 101.692(7)	α = 108.231(2)	α = 90
	β = 115.010(8)	β = 113.667(2)	β = 90
	γ = 110.375(8)	γ = 104.028(2)	γ = 90
Volume	2070.9(2) $Å^3$	2120.17(9) $Å^3$	9243.8(3) $Å^3$
Z	2	1	4
Density (calcd.)	1.520 g cm^{-1}	1.487 g cm^{-1}	1.316 g cm^{-1}
Absorption coefficient	0.503 cm^{-1}	2.013 cm^{-1}	1.676 cm^{-1}
F(000)	966	966	3800
Crystal size	0.06×0.04×0.02 mm^3	0.06×0.05×0.01 mm^3	0.2×0.14×0.1 mm^3
Theta range for data collection	6.535 to 16.00°	3.0900 to 30.2610°	2.9070 to 30.2970°
Index ranges	-17<=h<=17 -18<=k<=18 -18<=l<=18	-18<=h<=19 -18<=k<=19 -20<=l<=20	-20<=h<=18 -30<=k<=31 -44<=l<=42
Reflections collected	62001	42682	93431
Independent reflections	18641 (Rint = 0.0466)	19538 (Rint = 0.0676)	25814 (Rint = 0.0466)
Completeness to theta	1.96/0.98 (theta = 15.540°)	1.41/0.70 (theta = 31.290°)	1.59/0.86 (theta = 31.279°)
Absorption correction	multi-scan	multi-scan	multi-scan
Refinement method	Full-matrix least-square on F^2	Full-matrix least-square on F^2	Full-matrix least-square on F^2
Goodness-of-fit on F2	1.020	0.743	1.082
Final R indices [I>2sigma(I)]	R_1 = 0.0459	R_1 = 0.0438	R_1 = 0.0540
	wR_2 = 0.1217	wR_2 = 0.0952	wR_2 = 0.1193
R indices (all data)	R_1 = 0.0463	R_1 = 0.0500	R_1 = 0.0732
	wR_2 = 0.1223	wR_2 = 0.0974	wR_2 = 0.1261

Table S2. Representative Parameters for the Crystal Structure Analysis of *Δ-fac*-**9**, *Δ-fac*-**11**, and *Λ-fac*-**11**.

		Δ-fac-**9**	*Δ-fac*-**11**	*Λ-fac*-**11**
bond lengths (Å) (averaged values)	C (2')–Ir	2.00	1.99	2.01
	N (1)–Ir	2.12	2.14	2.13
dihedral angles (deg) (averaged values)	C–Ir–C	94.2	95.4	95.5
	C–Ir–N	89.9	89.9	89.6
	N–Ir–N	96.2	95.5	95.6

Table S3. Photophysical Properties of *fac*-**4**, *fac*-**6**, and *fac*-**13** in Degassed DMSO at 298 K ([compound] = 10 μM)

compound	λ_{abs} (nm) (ε ($M^{-1}cm^{-1}$))	λ_{em} (nm)[a]	Φ[a]	τ (*us*)
Δ-fac-**4**	283 (6.1 × 10^4), 320 (2.5 × 10^4)	489	0.58[b]	1.28[d]
Λ-fac-**4**	283 (5.8 × 10^4), 320 (2.5 × 10^4)	489	0.57[b]	1.27[d]
Δ-fac-**6**	286 (4.5 × 10^4)	491	0.57[b]	1.35[d]
Λ-fac-**6**	286 (4.8 × 10^4)	490	0.60[b]	1.35[d]
Δ-fac-**13**	279 (2.2 × 10^4), 314 (2.0 × 10^4), 400 (5.4 × 10^3)	591	0.42[c]	2.06[e]
Λ-fac-**13**	279 (1.9 × 10^4), 316 (2.0 × 10^4), 402 (5.4 × 10^3)	590	0.42[c]	2.03[e]

[a]Excitation at 366 nm. [b]Quinine sulfate in 0.1 M H_2SO_4 (Φ = 0.55) was used as a reference. [c]*fac*-Ir(mpiq)$_3$ in toluene (Φ = 0.26) was used as a reference. [d]A 475 nm long wave pass filter was used. [d]A 550 nm long wave pass filter was used.

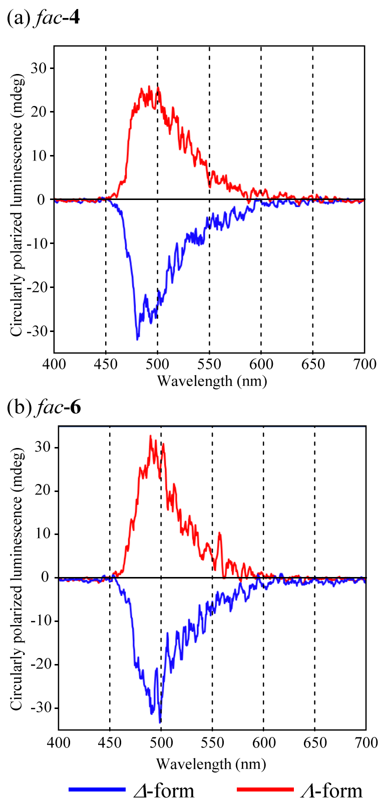

Figure S5. CPL spectra of (a) *fac*-**4** and (b) *fac*-**6** (100 μM) in DMSO (excitation at 290 nm for *fac*-**4** and **6**) at 298 K. Blue curves: *Δ*-forms and red curves: *Λ*-forms.

(a) ^{1}H NMR

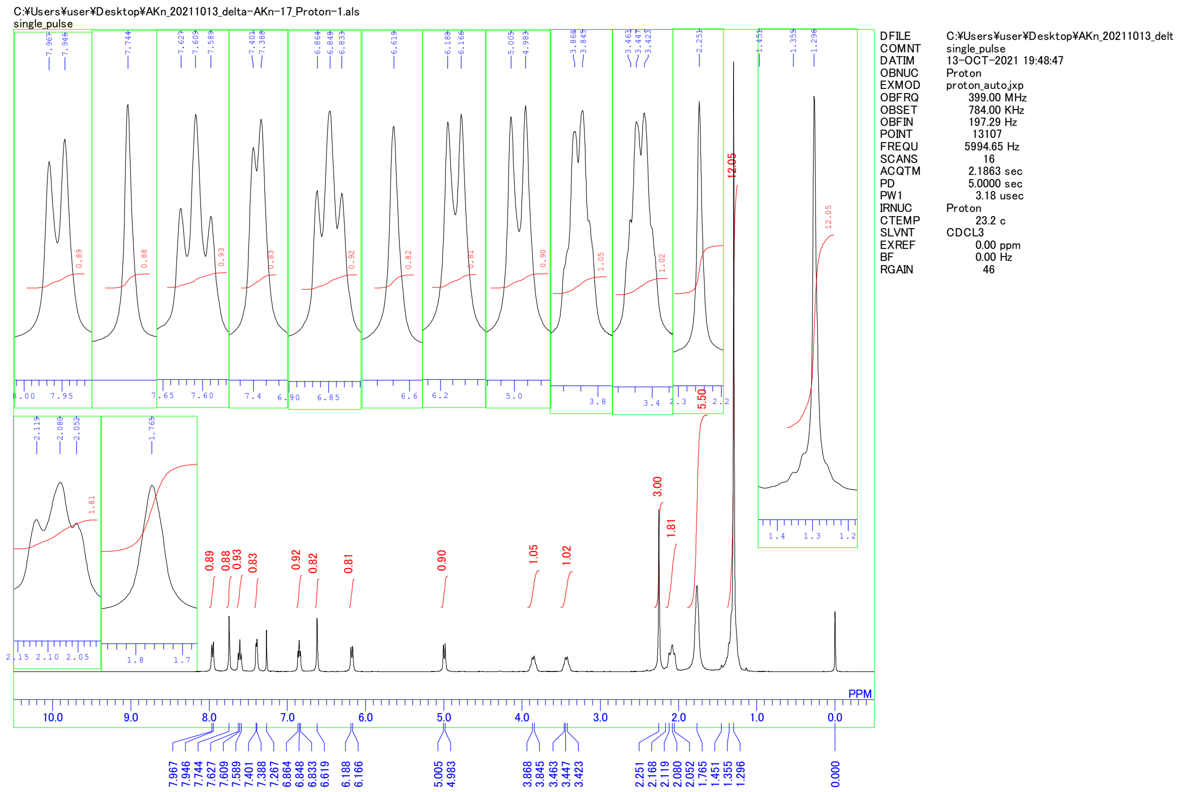

(b) ^{13}C NMR

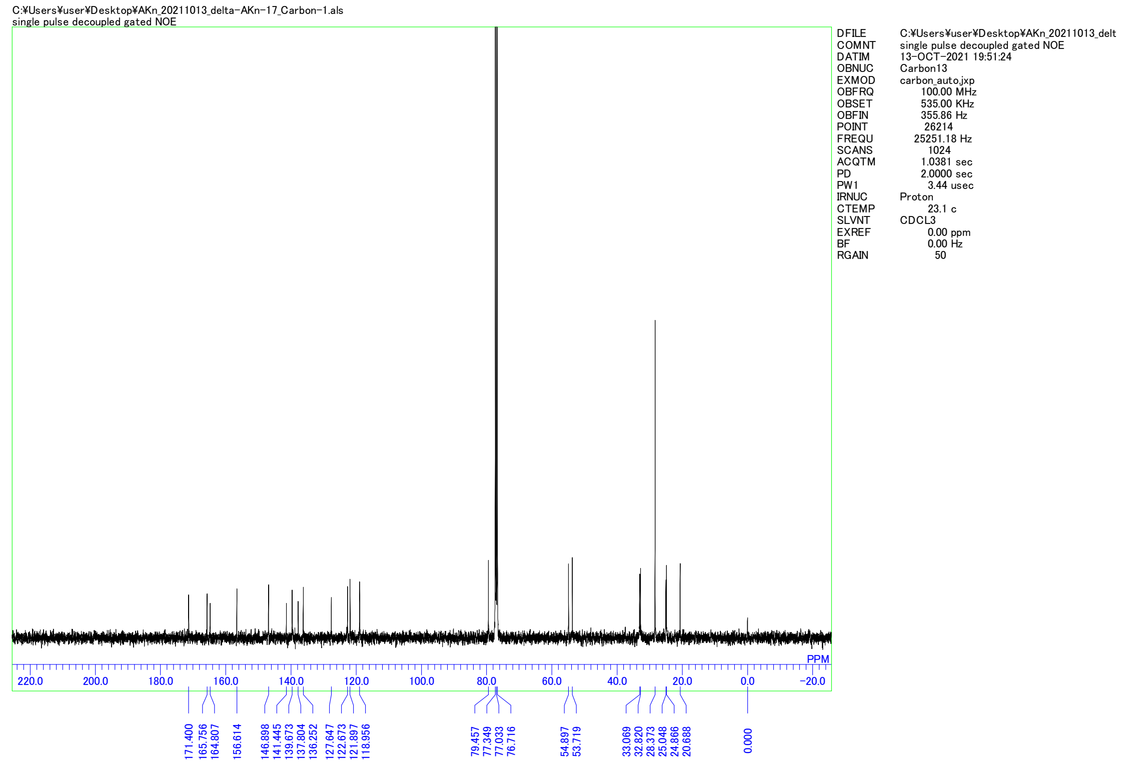

Figure S6. ^{1}H NMR (a) and ^{13}C NMR (b) spectra of *Δ*-*fac*-**9**.

(a) ^{1}H NMR

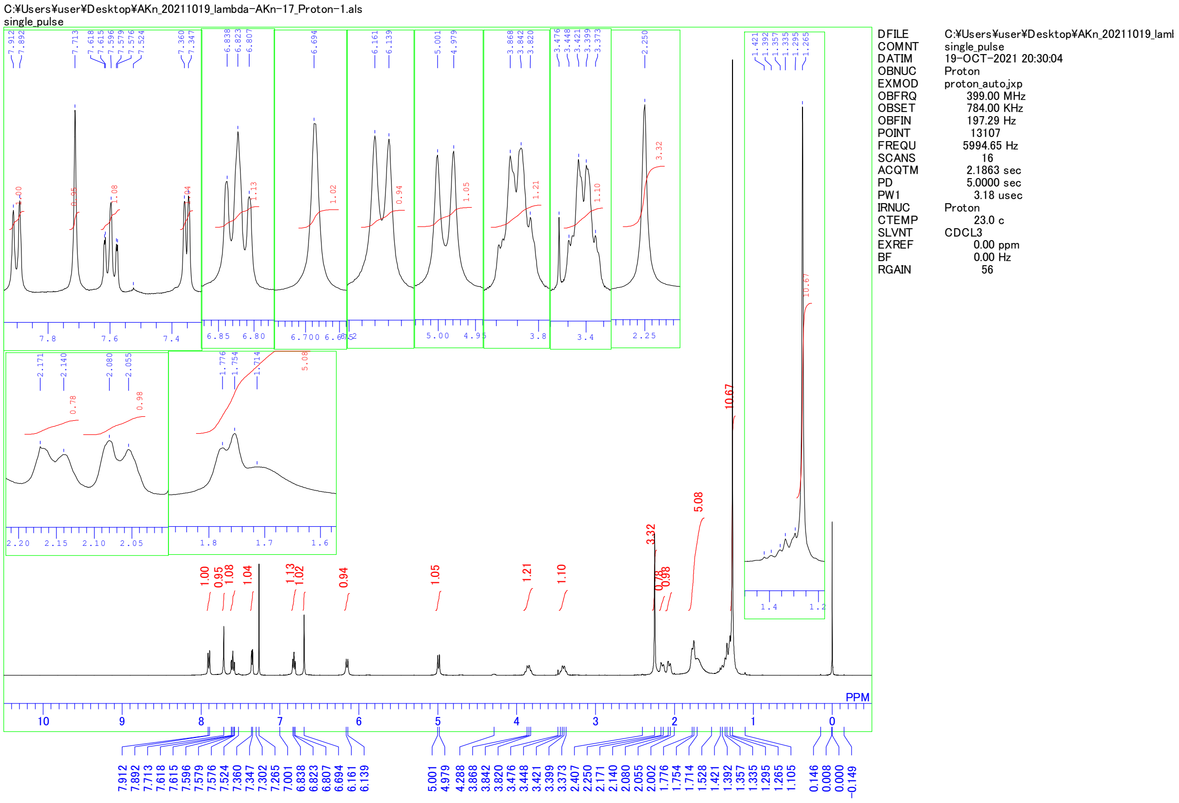

(b) ^{13}C NMR

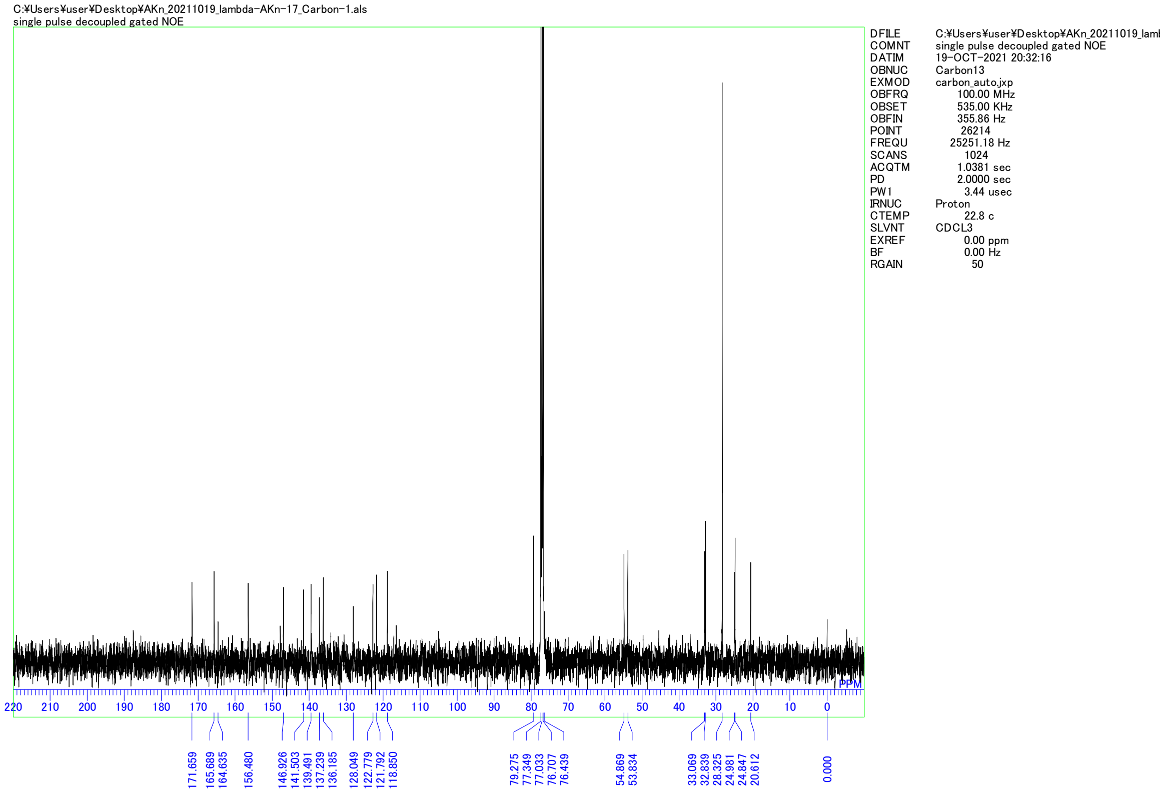

Figure S7. ^{1}H NMR (a) and ^{13}C NMR (b) spectra of *Λ*-*fac*-**9**.

(a) ^{1}H NMR

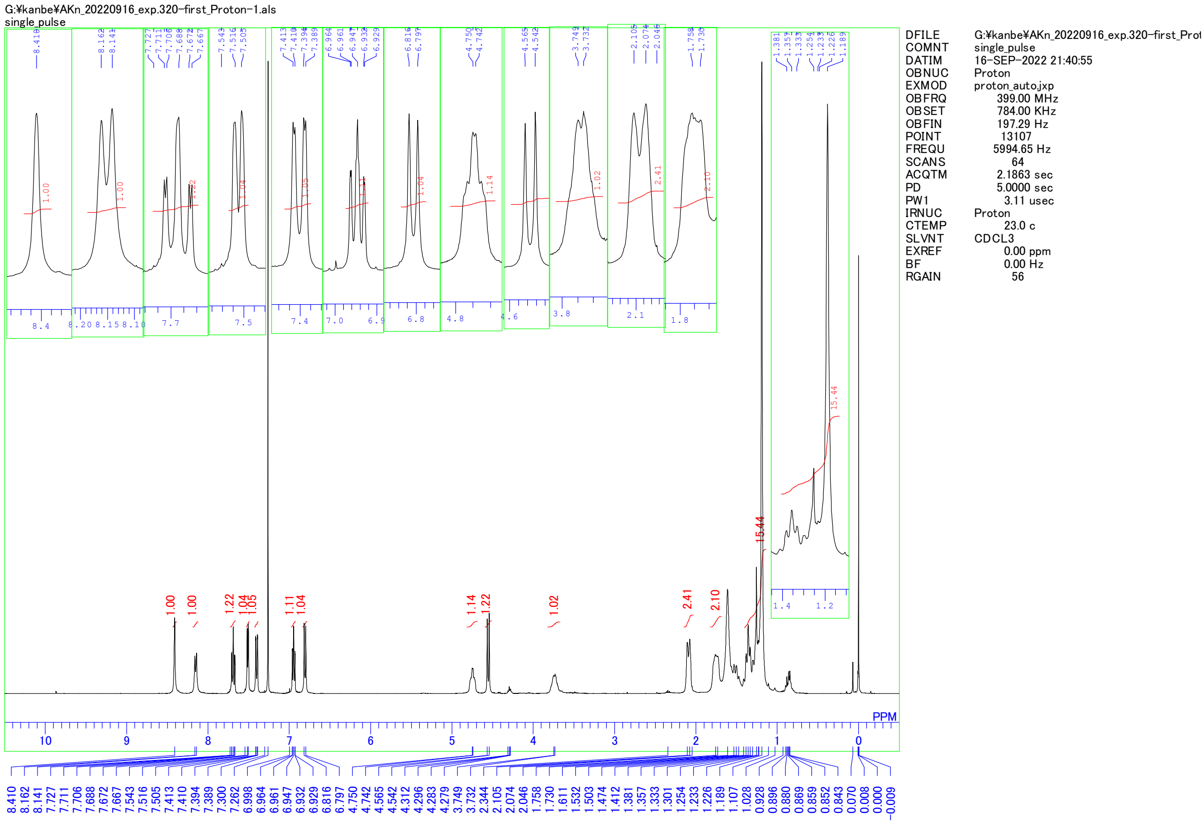

(b) ^{13}C NMR

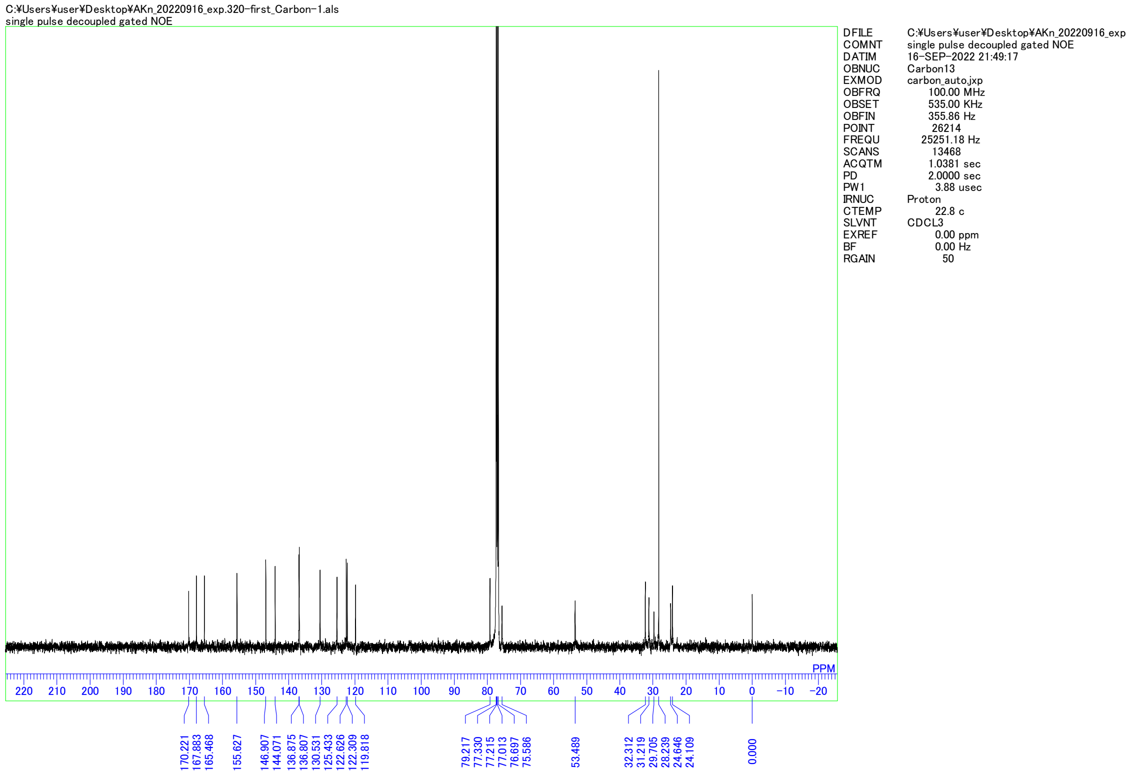

Figure S8. ^{1}H NMR (a) and ^{13}C NMR (b) spectra of *Δ-fac*-**10**.

(a) ^{1}H NMR

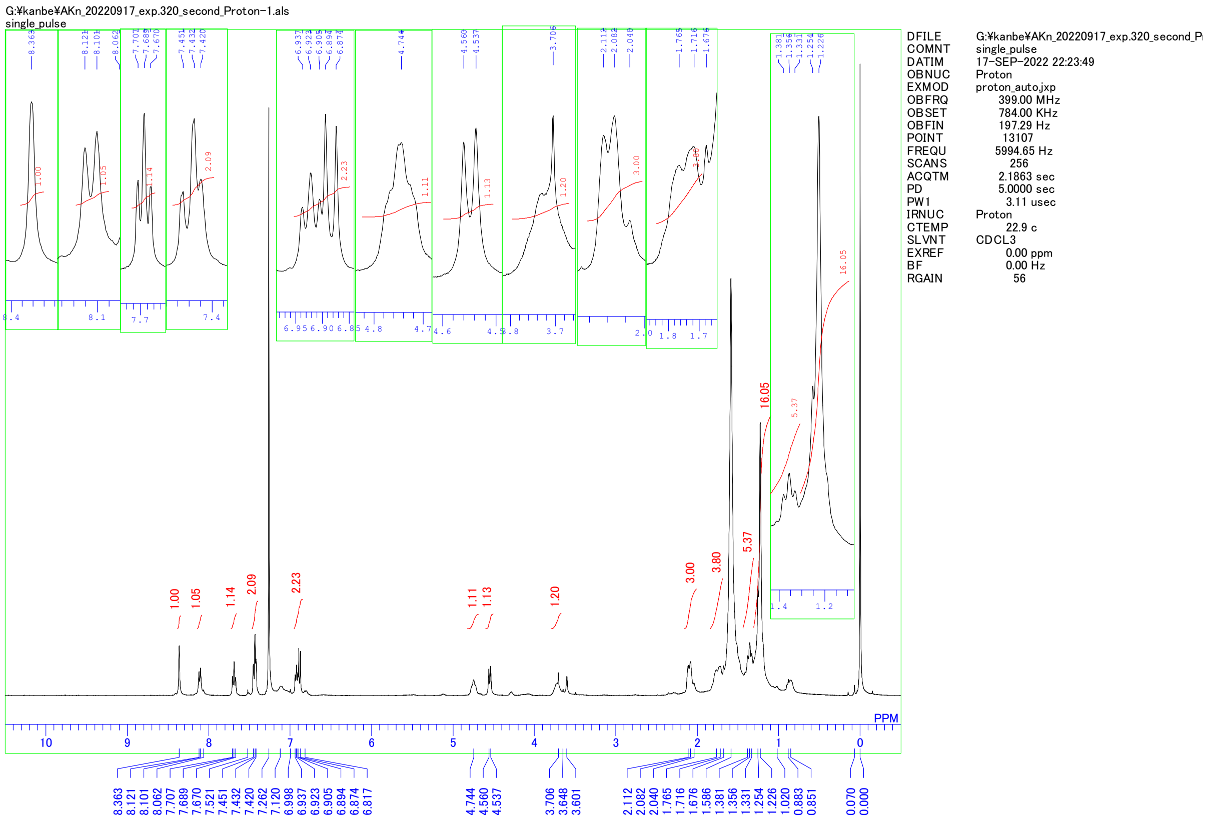

(b) ^{13}C NMR

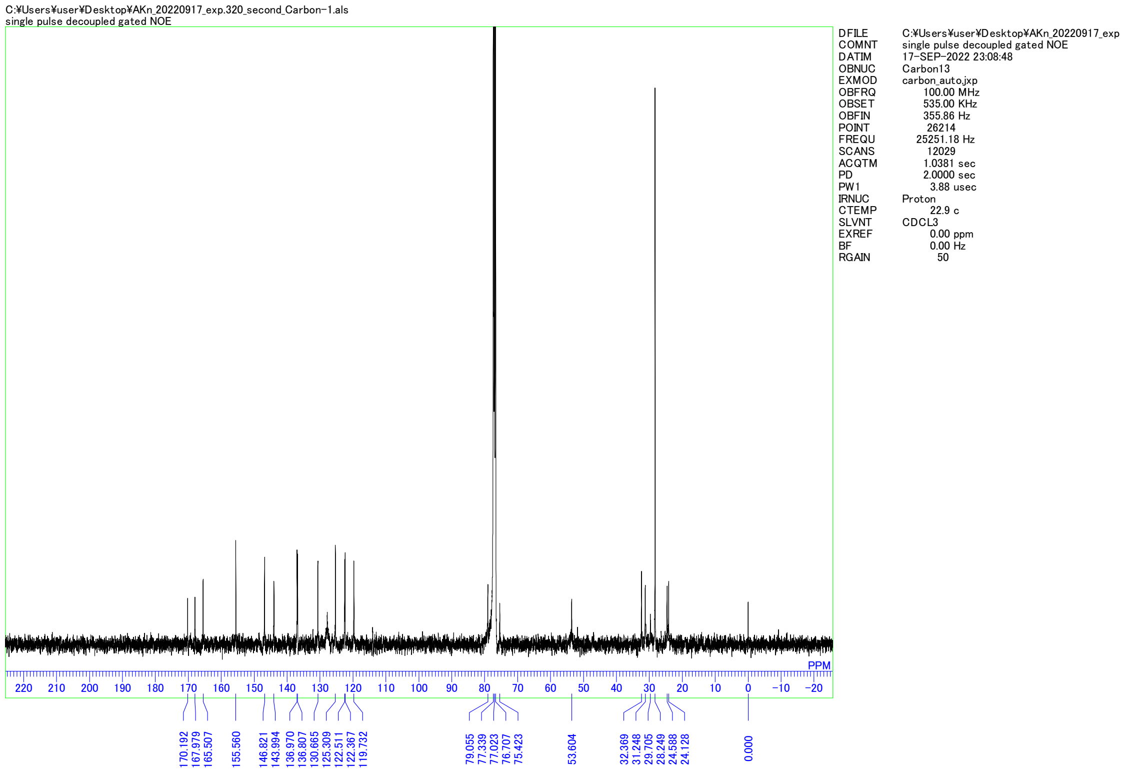

Figure S9. ^{1}H NMR (a) and ^{13}C NMR (b) spectra of *Λ*-*fac*-**10**.

(a) ^{1}H NMR

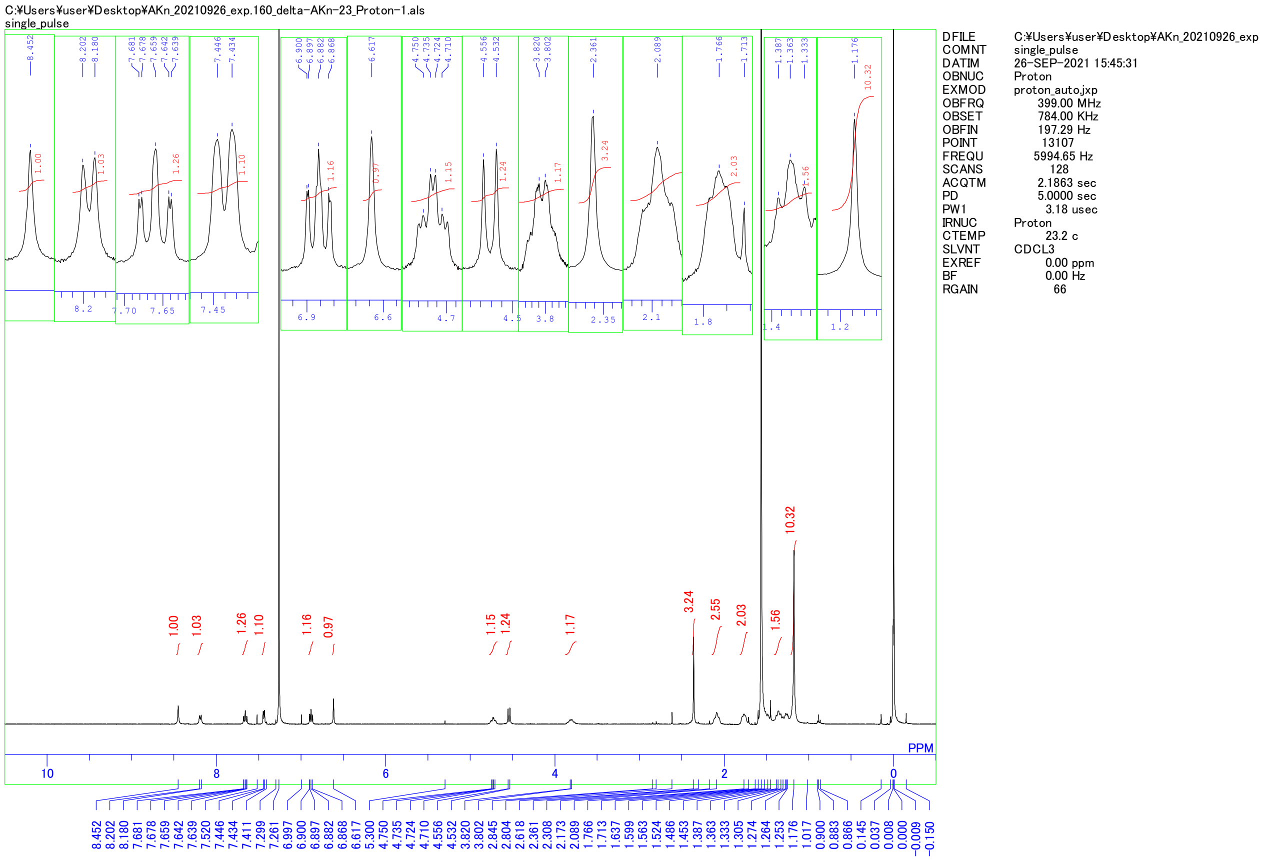

(b) ^{13}C NMR

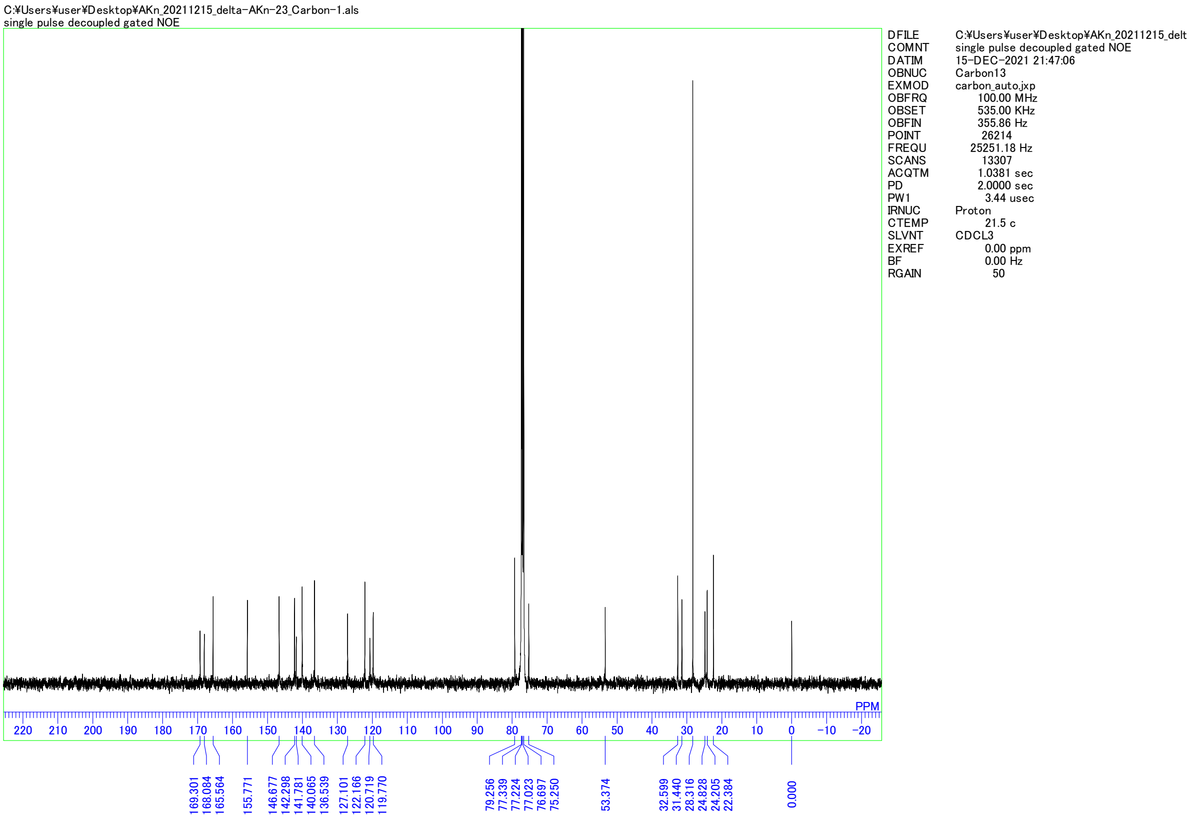

Figure S10. ^{1}H NMR (a) and ^{13}C NMR (b) spectra of *Δ*-*fac*-**11**.

(a) ^{1}H NMR

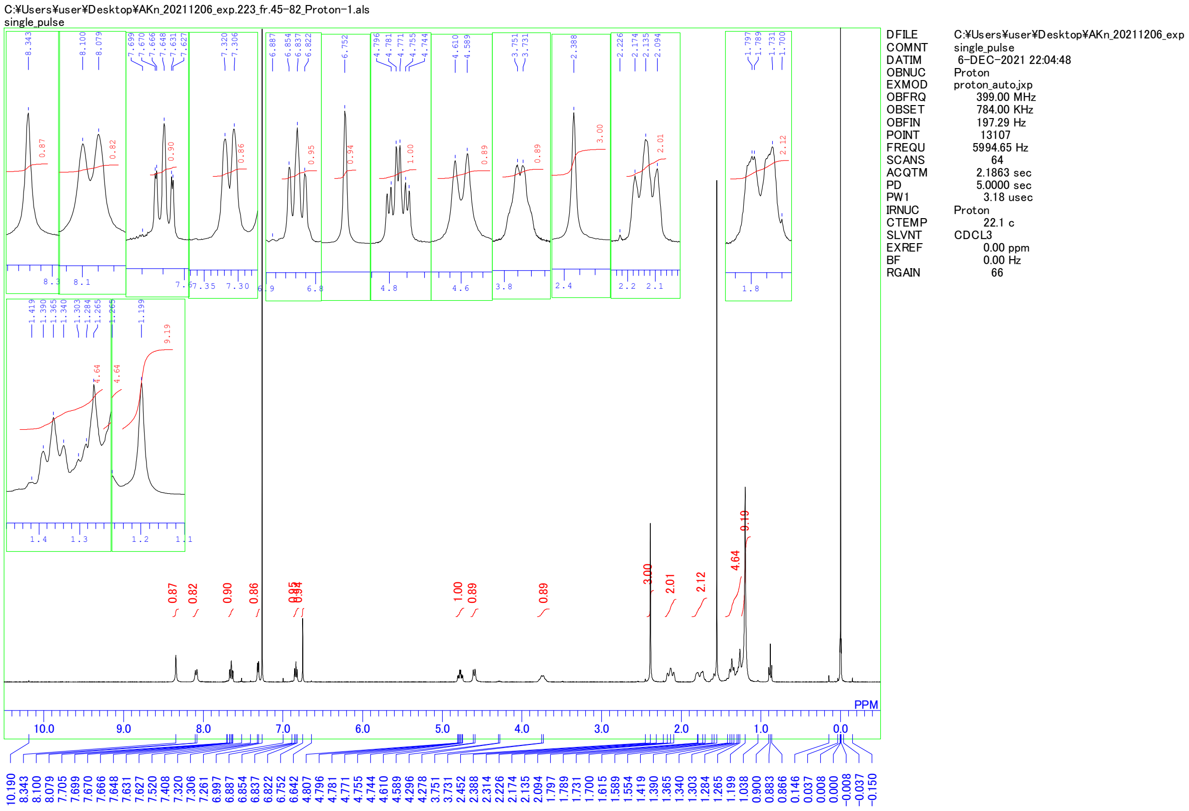

(b) ^{13}C NMR

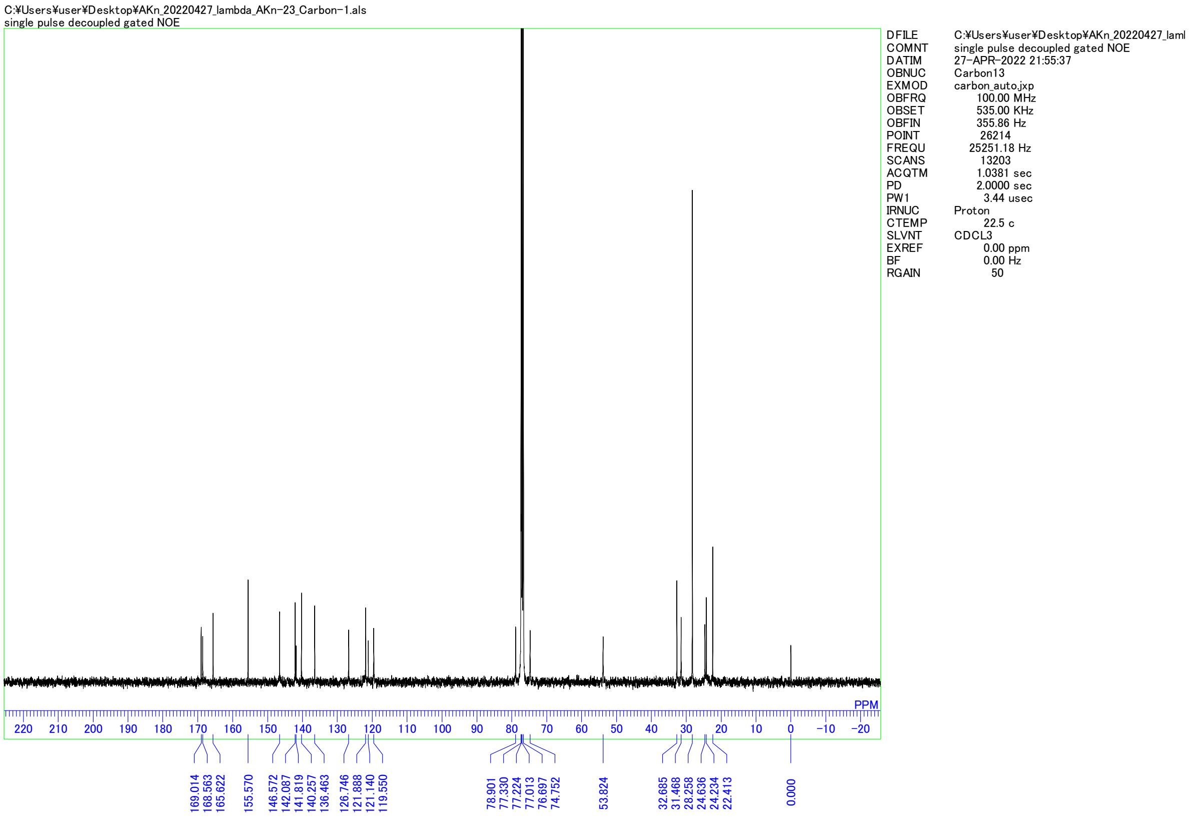

Figure S11. ^{1}H NMR (a) and ^{13}C NMR (b) spectra of *Λ*-*fac*-**11**.

(a) ^{1}H NMR

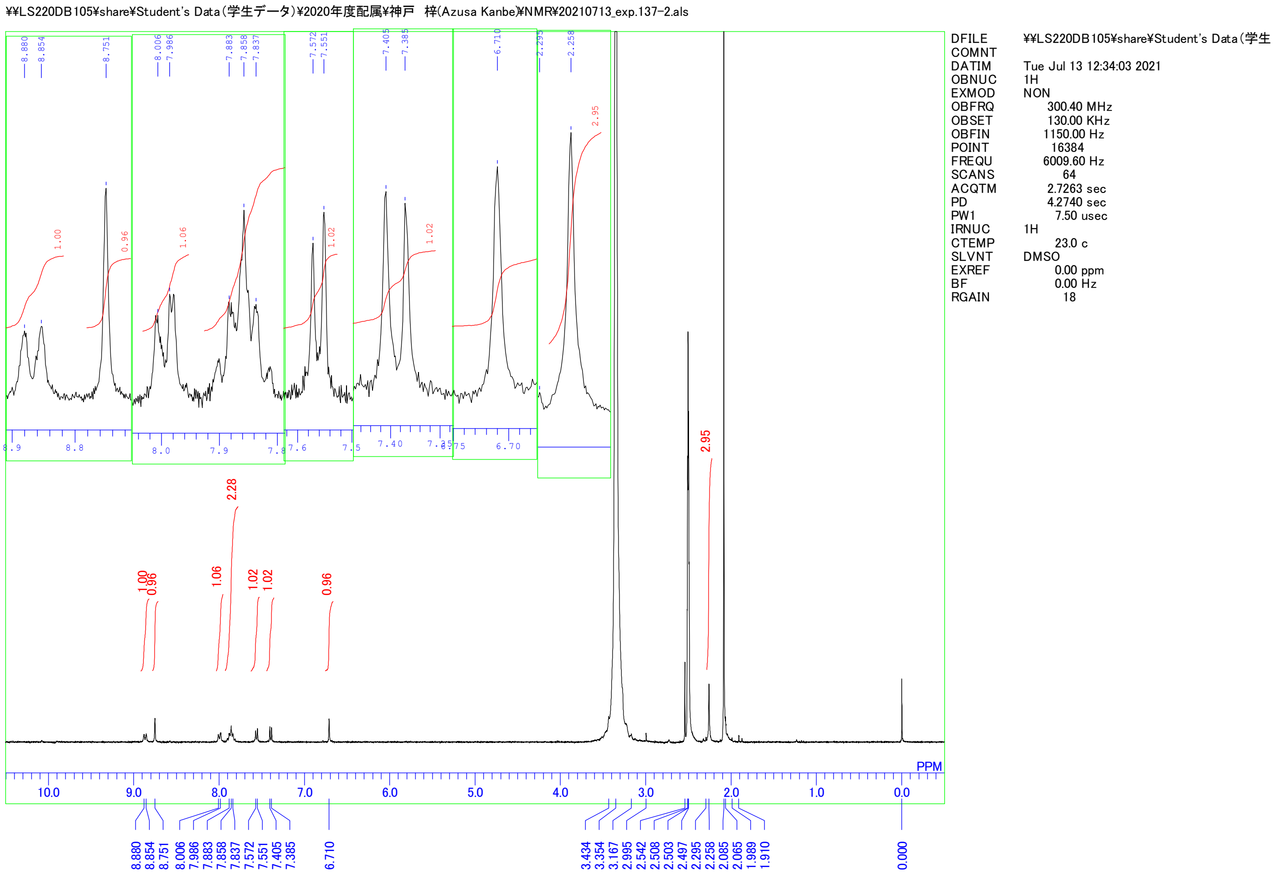

(b) ^{13}C NMR

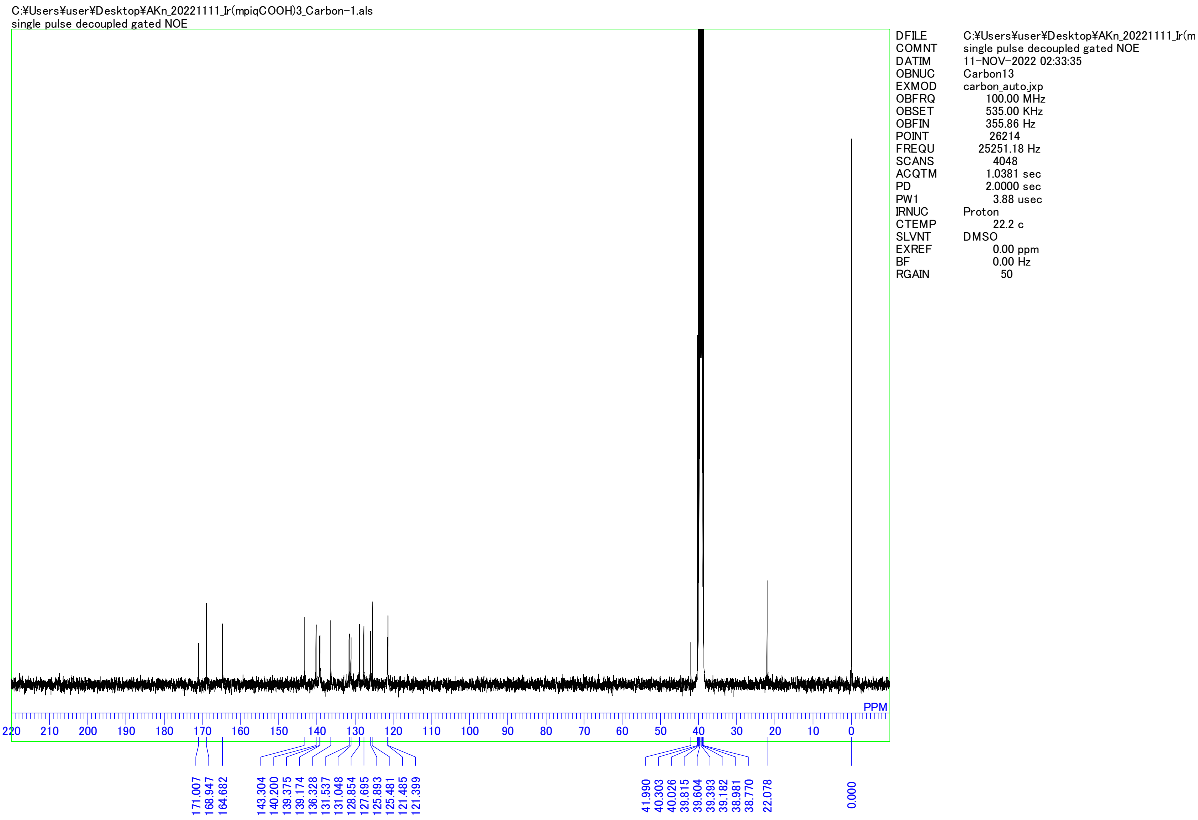

Figure S12. ^{1}H NMR (a) and ^{13}C NMR (b) spectra of racemic *fac*-**13**.

(a) ^{1}H NMR

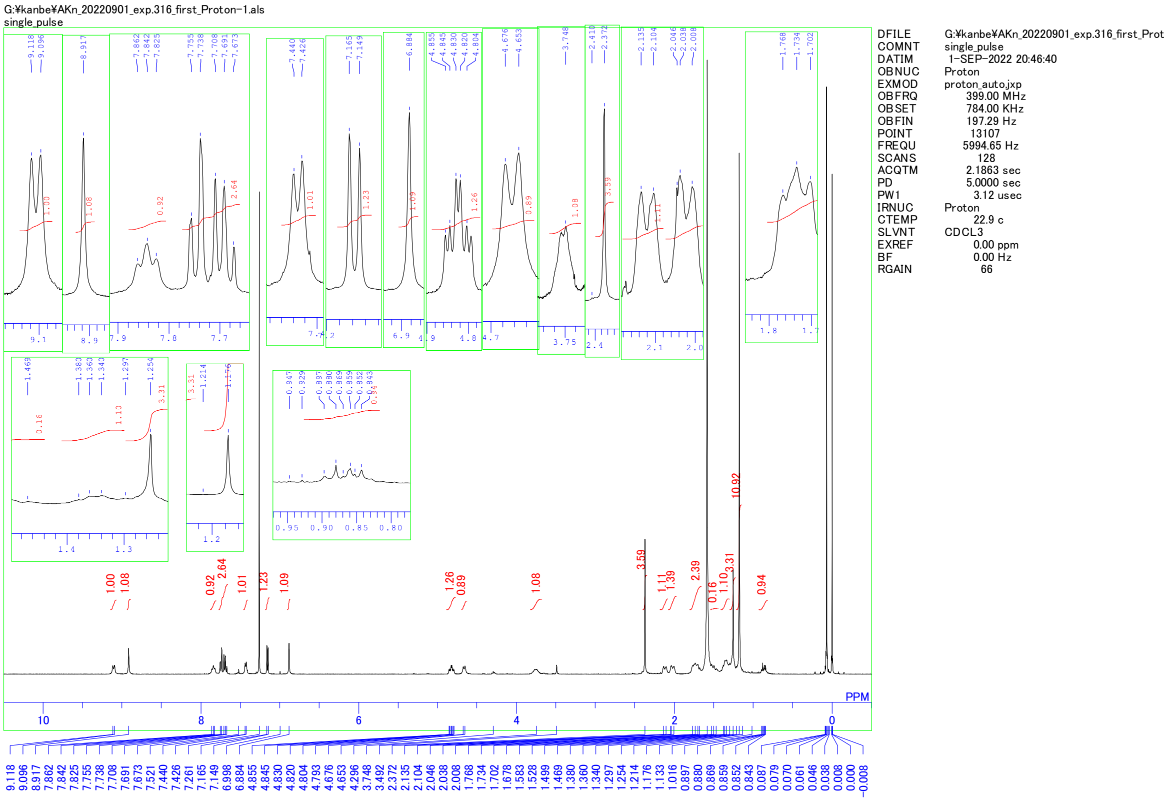

(b) ^{13}C NMR

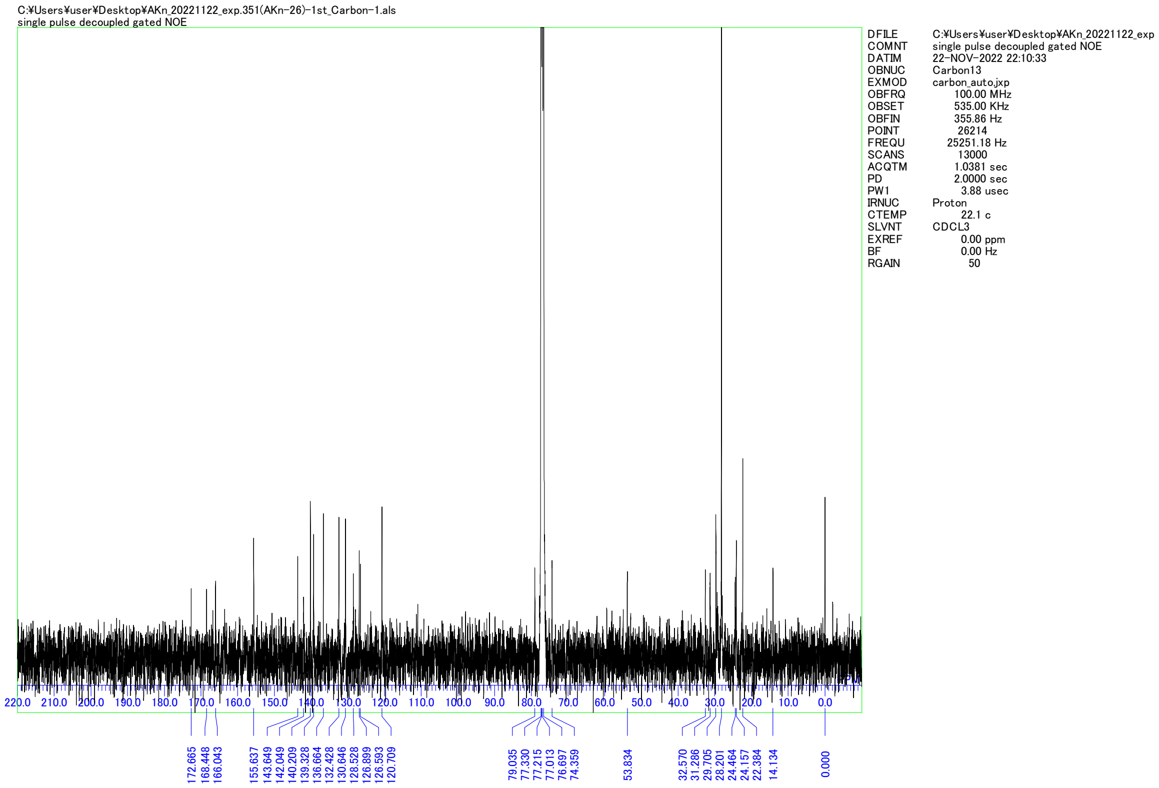

Figure S13. ^{1}H NMR (a) and ^{13}C NMR (b) spectra of *Δ*-*fac*-**14**.

(a) ^{1}H NMR

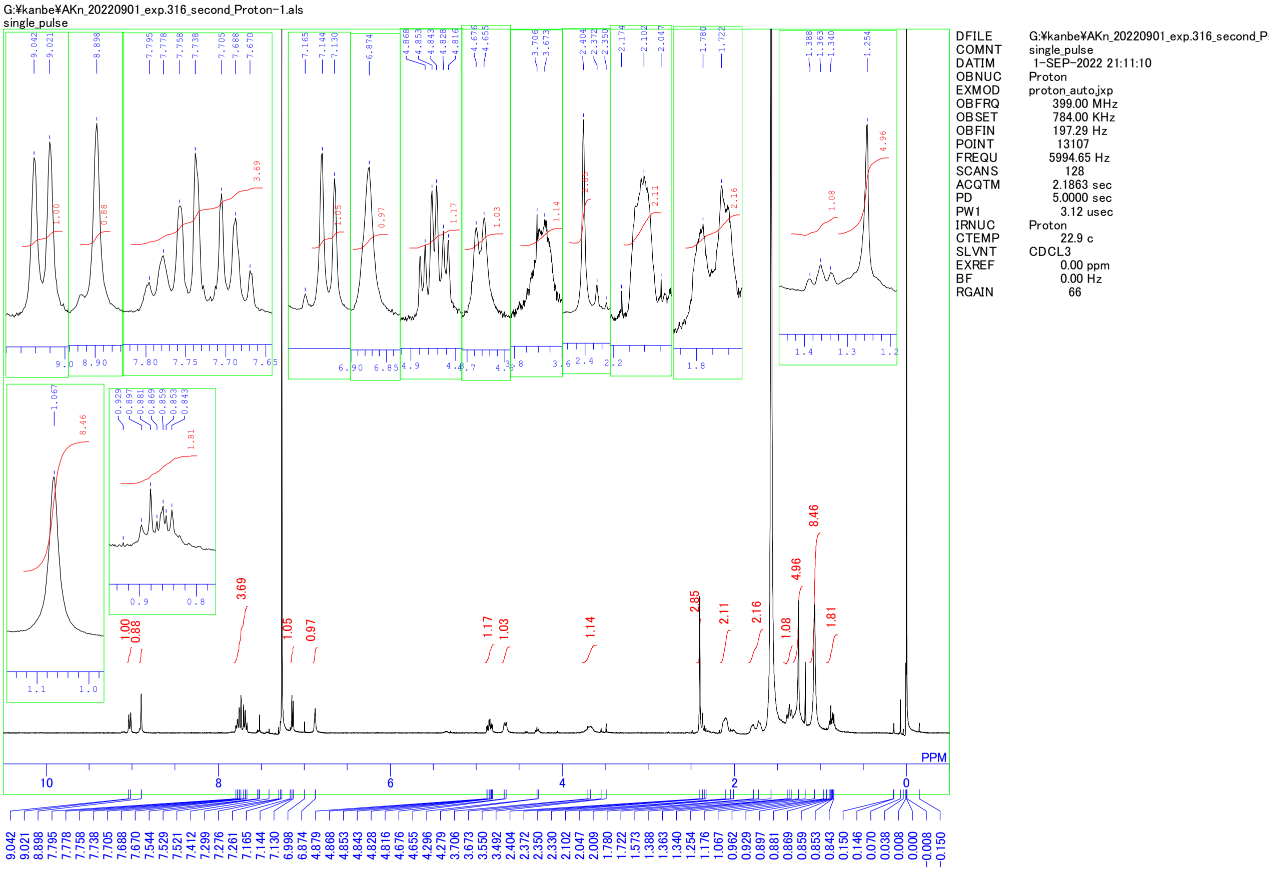

(b) ^{13}C NMR

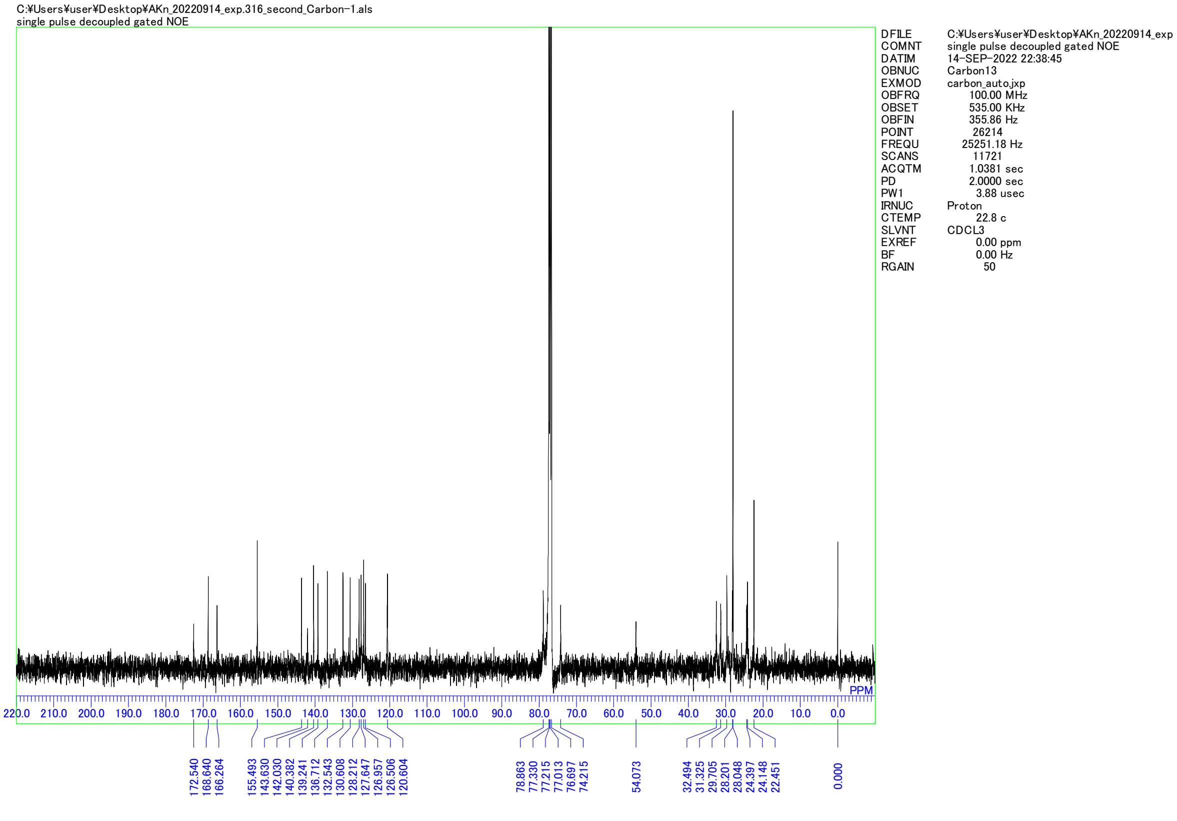

Figure S14. ^{1}H NMR (a) and ^{13}C NMR (b) spectra of *Λ*-*fac*-**14**.

(a) ^{1}H NMR

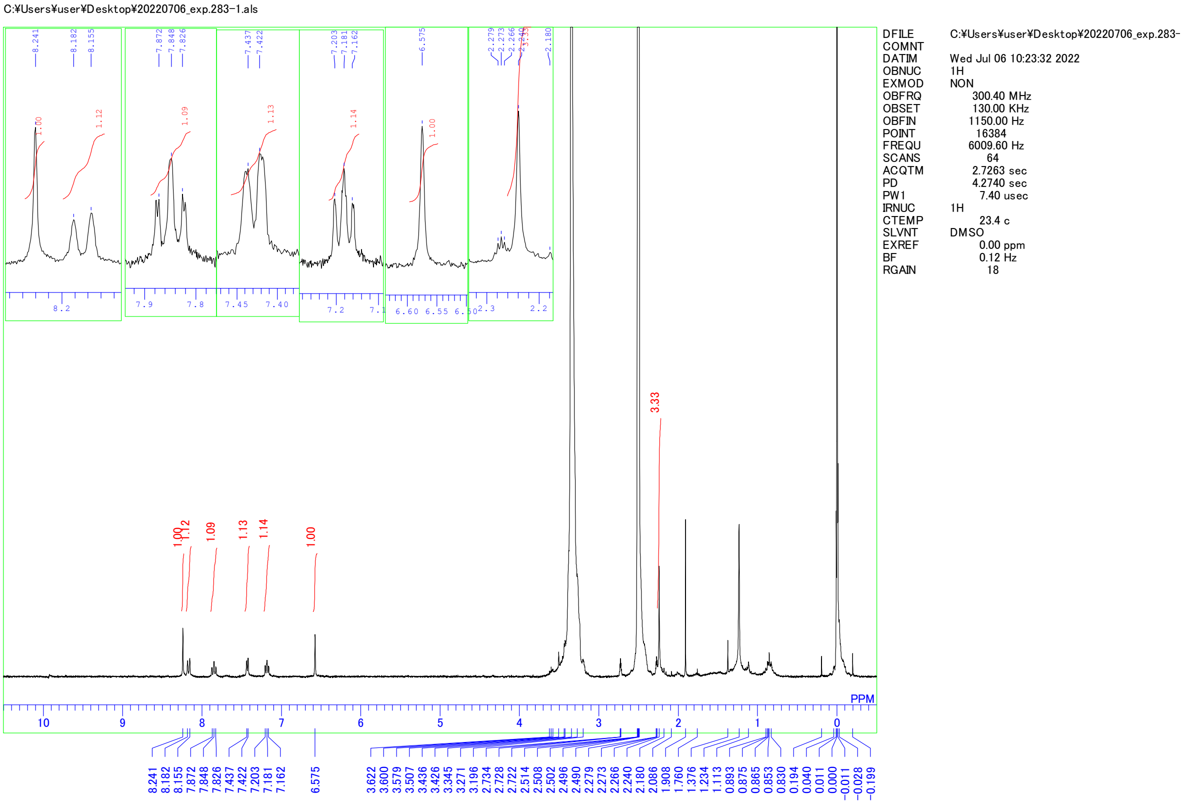

(b) ^{13}C NMR

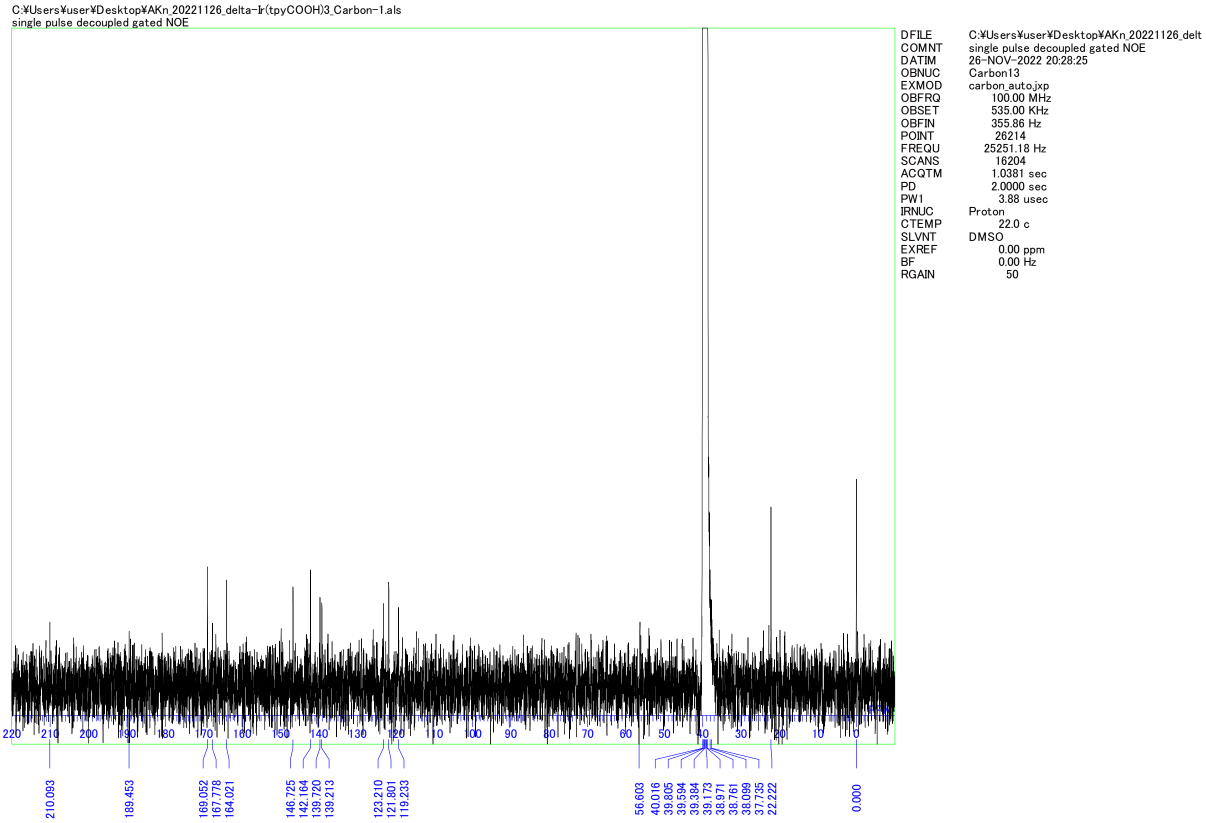

Figure S15. ^{1}H NMR (a) and ^{13}C NMR (b) spectra of *Δ*-*fac*-**6**.

(a) ^{1}H NMR

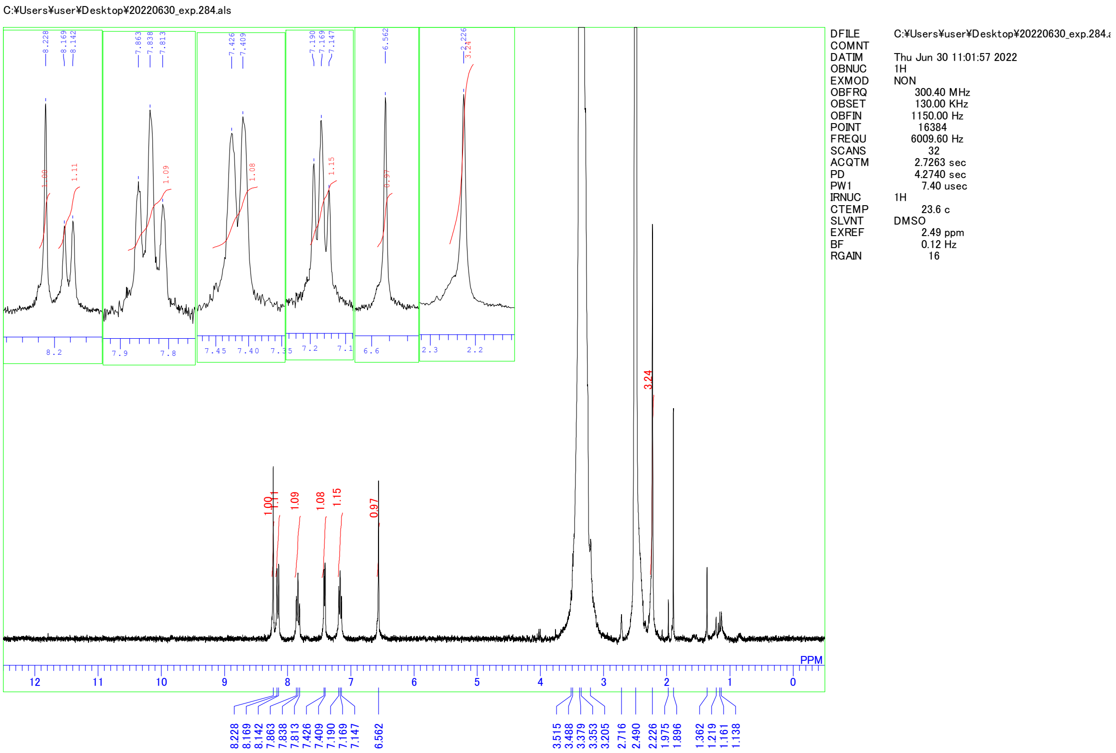

(b) ^{13}C NMR

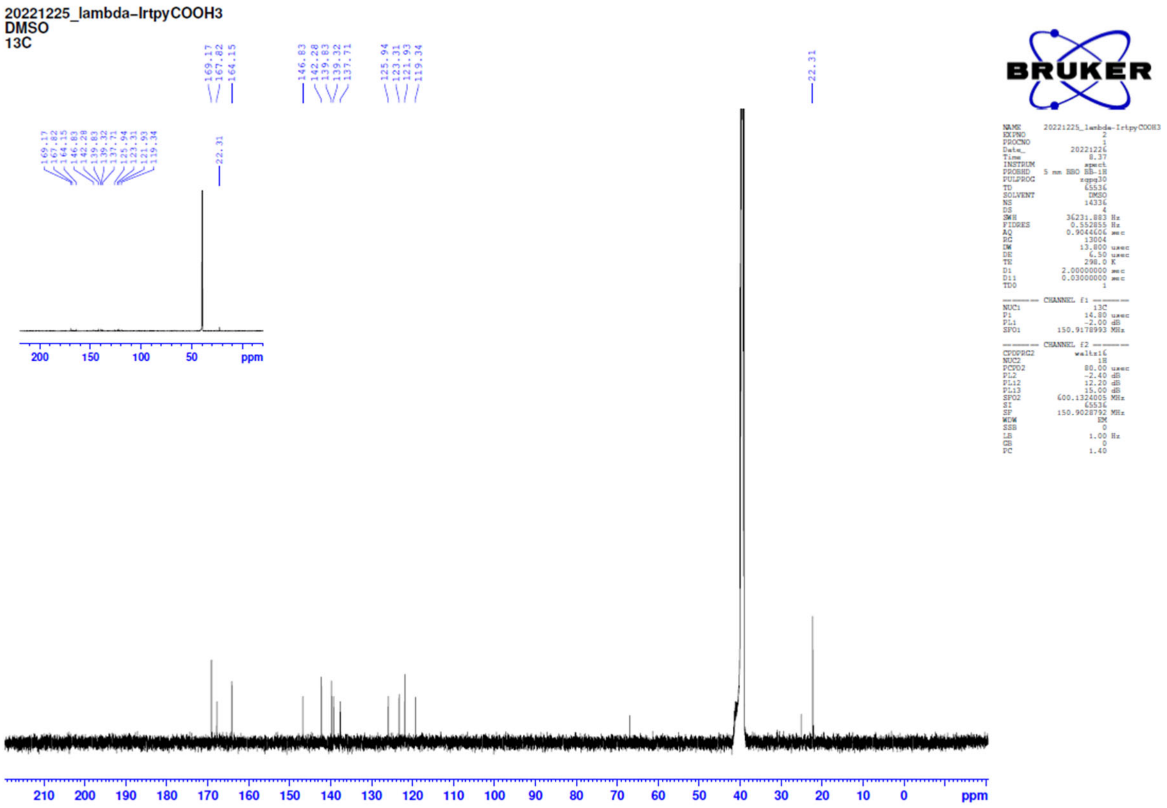

Figure S16. ^{1}H NMR (a) and ^{13}C NMR (b) spectra of *Λ*-*fac*-**6**.

(a) ^{1}H NMR

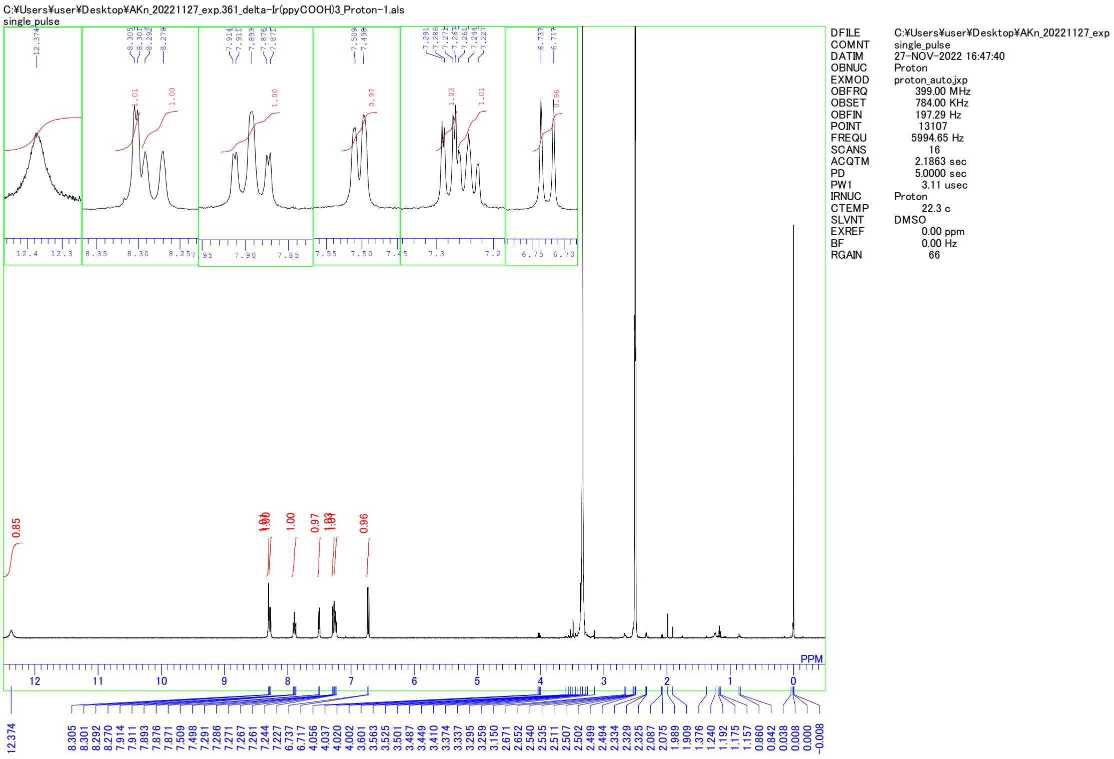

(b) ^{13}C NMR

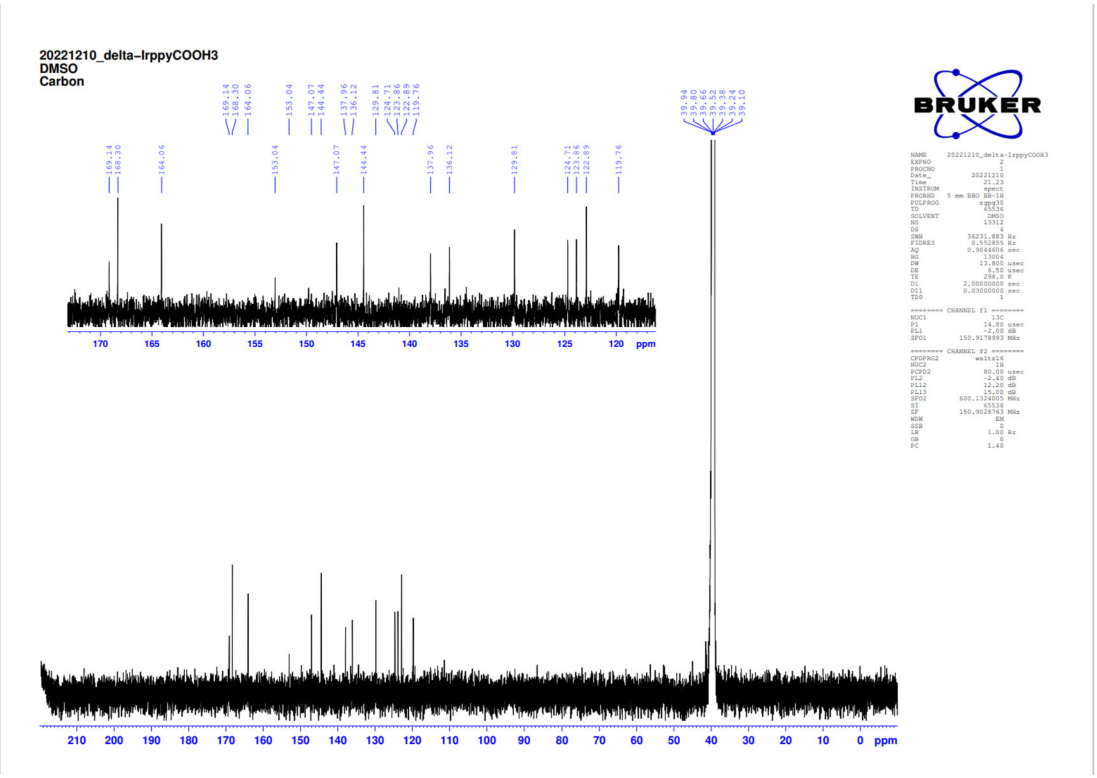

Figure S17. ^{1}H NMR (a) and ^{13}C NMR (b) spectra of *Δ-fac*-**4**.

(a) ^{1}H NMR

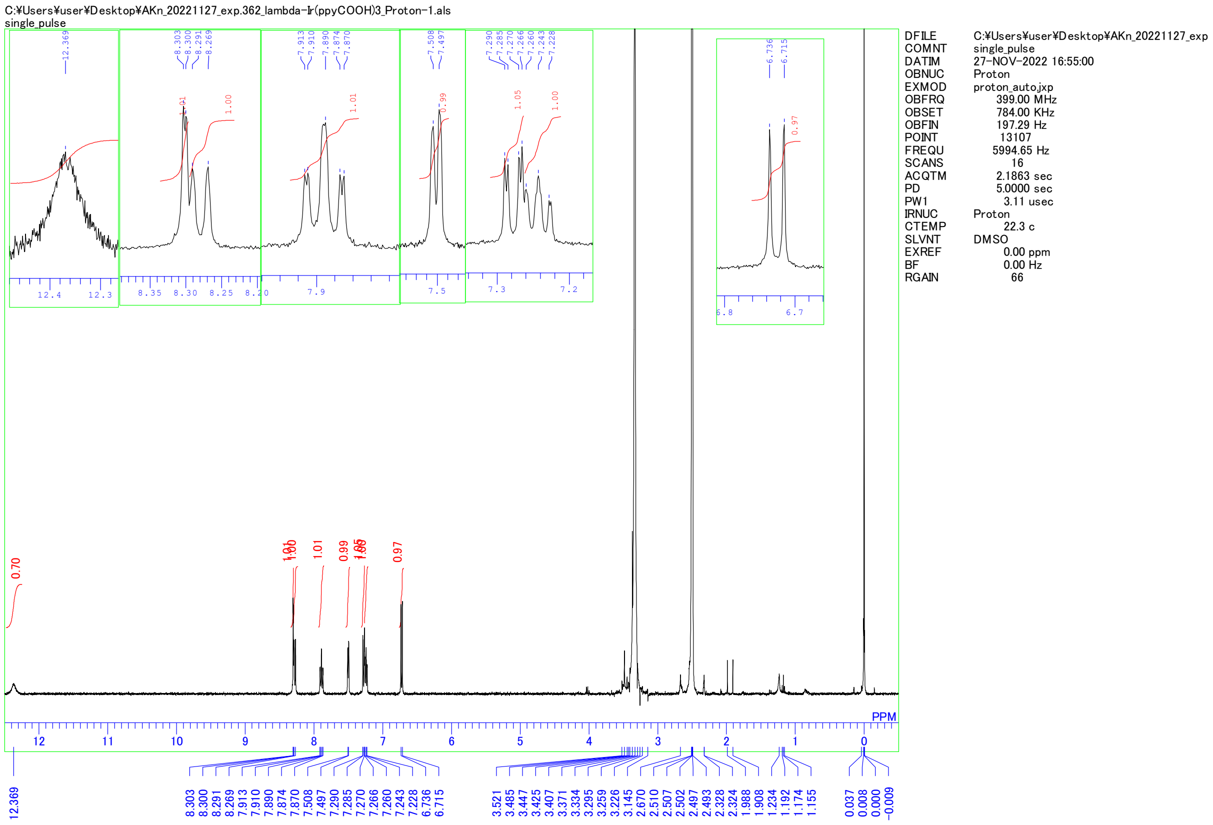

(b) ^{13}C NMR

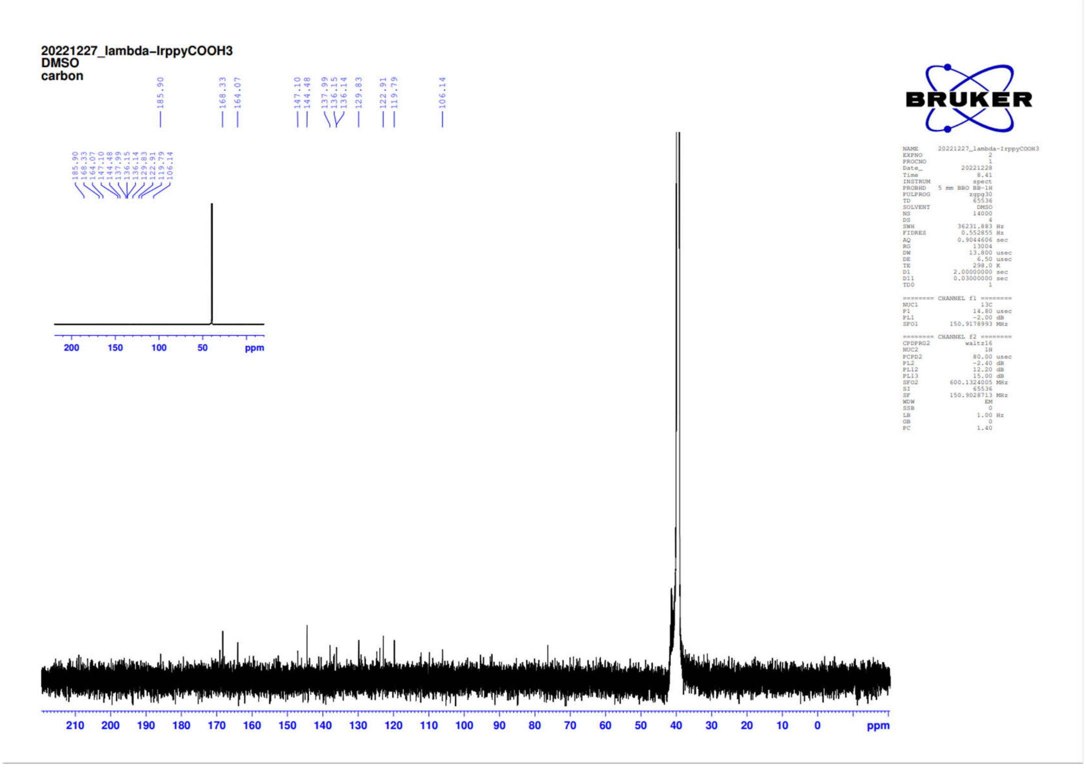

Figure S18. ^{1}H NMR (a) and ^{13}C NMR (b) spectra of *Λ*-*fac*-**4**.

(a) ^{1}H NMR

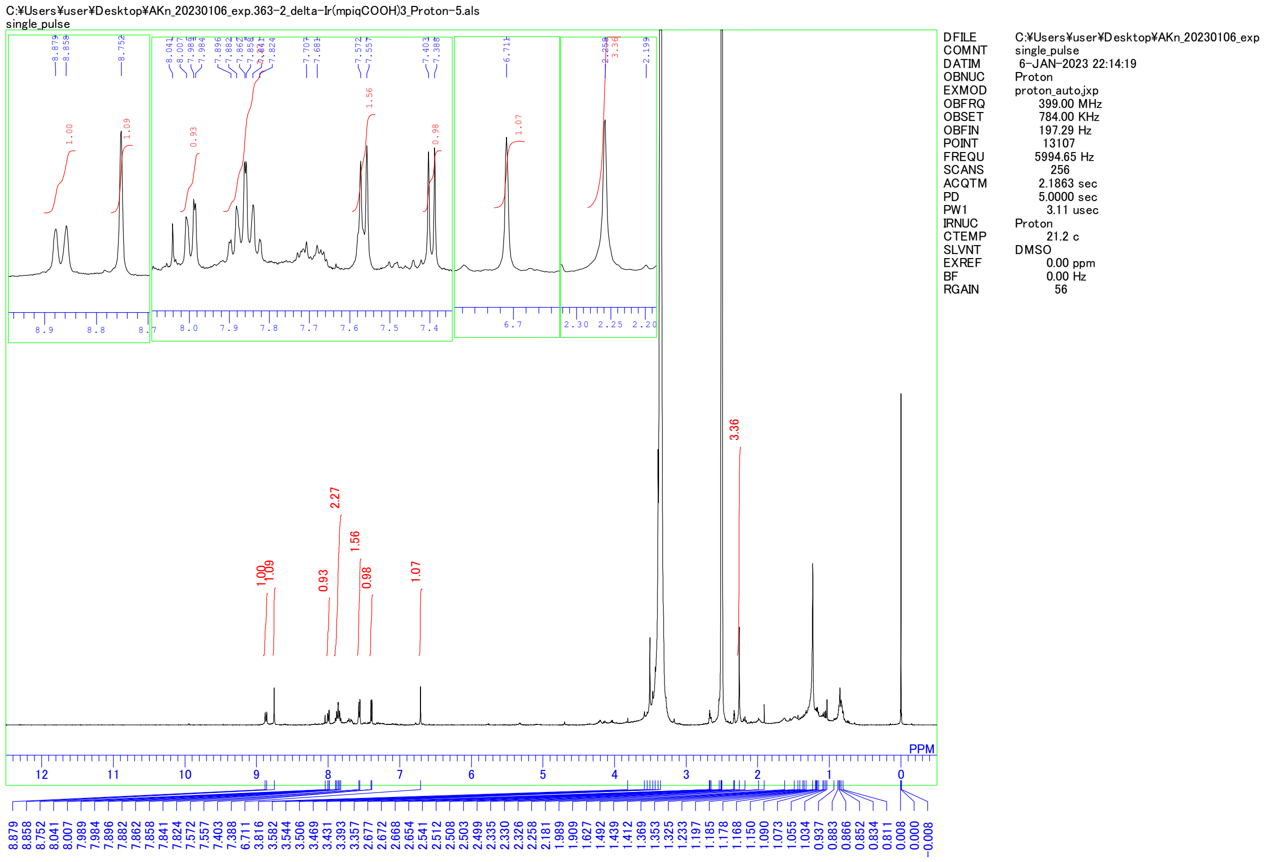

(b) ^{13}C NMR

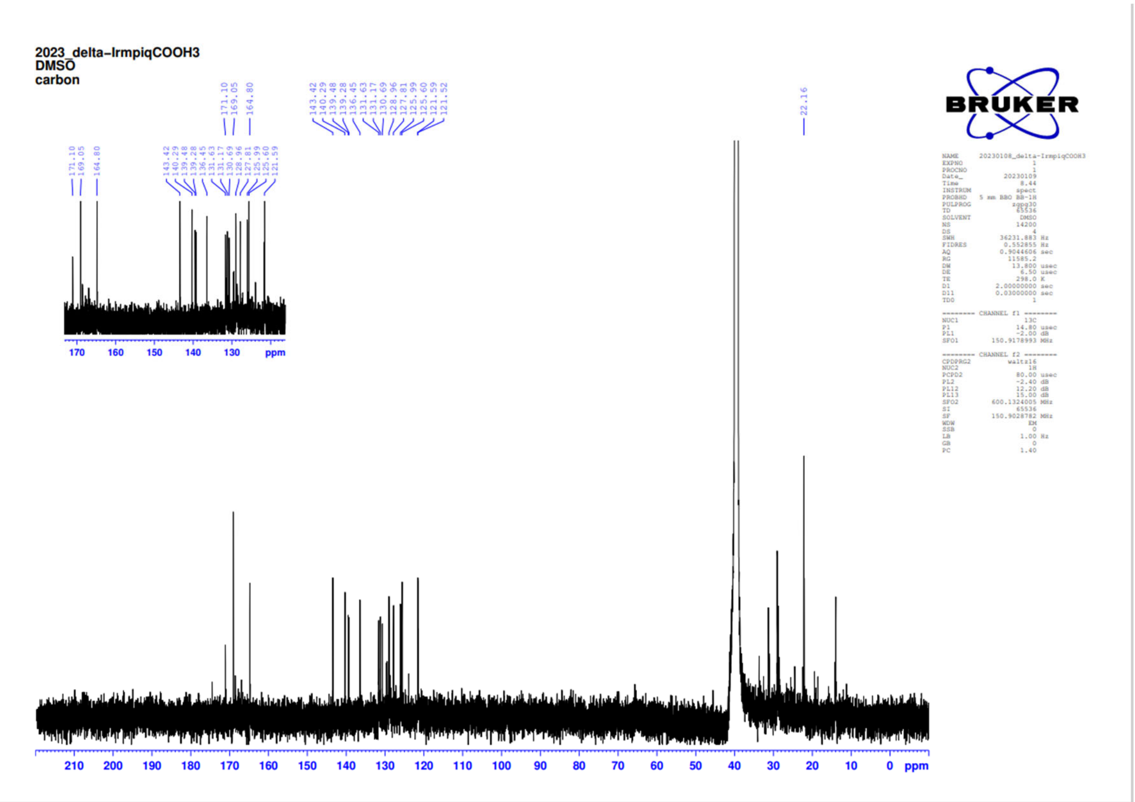

Figure S19. ^{1}H NMR (a) and ^{13}C NMR (b) spectra of *Δ*-*fac*-**13**.

(a) ^{1}H NMR

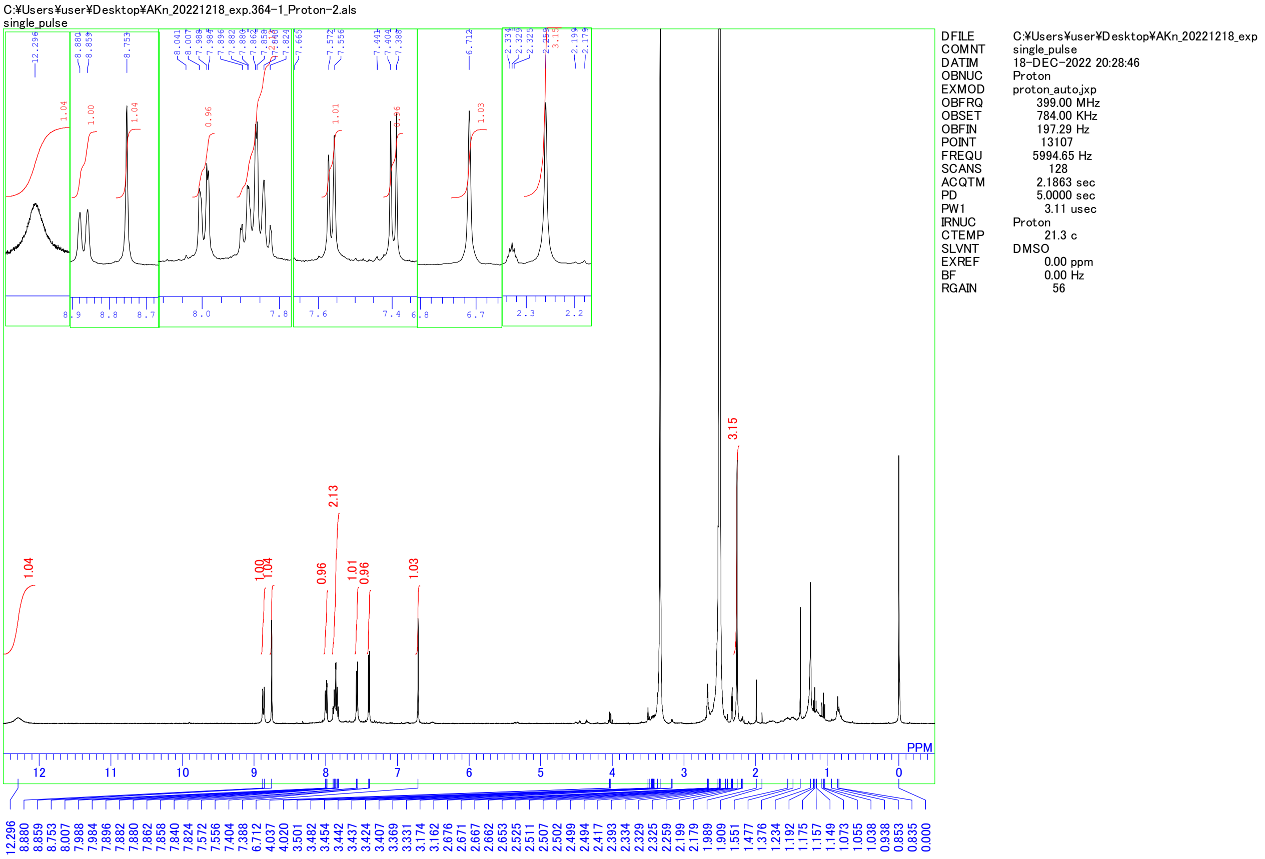

(b) ^{13}C NMR

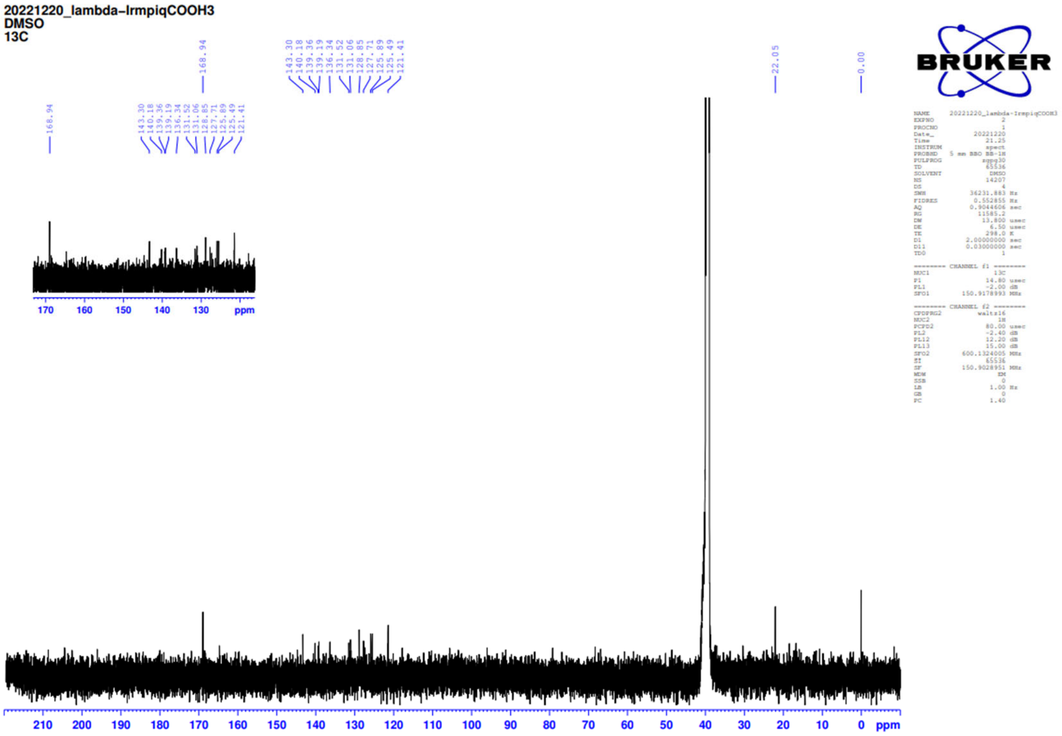

Figure S20. ^{1}H NMR (a) and ^{13}C NMR (b) spectra of *Λ*-*fac*-**13**.
